# Self-packaged stretchable printed circuits with ligand-bound liquid metal particles in elastomer

**DOI:** 10.1038/s41467-025-60118-4

**Published:** 2025-05-28

**Authors:** Hyeonyeob Seo, Gun-Hee Lee, Jiwoo Park, Dong-Yeong Kim, Yeonzu Son, Semin Kim, Kum Seok Nam, Congqi Yang, Joonhee Won, Jae-Young Bae, Hyunjun Kim, Seung-Kyun Kang, Steve Park, Jiheong Kang, Seongjun Park

**Affiliations:** 1 Department of Bio and Brain Engineering, Korea Advanced Institute of Science and Technology (KAIST), Daejeon, Republic of Korea; 2 Departments of Cogno-Mechatronics Engineering, Pusan National University, Busan, Republic of Korea; 3 Departments of Optics and Mechatronics Engineering, Pusan National University, Busan, Republic of Korea; 4 School of Electrical Engineering, Korea Advanced Institute of Science and Technology (KAIST), Daejeon, Republic of Korea; 5 Program of Brain and Cognitive Engineering, Korea Advanced Institute of Science and Technology (KAIST), Daejeon, South Korea; 6 Graduate School of Semiconductor Technology, Korea Advanced Institute of Science and Technology (KAIST), Daejeon, Republic of Korea; 7 Medical Research Center, Seoul National University, Seoul, Republic of Korea; 8 Department of Materials Science and Engineering, Korea Advanced Institute of Science and Technology (KAIST), Daejeon, Republic of Korea; 9 Department of Materials Science and Engineering, Seoul National University, Seoul, Republic of Korea; 10 KAIST Institute for NanoCentury, Daejeon, Republic of Korea; 11 Department of Chemistry, Seoul National University, Seoul, Republic of Korea; 12 Department of Biomedical Sciences, College of Medicine, Seoul National University, Seoul, Republic of Korea; 13 School of Transdisciplinary Innovations, Seoul National University, Seoul, Republic of Korea; 14 Interdisciplinary Program in Bioengineering, College of Engineering, Seoul National University, Seoul, Republic of Korea; 15 Department of Transdisciplinary Medicine, Seoul National University Hospital, Seoul, South Korea

**Keywords:** Electronic devices, Nanoparticles

## Abstract

Packaging in stretchable electronics is crucial to protect components from environmental damage while preserving mechanical flexibility and providing electrical insulation. The conventional packaging process involves multiple steps that increase in complexity as the number of circuit layers multiply. In this study, we introduce a self-packaged stretchable printed circuit board enabled by the in situ phase separation of liquid metal particles (LMPs) within various polymer matrices during solution-based printing processes. The ligand-bound LMPs (LB-LMPs), engineered to inhibit oxide growth, undergo in situ sintering, prompting vertical phase separation. This synthesis strategy not only achieves high initial conductivity of the LMPs but also encapsulates them within the polymer matrix, preventing leakage and providing electrical insulation. Our method enables multi-layer circuit printing, eliminating the need for additional activation and packaging processes. Furthermore, by integrating conductive materials into packaging layers for selective electrical conductivity, vertical interconnect accesses and conductive pads can be formed, enabling large-scale, stretchable, and leakage-free multi-layer electrical circuits and bio-interfaces.

## Abstract (web summary)

Conventional stretchable circuit fabrication requires complex packaging to prevent leakage. Here the authors introduce a self packaging stretchable circuit enabled by in situ sintering of ligand-bound liquid metal particles, allowing scalable, leakage-free, multilayer integration.

## Introduction

Stretchable electronics are becoming increasingly critical for the advancement of future technologies, including wearable devices^1–3^, implantable electronics^4–7^, and soft robotics^8–10^. Especially, stretchable printed circuit boards (S-PCBs) are essential for stretchable electronics by providing a stable and compact platform for mounting and interconnecting electronic components. Traditionally, the fabrication of an S-PCB involves complex processes such as depositing conductive materials, encapsulation packaging (insulating layer coating and patterning), and creating vertical interconnect accesses (VIAs)^11–14^. Ensuring reliable packaging is crucial for the proper functioning of stretchable circuits, as it protects against mechanical stresses and environmental influences, thereby preventing potential short circuits or leakage between conductive traces^15–17^. In addition, reliable VIAs are essential for maintaining electrical connections between circuit layers during dynamic operation^13^. However, the implementation of packaging and VIAs remains underdeveloped in the manufacturing process of S-PCBs. Conventional S-PCB manufacturing involves complex processes wherein VIAs are established by first printing and encapsulating conductors on a board, followed by etching the board or drilling holes in the traces, and subsequently coating or filling them with conductive materials^11,18–23^. This multi-step processes poses obstacles to large-scale integration or the fabrication of delicate circuits on unconventional substrates, such as textiles and clothes, while the process complexity increases significantly as the circuit layers multiply^24^.

To implement an S-PCB, it is also crucial to select circuit materials which maintain constant electrical resistance under stretching. Gallium-based liquid metal particles (LMPs), thanks to the metallic conductivity and exceptional deformability, serve as a prominent choice for stretchable conductive mateirals^25–30^. In contrast to liquid metal, LMPs exhibit enhanced stability and no fluidity due to the presence of a native oxide layer on their surface^26,30^. The oxide layer reduces surface tension, facilitating the formation of composites with the polymer^31^. However, the insulating nature of the oxide layer necessitates an additional sintering process to ensure high conductivity^22,32–36^. This requirement can lead to leakage of liquid metal, attributable to the brittle mechanical characteristics of the LMP oxide layers.

**Fig. 1:**
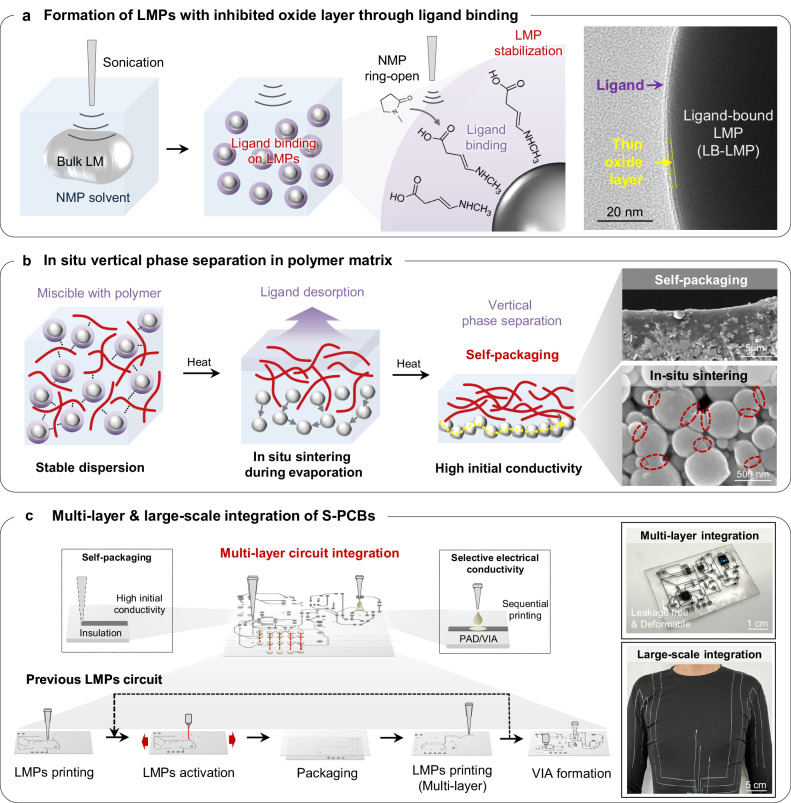
Schematic of ligand-bound liquid metal particles (LB-LMPs) and its application for stretchable printed circuits. **a** Schematic illustration and TEM image of ligands bound on LMP during particle fabrication (4 biological replicates). **b** Schematic illustration and SEM image of in situ sintering and self-packaging of LB-LMPs in a polymer matrix (3 biological replicates).**c** Fabrication of an LB-LMPs-based stretchable multi-layer circuit and large-scale integration.

To address these issues, we propose the concepts of both in situ sintering and self-packaging of conductors through the vertical phase separation of LMPs within various polymer matrices for the fabrication of multi-layer S-PCBs. The utilization of ligand-bound LMPs (LB-LMPs) prompts phase separation within the polymer matrix. When ligands bind to the surface of an LMP, it inhibits the growth of the oxide layer, a phenomenon supported by the Cabrera–Mott theory^37–40^ (Supplementary Fig. 1). We synthesize LB-LMPs using N-Methyl-2-pyrrolidone (NMP) as a solvent, where NMP undergoes ring-opening and attaches ligands to LMP surfaces during sonication to create microparticles (Fig. 1a and Supplementary Fig. 2). The ligand binding is maintained, enabling a stable dispersion of LMPs^41–43^. In addition, we discovered that in LMPs/polymer composites, the ligands on the LMPs are removed and sintered during solvent evaporation (Fig. 1b). The sintered LMPs settle, resulting in a LMP-encapsulation polymer bilayer structure, thereby preventing the leakage of liquid metal during use (Supplementary Fig. 3). This process enables the one-step printing of stretchable circuits, in contrast to conventional methods that require multiple sintering^22,32–36^ and packaging steps^44,45^ (Fig. 1c). Furthermore, selective electrical conductivity can be readily achieved by incorporating conductive materials into the packaging layer, thereby enabling the implementation of VIAs and conductive pads in multi-layer circuits. This approach facilitates the implementation of stretchable multilayer circuits as well as the incorporation of large-scale interconnectors for applications in smart apparel and bioelectronics.

## Results

### Mechanism of in situ sintering of LB-LMPs

**Fig. 2:**
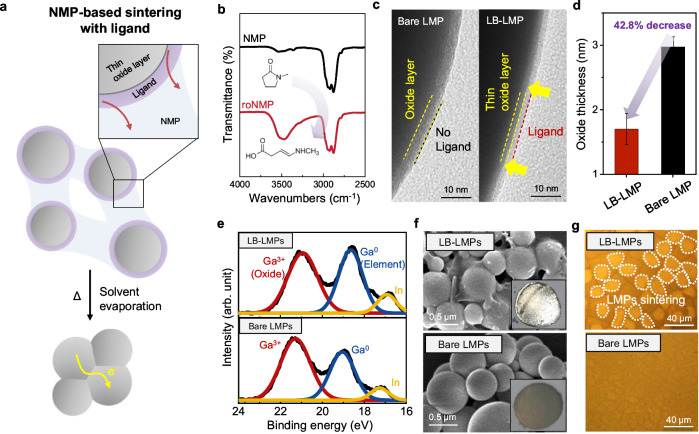
Mechanism of in situ sintering and self-packaging of LB-LMPs. **a** Schematic illustration of the NMP-based sintering process. **b** FTIR spectrum of pure NMP and roNMP. **c**, TEM images of bare LMP and LB-LMP surfaces. **d** Oxide layer thickness of bare LMPs and LB-LMPs (*n* = 12, 4 biological replicates). Values are presented as means ± SD. **e** XPS spectrums of Ga *3 d* of bare LMPs and LB-LMPs surfaces. **f** SEM images of the top surfaces of bare LMPs and LB-LMPs (3 biological replicates). **g** Optical microscope images of the bottom surface of bare LMPs and LB-LMPs (3 biological replicates).

To achieve in situ sintering, LMPs with a thin oxide layer were synthesized by introducing the amine-based ligand (Fig. 2a). LB-LMPs are in situ formed during the sonication of liquid metal in NMP solvent. When NMP is subjected to sonication at high temperature, it undergoes ring-opening reactions during thermal activation^46,47^. The Fourier-transform infrared (FTIR) spectrum of sonicated NMP shows an amine peak at around 3500 cm^−1^, which is absent in the pure spectrum of NMP, confirming cleavage of the pyrrolidone ring^46^ (Fig. 2b). In the X-ray photoelectron spectroscopy (XPS) analysis, the presence of the C-N peak at 285.8 eV and the carboxyl bond peak at 288.6 eV in sonicated NMP are newly observed (Supplementary Fig. 4). A proton nuclear magnetic resonance (^1^H NMR) spectrum additionally substantiates the ring-opening phenomenon of NMP through the observation of peaks corresponding to the carboxyl group (Supplementary Fig. 5). The ring-opened NMP (roNMP) acts as a ligand, binding to the surface of LMPs, thereby stabilizing the dispersion of LMPs (Supplementary Fig. 6). In addition, it reduces the thickness of the oxide layer (Fig. 2c). The oxide layer thickness on the LB-LMP surface experiences a reduction of approximately 42.8% compared to the bare LMP, as depicted in Fig. 2d. To quantify the proportion of oxide layer in LMPs, the XPS spectrum, specifically Ga *3 d*, for both bare LMPs and LB-LMPs were measured (Fig. 2e). In the Ga 3 *d* spectrum of the LB-LMPs, the Ga^0^ peak (18-19 eV) was dominant over the Ga^3+^ peak (~ 21 eV), which constitutes the gallium oxide layer. Conversely, in the case of bare LMPs, the Ga^3+^ peak dominates over the Ga^0^ peak.

To understand which functional groups of roNMPs can effectively inhibit the growth of the LMP oxide layer, we screened ligands with various functional groups in a hydrocarbon solvent and measured the oxide thickness. Upon the introduction of a ligand featuring an amine group, a reduction in the oxide layer by approximately 28.5% was noted. Conversely, the presence of a ligand endowed with a carboxyl group resulted in a slight augmentation of the oxide layer. (Supplementary Figs. 7 and 8). In the XPS spectrum of Ga 3 *d*, only the ligand with an amine functional group had a Ga^0^ peak dominant over the Ga^3+^ peak (Supplementary Fig. 9). From these results, we have confirmed that the bond between the LMP surface and the amine group of roNMP is dominant, and the growth of the oxide layer is suppressed by the amine group. According to the Cabrera-Mott theory, the ligand attached to the LMP surface creates a physical barrier and weakens the electric field between Ga^3+^ and O^2-^, preventing the further oxidation of gallium^39,40^. Especially, the amine group can coordinate with gallium, suppressing the oxidation of gallium^48,49^.

LB-LMPs, in which oxide layer formation is suppressed by roNMP, undergo in situ sintering during the solvent evaporation. To validate the sintering process of LMPs, the surface of the LMPs following solvent evaporation was examined using SEM imaging. The LB-LMPs surface displays a shiny surface, with localized sintering of particles observed through the SEM image (Fig. 2f). In addition, in the optical image of the bottom surface of LB-LMPs, clusters indicate that LMPs sintering are clearly confirmed, a feature notably absent in the case of bare LMPs (Fig. 2g).

### In situ sintering and self-packaging of LB-LMPs in polymer matrices

**Fig. 3:**
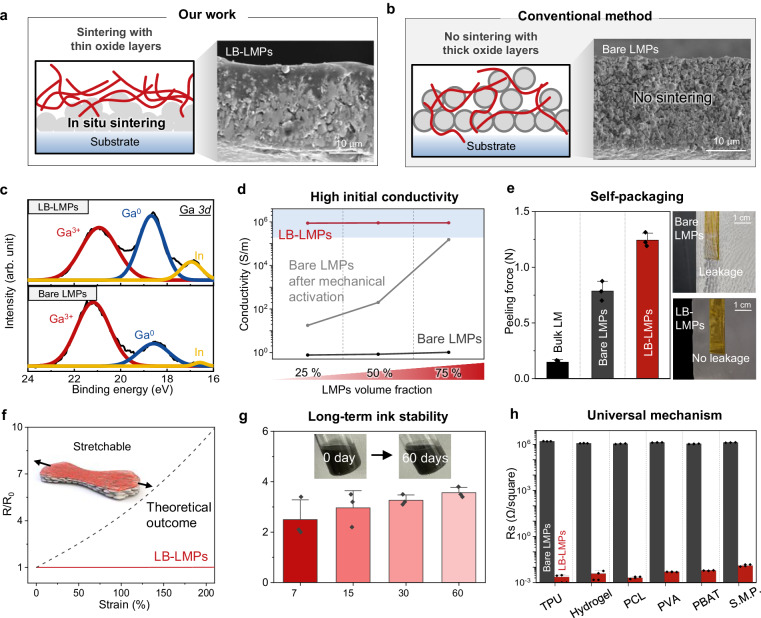
Mechanical and electrical properties of LB-LMPs-based conductors. **a** Schematic illustration and cross-sectional SEM image of the LB-LMPs/polymer film and (**b**) the bare LMPs/polymer film (3 biological replicates). **c** XPS of Ga 3 d on the bottom surface of the LMPs/polymer films. **d** Conductivity of bare LMPs and LB-LMPs-based conductors according to polymer contents. **e** Peeling force of bulk LM, bare LMPs/polymer and LB-LMPs/polymer films (*n* = 3 biologically independent samples). Values are presented as means ± SD. There is no leakage of the LB-LMPs/polymer film after peel off with tape. **f** Relative resistance of LB-LMPs under applications of strain. **g** Resistance of LB-LMPs films according to the day after ink formation (*n* = 3 biologically independent samples). Values are presented as means ± SD. **h** Sheet resistance of bare LMPs and LB-LMPs in various polymer matrices (*n *= 3 biologically independent samples). Values are presented as means ± SD.

The in situ sintering of LB-LMPs persists even when they coexist with a polymer matrix (Fig. 3a). Featuring thin oxide layers, LB-LMPs undergo in situ sintering within the polymer and subsequently sink downwards, leading to the upward separation of the polymer phase. In the SEM image, the film’s lower segment shows connectivity with LMPs, forming a tight conductive pathway, while the upper region is packaged by polymers, resulting in self-packaging. This vertical phase separation effectively prevents the leakage of liquid metal while maintaining initial conductivity, eliminating the need for additional activation and packaging processes. In contrast, bare LMPs with thick oxide layers failure to sinter or achieve complete phase separation in the polymer matrix (Fig. 3b and Supplementary Fig. 10). In the Ga 3 *d* spectrum, the prevalence of the Ga^0^ peak is predominantly observed on the bottom surface of the LB-LMPs/polymer film, whereas the Ga^3+^ peak is significantly more dominant in the bare LMPs/polymer film, verifying that thick oxide layers interfere with sintering in the polymer matrix (Fig. 3c). Moreover, fabricating the LB-LMPs/polymer film at ambient temperature without thermal treatment precludes ligand detachment, thereby impeding sintering^50–52^ between LMPs and inhibiting phase separation (Supplementary Fig. 11). XPS analysis of LB-LMP/polymer films without heat treatment reveals a peak at 399.5 eV in the N 1 *s* spectrum, indicating incomplete ligand desorption (Supplementary Fig. 12). This suggests that evaporation-induced desorption of ligands from LMPs is crucial to facilitate interparticle sintering among LMPs and initiate vertical phase separation. Notably, sintering of particles allows for high electrical conductivity, achieving over 8.75 × 10^6 ^S/m in the interconnect without requiring activation, even at small LMPs volume ratios (Fig. 3d). In contrast, bare LMPs show low electrical conductivity, even after undergoing rigorous mechanical activation (under 180 S/m at 25% v/v and 200 S/m at 50% v/v).

Packaging is vital in an S-PCB for enhancing the durability of circuits by protecting the circuits from both mechanical stress and environmental influences. Compared to bulk liquid metals and bare LMPs/polymer films, the LB-LMPs/polymer film exhibits relatively high peeling force, experiencing no rupturing or leakage (Fig. 3e and Supplementary Fig. 13). In contrast, the bare LMPs/polymer film displayed multiple instances of rupturing and leakage, resulting in reduced peeling force. LB-LMPs also preserve the deformable characteristics of LMPs, making them ideal for use in interconnects or electrodes by maintaining their resistance under strain (Fig. 3f and Supplementary Fig. 14). In addition, the ligands bound to the LMPs suppress oxidation over an extended period of time, enabling long-term usage of the ink and enhancing its practicality in manufacturing processes. Even after 60 days, the oxide layer thickness of the LB-LMPs showed no significant increase compared to the bare LMPs counterpart (Supplementary Figs. 15 and 16), resulting in LB-LMPs conductor maintaining low resistance in the long-term after from the initial ink formulation (Fig. 3g). Furthermore, by tuning the viscosity of the ink through varying polymer contents, various solution process like printing or dip coating become available (Supplementary Figs. 17 and 18). Even upon dip coating of the fiber with LB-LMPs/polymer ink, sintering of LMPs occurred, followed by polymer packaging on the external surface, preventing leakage of liquid metal under strain (Supplementary Figs 19 and 20). This utilization broadens the application scope of LB-LMPs to include smart textiles with electronic fibers. Since the in situ sintering of LB-LMPs relies on a universal mechanism involving ligand binding, it is compatible with a variety of polymers. High electrical conductivity is achieved with various polymers that can dissolve in the NMP solvent, such as thermoplastic polyurethane (TPU), polycaprolactone (PCL), and polyvinyl alcohol (PVA), demonstrating the versatility of LB-LMPs-based conductors (Fig. 3h). A small amount of LB-LMPs inclusion in polymer matrix even allows it to maintain its original mechanical properties (Supplementary Fig. 21).

### Developing vertical electrical conductivity for stretchable electronics

**Fig. 4:**
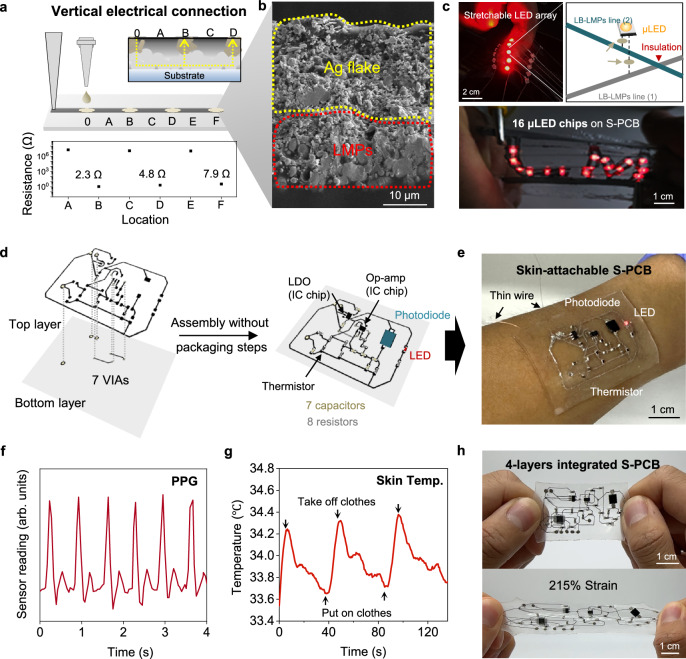
LB-LMPs-based multi-layered stretchable circuit. **a** Schematic illustration and resistance of the selectively electrically connected LB-LMPs-based conductor with silver flakes. **b** SEM image of selective electrical connections after printing silver flake composites (3 biological replicates). **c** Optical images and schematic illustration of the LED array under strain. **d** Schematic illustration of the S-PCB with conductive traces, VIAs, and electronic components using LB-LMPs for PPG and temperature sensing. **e** Optical image of the PPG sensor attached to human skin. **f** The normalized reading of the PPG sensor on the wrist. **g** Measured skin temperature change on the wrist. **h** Optical image of a stand-alone 4-layer integrated S-PCB (top) and the S-PCB under 215% strain (bottom).

In the pursuit of completing electrical systems, mechanically robust electrodes are essential for seamless integration with electronic components or bioelectronic interfaces^53–55^. However, physically removing the packaged polymer layer inevitably leads to the leakage of liquid metal. To address this issue, we developed a strategy that enables vertical electrical conductivity of the packaged polymer. This was achieved by applying a conductive material, such as silver flakes (AgF), after printing the LB-LMPs/polymer (Fig. 4a). The high-density AgF ink creates an electrical pathway from the bottom LB-LMPs layer to the top layer (Fig. 4b**and** Supplementary Fig. 22). Consequently, electrical conductivity is present only in the regions where the AgF is applied, while the other areas remain insulated, making the exposed area a connection region (Supplementary Figs. 23 and 24). Unlike LMPs, the AgF is stable, allowing for robust integration with other components without any rupturing or leakage, even in the absence of packaging^56,57^ (Supplementary Fig. 25). The fabricated conductor demonstrates mechanical and electrical stability, maintaining its initial resistance after 10,000 cycles of 100% strain (Supplementary Fig. 26). Furthermore, we fabricated a stretchable display by utilizing LB-LMPs-based conductors, highlighting their advantages of self-packaging and selective electrical connections with LED chips (Fig. 4c). To manage the LED arrays effectively, a precise design of electrical voltage without interference is essential, necessitating a multi-layer circuit for both voltage and ground. In situ packaging via LB-LMPs printing instantly produces circuits with two layers, avoiding any electrical shorts. In addition, the electrical interconnection of selective AgF establishes a pad suitable for integration with LED chips.

The inherent initial conductivity, self-packaging, and vertical electrical connection capabilities of the LB-LMPs composites facilitate the demonstration of various deformable and stretchable circuits in diverse configurations. In modern electronics, multi-layer circuits are crucial for the precise control of electronic components, ensuring operation free from electrical interference^13,20,24^. The use of LB-LMPs composite in printing enables the creation of self-packaged circuits, while its selective electrical connection capability allows for the seamless integration of VIAs and conductive pads (Fig. 4d). Through the integration of VIAs and pads, we developed an S-PCB capable of performing both photoplethysmography (PPG) and temperature sensing (Fig. 4e). The healthcare monitoring S-PCB consistently measured PPG and body temperature under dynamic stress applied to the skin (Fig. 4f, g and Supplementary Fig. 27). In addition, the 4-layer integrated S-PCB comprising 26 chips and 24 VIAs remained leakage-free under 215% strain (Fig. 4h and Supplementary Figs. 28 and 29).

### Scalable integration of LB-LMPs for smart apparels and implantable devices

**Fig. 5:**
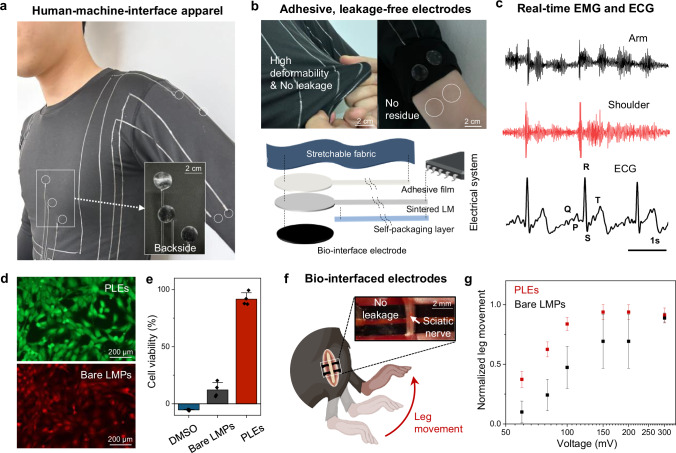
Large-scale integration of PEDOT:PSS/LB-LMPs electrodes (PLEs) and interconnects. **a** Photograph of the large-scale integration of LB-LMPs-based conductor on apparel. **b** Photograph and schematic illustrations of LB-LMPs-based biocompatible electrodes and interconnects. **c** Real-time mapping of EMG and ECG signals across various body parts using smart apparel. **d** LIVE/DEAD staining images of 3T3 fibroblast cells. Live cells in green, with dead cells in red. **e** Cell viability according to DMSO, bare LMPs-based electrodes and PLEs (*n* = 4 biologically independent samples). Values are presented as means ± SD. **f** Photograph and schematic illustration of LB-LMPs-based biocompatible electrodes implanted in the mouse sciatic nerve. **g** Stimulated leg movement versus stimulation voltage in PLEs versus bare LMPs-based electrodes at 50 Hz stimulation (*n* = 4 biologically independent samples). Values are presented as means ± SEM. Figure 5f was created in BioRender. Park, S. (2025) https://BioRender.com/21g8oop.

Eliminating the need for additional activation and packaging processes with LB-LMPs offers a scalable solution to overcome the traditional challenges faced by LMPs-based conductors. This innovation facilitates the direct and seamless integration of large-scale interconnects and surface-electrodes into commercialized apparel for bio-signal measurements (Fig. 5a and Supplementary Fig. 30). To streamline the integration of interconnects onto fabric, we adopt a one-step process utilizing LB-LMPs conductors applied onto a silicone adhesive film. In addition, a deformable and biocompatible PEDOT:PSS/LB-LMPs electrode (PLE) with high stability and biocompatibility were produced (Supplementary Figs. 31 and 32) by incorporating PEDOT:PSS into the polymer to create electrical connections between LMPs and the composite matrix (Supplementary Fig. 33). Integrating LB-LMPs interconnects and PLEs into fabrics leads to the creation of a smart apparel system, demonstrating a deformable electrical system without any leakage of liquid metal during or after use (Fig. 5b). This enables the real-time monitoring of electromyogram (EMG) and electrocardiogram (ECG) signals across various body parts using a LB-LMPs-based smart apparel (Fig. 5c).

Moreover, LMPs-based implantable devices have recently gained attention for their low modulus and high electrical conductivity. However, the risk of leakage significantly hinders their broader application, necessitating extra packaging or treatments. Our PLE, leveraging PEDOT:PSS and LB-LMPs, overcomes this issue by providing highly stable and biocompatible interfaces. In addition, compared to the bare LMPs-based electrode, the PLE interface improves cell viability due to the sealing of LMPs surfaces with a biocompatible PEDOT:PSS^58–60^ (Fig. 5d, e). In vivo biocompatibility was evaluated through dorsal subcutaneous implantation in mice for 2 weeks, revealing a minimal inflammatory response comparable to a biocompatible control, supporting their potential for long-term implantation^61^ (Supplementary Fig. 34). By combining the high charge injection capabilities of PEDOT:PSS^62,63^ with LMPs electronics via PLEs, we enabled the development of highly conductive, deformable, and leakage-free neural interfaces (Fig. 5f). In contrast, bare LMPs-based electrodes experienced a leakage of liquid metal, potentially compromising its durability. The PLE demonstrates the ability to stimulate the sciatic nerve in vivo with low voltages, up to 60 mV (Fig. 5g), as well as exhibits high reliability in electrically-stimulated leg movement over a broad range of stimulation frequencies (2 ~ 50 Hz)^63–65^ (Supplementary Fig. 35). The combination of LB-LMPs with various conductive materials through this process shows versatility and reliable performance as bioelectronic interfaces, highlighting the potential for effective liquid metal use in diverse bio-applications.

## Discussion

Conventional LMPs-based S-PCBs require an additional activation process, as well as a packaging process to prevent leakage and ensure electrical insulation. In our study, we demonstrate the use of ligand-assisted sintered LMPs to enable one-step circuit printing. Amine-based ligands, formed during the fabrication process, bind to the surface of LMPs, effectively suppressing oxidation by creating a barrier on the surface. The in situ sintering of LB-LMPs within the polymer matrix facilitates vertical phase separation, resulting in the automatic formation of a packaging layer. This process enables the large-scale production of stretchable circuits. Furthermore, the selective electrical conductivity of the packaging layer allows for the precise development of multi-layer circuits and biomedical devices. We envision that this method offers significant potential for a broad range of LMPs-based stretchable interconnectors and electrodes, thanks to its streamlined processing, scalability, and adaptability to various polymers.

## Methods

### Materials

Unless specified, all chemicals employed in the present study were utilized without additional purification. The procurement of all chemicals was conducted through Sigma-Aldrich, unless explicitly indicated otherwise. LM alloy (eutectic gallium indium, EGaIn, 99.99%) was purchased from Changsha Ruichi Nonferrous Metals, and Ag flakes (0.5 ~ 2 μm) were obtained from Daejoo Electronics Materials Co. Thermoplastic polyurethane (TPU, Elastollan® L1185A) was purchased from Goodfellow. Polycaprolactone (PCL, Mn 80,000), Poly(vinyl alcohol) (PVA, Mw 89,000–98,000), Poly(butylene adipate co-terephthalate) (PBAT), and Poly(3,4-ethylenedioxythiophene)-poly(styrenesulfonate) (PEDOT:PSS, dry re-dispersible pellets) were used as the polymer matrix of LMPs. Hydrogel (HydroMed™ D4) was fabricated by AdvanSource Biomaterials, and shape-memory polymer (SMP, MM3520) was acquired from SMP Technologies Inc. N-Methylpyrrolidone (NMP, 99%) and cyclohexane (99%) were used as solvents to disperse LMPs. Butylamine (99.5%) and butyric acid (99%) used as additional ligands. For printing the substrate, PDMS (Sylgard 184, Dow Corning), VHB tape (3 M), Kapton tape (3 M) and Tegaderm (3 M) were used. Water-Soluble Wave Solder Tape 5414 (3 M) was used as release tape.

### Preparation of LB-LMPs solution

2.4 g of LM was subjected to ultrasonication (VC-505, Sonics & Materials, 3 mm microtip) in a 6 mL of NMP solvent, utilizing a 40% amplitude for 20 min. The treatment of bare LMPs involved ultrasonication under conditions maintained by an ice bath.

### Preparation of LB-LMPs/polymer ink

The LB-LMPs solution was incorporated into a polymer solution (1.2 g of TPU in 18 mL of NMP), undergoing mixing for a duration of 5 min utilizing a Thinky mixer (AR-100). The volume of the polymer solution was adjusted to achieve LMPs volume fraction $({V}_{{LMPs}}/({V}_{{LMPs}}+{V}_{{polymer}}))$ of 25%, 50%, and 75%, enabling a comparison of resistance based on the volume fractions. In addition, Hydrophilic PU (D4), PCL, PVA, PBAT, and SMP were dissolved in NMP at the same concentration of 0.1 g/ml at identical concentrations and subsequently combined with the LB-LMPs solution at a consistent LMPs volume fraction of 25%. LB-LMPs/polymer films were prepared by evaporating the solvent from the LB-LMPs/polymer ink using a hotplate set at 60 °C. We conducted screen printing, nozzle printing, and dip coating with LB-LMPs/polymer ink by adjusting the viscosity of the ink. The viscosity of the ink was adjusted by varying the polymer concentration. For screen printing, the ink was formulated by dissolving 0.6 g of TPU in 12 ml of NMP to create the polymer solution. For dip coating, the polymer solution consisted of 0.6 g of TPU dissolved in 9 mL of NMP. For nozzle printing, the polymer solution was prepared by dissolving 0.6 g of TPU in 6 mL of NMP. In all cases, the LB-LMPs solution was prepared separately by performing tip sonication on 1.2 g of LM in 3 mL of NMP, followed by thorough mixing with the polymer solution using a Thinky mixer. For large area integration on adhesive film, we coated the LB-LMPs film on VHB tape and conducted laser cutting (3-Axis UV Laser Marker, Keyence).

### Peel test of LB-LMPs/polymer composites

In order to assess the extent of LM leakage, a peel test was conducted on the upper surface of the LMPs/TPU film using a Materials Testing Machine (LS1, Ametek). The film on a glass plate was securely positioned in a vertical orientation with a gripper. Following the affixation of a 10 mm-wide polyimide tape onto the surface of the film, the peel strength was determined by gradually peeling off the tape at a 180° angle, at a constant rate of 200 mm/min.

### Vertical electrical connection with top polymer layer

To establish vertical electrical connections with the top polymer layer, AgF or PEDOT:PSS ink was deposited onto the printed LB-LMPs/polymer ink. The AgF ink was prepared by adding 2 g of AgF to a TPU solution composed of 0.8 g of TPU dissolved in 12 mL of NMP. The PEDOT:PSS ink was prepared by dissolving 0.3 g of TPU and 0.15 g of PEDOT:PSS in 15 mL of NMP. AgF and PEDOT:PSS inks were fabricated through tip-sonication of the solution with the same solvent and polymer used for the LB-LMPs/TPU ink.

### Fabrication of stretchable display

After screen printing the LB-LMPs/TPU ink onto a VHB film, the electrically connected regions was defined by dropping AgF ink. For the second layer, a film was formed on PDMS and cut to a specific pattern with AgF composite. This second layer was then transferred onto the first layer, patterned on the VHB film. Following the transfer, the LED was attached to the AgF composite region.

### Fabrication of multi-layer S-PCBs

Following the screen printing of LB-LMPs/TPU ink onto a glass plate, the electrically connected regions for VIAs or conductive pads were defined by applying AgF ink. After evaporation at 60 °C, the printed circuits were soaked in water for 30 min and then easily removed using release tape. The printed circuit of each layer was sequentially transferred onto VHB tape. All chips were then fixed in place using silver paste and epoxy, applied in the order. A red LED was utilized to visualize circuit operation, while infrared emitters (APHD1608F3C-P22) were employed for PPG sensing. A temperature sensor (NCP18WF104F12RB) was also integrated into the circuit.

### Fabrication of PEDOT:PSS/LB-LMPs electrode (PLE)

The fabrication process begins by depositing the LB-LMPs/TPU ink onto the substrate on 60 °C hot plate. Subsequently, the PEDOT:PSS/TPU ink is precisely printed on top of the initial layer before the complete evaporation of NMP. The ink is then dried at 60 °C and laser-cut into the desired design.

### Fabrication of human-interface apparel

To fabricate large-scale interconnector threads, LB-LMPs/TPU ink was poured onto VHB tape, followed by drying at 60 °C. Subsequently, the film was precisely cut to a width of 2 mm using a laser cutter. The electrical terminations at both ends of the interconnector threads were established using AgF ink. One end was knotted to a jumper cable and linked to an EP signal processing unit, while the other end was connected to PLEs with silver epoxy (CW2400, Chemtronics). For the garment assembly, 11 PLEs were incorporated for EMG and ECG measurements. The placement of these electrodes is strategically arranged on the inner surface of the garment, ensuring full contact with the human body. ECG and EMG signals were measured using a commercial electrophysiological signal monitoring system (BioRadio, Great Lakes NeuroTech). All experiments involving a human participant were conducted with informed consent.

#### Characterization

##### Morphological characterization

For the analysis of the surface morphologies of the LMPs, TEM images were acquired utilizing a transmission electron microscope (Tecnai F20, FEI company). The LMPs solution was diluted at a volume ratio of 100:1 with dichloromethane and subsequently deposited onto 400-mesh copper grids. In order to investigate the thickness of the oxide layer, TEM analysis was conducted within a timeframe of 1 h following the preparation of the samples. SEM images of LMPs and LMPs/TPU films were captured employing a Magellan 400 scanning electron microscope (FEI company). Prior to imaging, NMP was evaporated overnight at 60 °C on a silicon wafer. To obtain the bottom surface images of the LMPs/TPU films, the films underwent immersion in water for 10 min, and were then peeled off without tension. For cross-sectional imaging, the films were sectioned while submerged in liquid nitrogen.

##### Chemical characterization

To identify the ring opening of NMP, FTIR was analyzed utilizing an FTIR Spectrometer (Nicolet iS50, Thermo Fisher Scientific Instrument). The LMPs solution underwent centrifugation to isolate the LMPs, and only the supernatant was extracted. ^1^H NMR analysis was carried out using a liquid-state 600 MHz nuclear magnetic resonance spectrometer (Avance Neo 600, Bruker BioSpin) with chloroform-d (99.8 atom % D, contains 1 % (v/v) TMS, Sigma-Aldrich) utilized as a solvent. XPS analysis was performed using an In-Situ X-ray photoelectron spectroscopy (Nexsa G2, Thermo Scientific) to obtain Ga *3 d* and C *1 s* spectrum.

##### Electrical characterization

The electrical properties of the conductor were measured using an LCR meter (4284 A, HP) and a source meter unit (2450, Keithley). To measure the resistance of the backside of the LMPs/polymer film, the film was detached through water immersion, allowing for separation without applying mechanical force. To monitor resistance variation under strain, a special setup including a force gauge (with a maximum force of 50 N, Mark-10), a stand with a motor (Mark-10), and a customized manual strain machine were utilized.

##### Rheological characterization

The rheological properties of the LMPs/TPU ink were investigated utilizing a rheometer (MCR 302e, Anton Paar) equipped with a 25 mm diameter measuring plate. The determination of the apparent viscosity involved conducting steady-state flow experiments with a logarithmic variation of shear rate (0.01 to 100 s^−1^) at a temperature of 25 °C.

##### Cell viability test

Samples were prepared by depositing a singular droplet of ink onto Tegaderm tape, subjected to controlled evaporation at a temperature of 60°C overnight, followed by cutting off the edges. To investigate the biocompatibility of the samples, NIH/3T3 fibroblast cells (ATCC, US) were used. NIH/3T3 fibroblast cells were seeded in full Dulbecco’s modified Eagle’s medium (Gibco,US) supplemented with 10% fetal bovine serum (Gibco,US) and 1% penicillin/streptomycin (Gibco,US) in a 48-well plate with the seeding density of cells 5 × 10^4^/mL. After 24 h of incubation, the complete medium was discarded and refilled with fresh full medium. Next, Bare LMPs-based electrodes and PLEs were placed to the 48-well plates for evaluating the cytotoxicity of the samples. After 24 h of incubation, Live/Dead viability test (ThermoFisher, US) and CCK8 assays (ABCAM, US) were used to both quantify and qualify the cytotoxicity of the test samples. For Live/Dead fluorescence staining, images were captured using a fluorescent microscope (Nikon Ti2, Japan). As for the CCK8 assays, the absorbance at 460 nm was measured with a microplate reader (Molecular Devices, US).

##### Animal experiments and surgical procedure

Mice were housed and maintained under conditions of 12 h light/dark cycle and 22–24 °C, and given ad libitum access to food and water. Anesthetized was induced with 4 ~ 5% isoflurane under O2 flow and maintained by reducing isoflurane concentration to 1 ~ 2.5 isoflurane. Mice were sacrificed in CO2 gas.

##### In vivo biocompatibility of PLE

All animal surgeries were reviewed and approved by the Institutional Animal Care and Use Committee at Seoul National University Hospital (Approval No. SNU-250115-2). 8-weeks old male C57BL/6 N mice (Koatech) were used for all in vivo studies. PLE films were cut into 5 mm × 5 mm and attached to Tegaderm for easy handling. Tegaderm was cut into 5 mm X 5 mm for implantation as control group. The films were prepared using sterile techniques and further sterilized by immersing 100% Ethanol for 1 h and further sterilized under UV light for 30 min. The films were implanted in the dorsal subcutaneous pocket of the animal. Back hair was removed, and skin was sterilized with betadine. A 1-cm Incision was made carefully with surgical scissors. The films were inserted into the subcutaneous pocket, and the skin incisions were closed with sutures (5-0 Black silk, Ethicon), followed by 0.9% saline washing. After 2 weeks of implantation, the animals were euthanized by CO2 inhalation, and the skin under the film was dissected for histological analysis. After 2 weeks of implantation, the animals were euthanized by CO2 inhalation, and the skin under the film was dissected for histological analysis. The tissues were then fixed in 10% formalin for 24 h before paraffin-embedding. The paraffin-embedded fixed samples were sliced and prepared into slides. The slides were deparaffinized and underwent hematoxylin and eosin staining for histological analysis.

##### Sciatic nerve device implantation and electrical stimulation

All experiments were approved by the KAIST Institutional Animal Care and Use Committee (Approval No. KA2023-036-v2). C57BL/6 N mice (Koatech) aged 6 weeks or older were used during in vivo testing. After shaving the lower limb hair, the bicep femoris and gluteus maximus muscles were separated via incision. Forceps were used to gently detach the sciatic nerve from surrounding tissue. The PLE was then placed beneath the sciatic nerve before electrical stimulation. Biphasic electrical stimulation was delivered to the sciatic nerve with an isolated pulse stimulator (A-M Systems Model 2100). Stimulation trains were applied at 1 s train burst width with 5 ms pulse durations at various voltages and inter-pulse periods. A protractor was placed beneath the leg to measure the angle of leg movement during stimulation.

### Reporting summary

Further information on research design is available in the Nature Portfolio Reporting Summary linked to this article.

## Supplementary information

Supplementary Information

Reporting Summary

Transparent Peer Review file

## Source data

Source Data

## Data Availability

The authors declare that the data supporting the findings of this study are available within the article and its Supplementary Information files. Extra data supporting the findings of this study are available from the corresponding authors upon request. Source data are provided in this paper.
