# Supplementary material for: Self-packaged stretchable printed circuits with ligand-bound liquid metal particles in elastomer
Source: Nat Commun. 2025 May 28;16:4944. doi: 10.1038/s41467-025-60118-4 (PMC12119845; doi:10.1038/s41467-025-60118-4)
Supplement: Supplementary file 1 — Supplementary Information [file 41467_2025_60118_MOESM1_ESM.pdf]

Supplementary Information

Self-packaged stretchable printed circuits with ligand-bound liquid metal particles in elastomer

Hyeonyeob Seo[1,†], Gun-Hee Lee[1,2,3†], Jiwoo Park[4], Dong-Yeong Kim[1], Yeonzu Son[5], Semin Kim[6], Kum Seok Nam[1], Congqi Yang[7], Joonhee Won[8], Jae-Young Bae[9], Hyunjun Kim[8], Seung-Kyun Kang[9], Steve Park[8,10], Jiheong Kang[11,*], and Seongjun Park[6,12,13,14,15,*]

[1] Department of Bio and Brain Engineering, Korea Advanced Institute of Science and Technology (KAIST), 291 Daehak-ro, Yuseong-gu, Daejeon 34141, Republic of Korea.

[2] Departments of Cogno-Mechatronics Engineering, Pusan National University, Busan 46241, Republic of Korea.

[3] Departments of Optics and Mechatronics Engineering, Pusan National University, Busan 46241, Republic of Korea.

[4] School of Electrical Engineering, Korea Advanced Institute of Science and Technology (KAIST), 291 Daehak-ro, Yuseong-gu, Daejeon 34141, Republic of Korea.

[5] Program of Brain and Cognitive Engineering, Korea Advanced Institute of Science and Technology (KAIST), Daejeon, 34141, South Korea.

[6] Graduate School of Semiconductor Technology, Korea Advanced Institute of Science and Technology (KAIST), 291 Daehak-ro, Yuseong-gu, Daejeon 34141, Republic of Korea.

[7] Medical Research Center, Seoul National University, Seoul 03080, Republic of Korea.

[8] Department of Materials Science and Engineering, Korea Advanced Institute of Science and Technology (KAIST), 291 Daehak-ro, Yuseong-gu, Daejeon 34141, Republic of Korea.

[9] Department of Materials Science and Engineering, Seoul National University, 1 Gwanak-ro, Gwanak-gu, Seoul 08826, Republic of Korea.

[10] KAIST Institute for NanoCentury, 291 Daehak-ro, Yuseong-gu, Daejeon 34141, Republic of Korea.

[11] Department of Chemistry, Seoul National University, Seoul 03080, Republic of Korea.

[12] Department of Biomedical Sciences, College of Medicine, Seoul National University, Seoul 03080, Republic of Korea.

[13] School of Transdisciplinary Innovations, Seoul National University, Seoul 03080, Republic of Korea.

[14] Interdisciplinary Program in Bioengineering, College of Engineering, Seoul National University, Seoul 08826, Republic of Korea.

[15] Department of Transdisciplinary Medicine, Seoul National University Hospital.

†These authors contributed equally to this work.

*Corresponding authors: J. K. (jiheongkang@snu.ac.kr), S. P. (seongjunpark@snu.ac.kr)

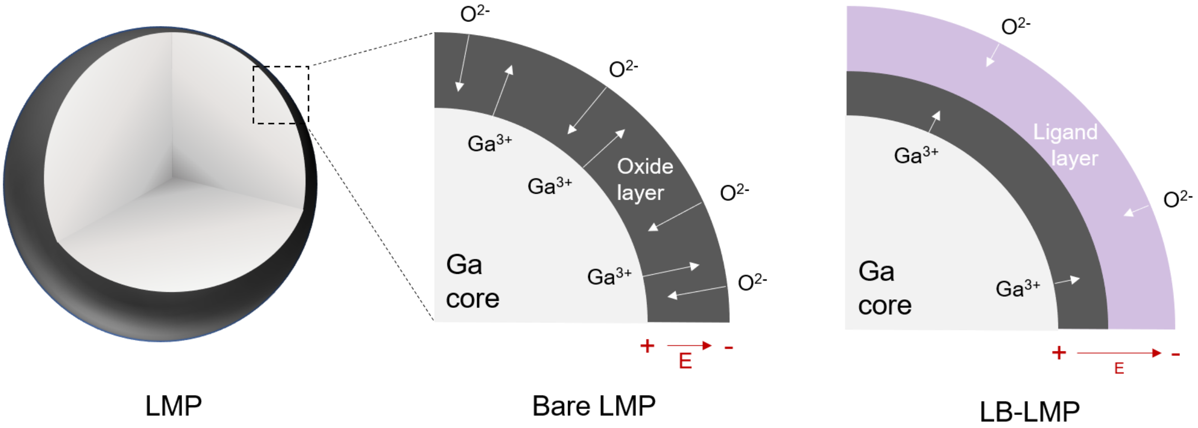

Supplementary Fig. 1| Schematic illustration of the Cabrera-Mott mechanism.

According to the Cabrera-Mott theory, oxygen chemisorption on the surface of LMP results in the formation of gallium oxide. Electrons within the gallium core traverse the oxide layer, inducing the ionization of oxygen[1,2]. The interplay between gallium cations within the core and oxygen anions on the surface generates an electric field that facilitates oxide formation. As the oxide layer thickens, the electric field diminishes, leading to the cessation of oxide formation[3,4]. In the presence of a surface-bound ligand, LMP not only impedes the transfer of electrons from the gallium core to oxygen but also attenuates the strength of the electric field by spatially separating gallium cations and oxygen anions. Consequently, ligand hinders oxide formation on the LMP surface.

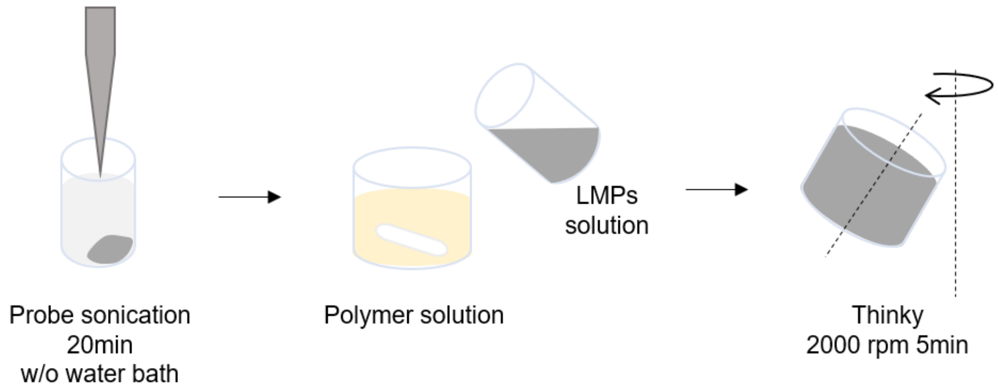

Supplementary Fig. 2| Fabrication of LB-LMPs/polymer ink.

Bulk LM was immersed in NMP, subjected to ultrasonication, and subsequently mixed through rotational agitation with the polymer solution.

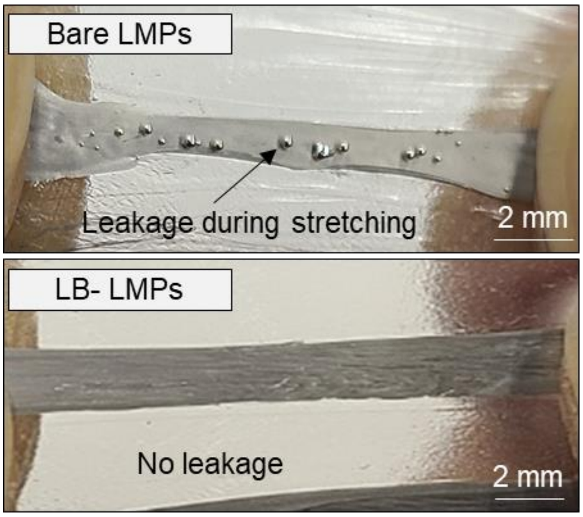

Supplementary Fig. 3| Bare LMPs or LB-LMPs/polymer printed on stretchable polymer substrate.

Bare LMPs/polymer composites exhibit LM leakage during stretching and display susceptibility to fracture even under weak tension. In contrast, LB-LMPs/polymer composites demonstrate a self-packaging, mitigating the issue of LM leakage, and displaying enhanced mechanical resilience.

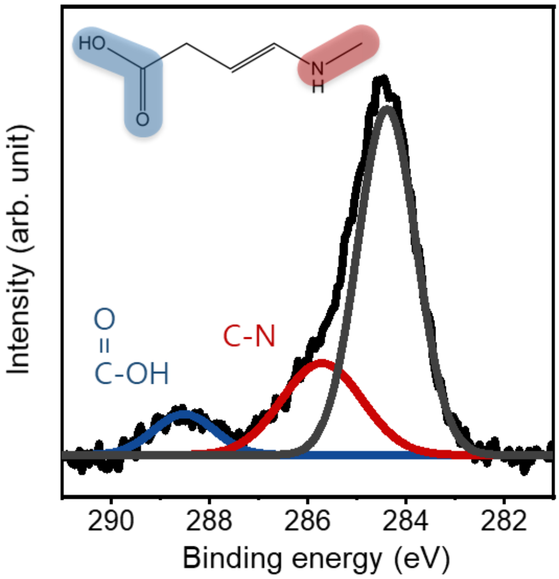

Supplementary Fig. 4| XPS spectrum of C 1*s* on the top surface of LB-LMPs.

In XPS spectrum on the top surface of LB-LMPs, the presence of a C-N peak (~285.8 eV) and a carboxyl bond peak (~288.6 eV) was observed.

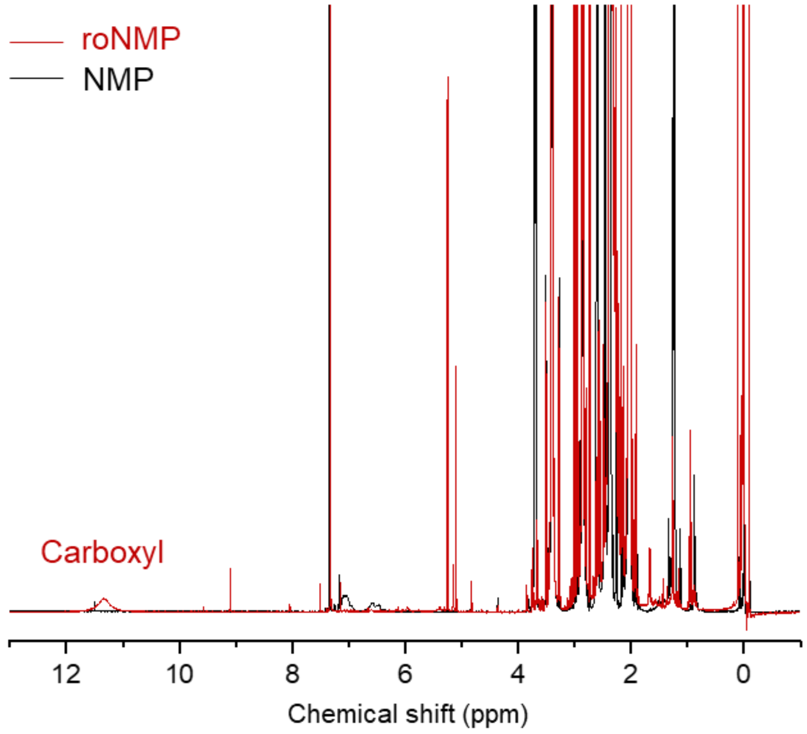

Supplementary Fig. 5| ^{1}H NMR spectrum of pure NMP and roNMP.

Proton NMR analysis was conducted on both pure NMP and roNMP. roNMP was derived by selectively removing LMPs subsequent to 20 minutes of ultrasonication. The spectra were recorded in $CDCl_3$ at 600 MHz.

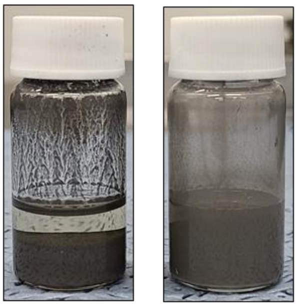

Supplementary Fig. 6| Bare LMPs and LB-LMPs dispersed in NMP after 6 hours.

Bare LMPs (left) exhibit a propensity to readily sink in NMP, whereas LB-LMPs (right) demonstrate enhanced dispersion characteristics.

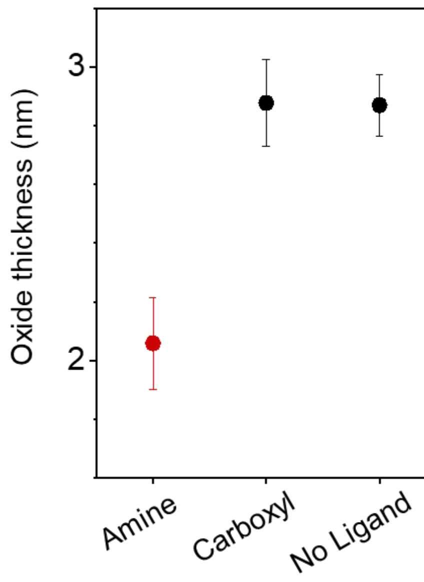

Supplementary Fig. 7| Oxide layer thickness of LMPs depending on the functional group of the ligand (n=12, 4 biological replicates). Values are presented as means ± SD.

Each ligand bound LMP as synthesized by sonication in cyclohexane in the presence of the ligand. The ligand featuring an amine group reduce the oxide layer thickness of LMPs.

According to HSAB theory, carboxyl groups, being harder bases, have a higher affinity for the hard acid Ga^{3+} ions compared to softer bases like amine groups[5,6]. When carboxyl groups attach to the Ga^{3+} ions on the LMP surface, they can enhance the outward diffusion of Ga^{3+} ions. This outward diffusion promotes further oxide layer formation[2]. In contrast, the softer base, the amine group, is less likely to facilitate the outward diffusion of Ga^{3+} ions, thereby preventing further oxide layer formation. This distinction highlights the role of chemical interactions at the molecular level in determining the behavior of oxide layer formation on LMP surfaces.

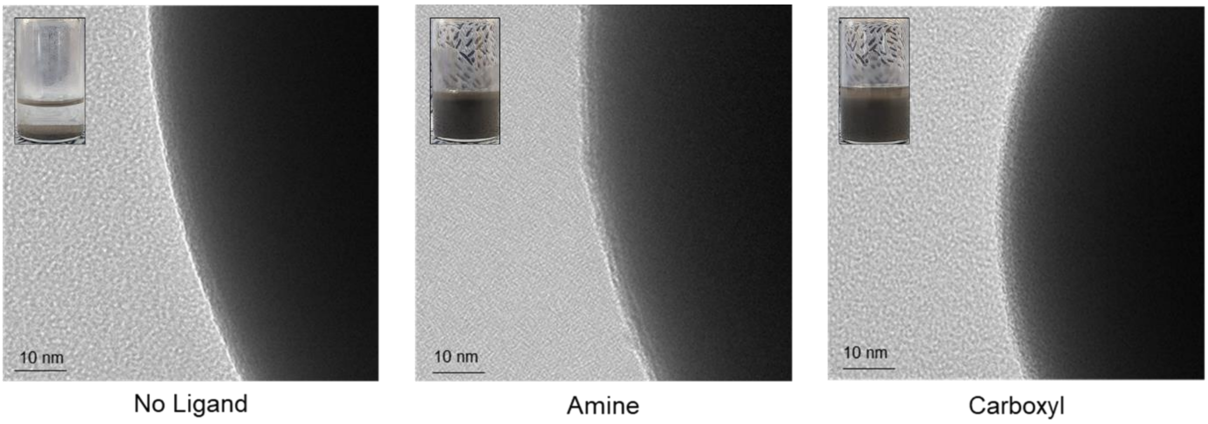

Supplementary Fig. 8| TEM images of LMP surface depending to the functional groups of the ligand (4 biological replicates).

TEM images capturing the surface morphology of LMP subsequent to ultrasonication in cyclohexane, while incorporating ligands, were obtained. Additionally, the solution containing the ligand exhibited significantly enhanced dispersion compared to the solution without the ligand.

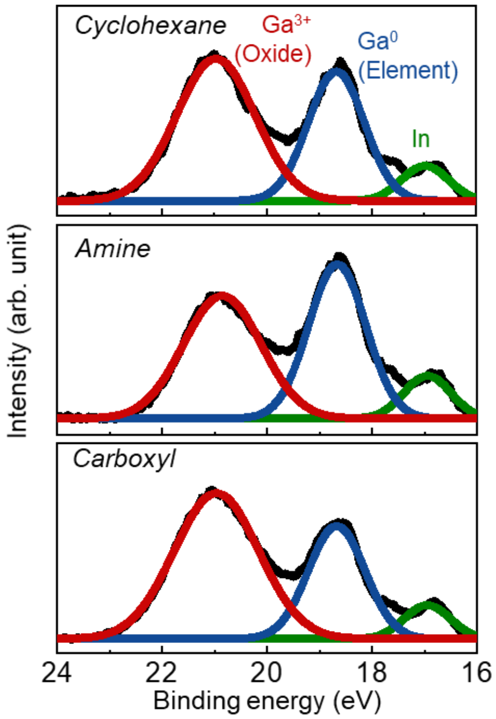

Supplementary Fig. 9| XPS of Ga 3*d* of LMPs depending to the functional groups of the ligand.

The red curve (~21 eV) corresponds the Ga^{3+} peak, indicative of the presence of gallium oxide, while the blue curve (18-19 eV) corresponds to the Ga^{0} peak.

.

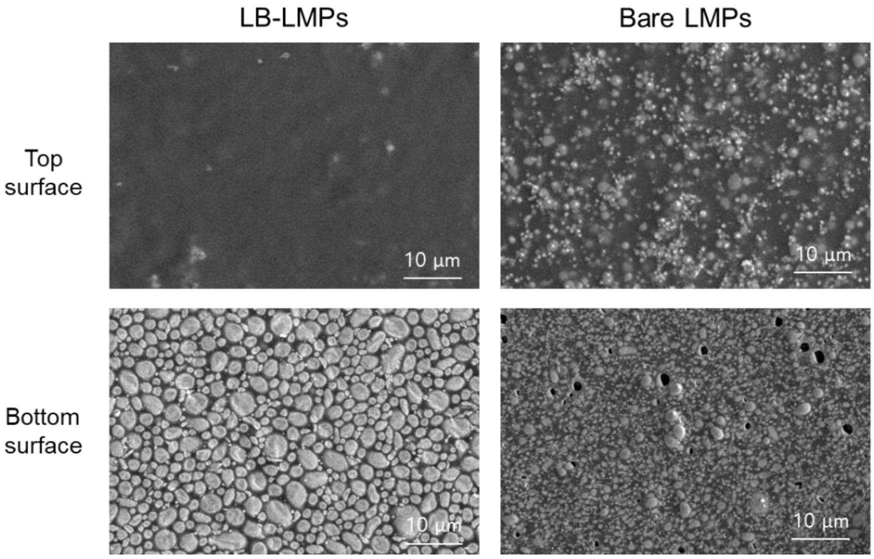

Supplementary Fig. 10| SEM images on the top and bottom surface of the LB-LMPs/polymer and bare LMPs/polymer films (3 biological replicates).

Vertical phase separation was observed in the LB-LMPs/polymer film, leading to the sintering of LMPs on the bottom surface and packaging of polymer on the top surface. Conversely, in the bare LMPs/polymer film, LMPs were found to be uniformly distributed across both the top and bottom surfaces.

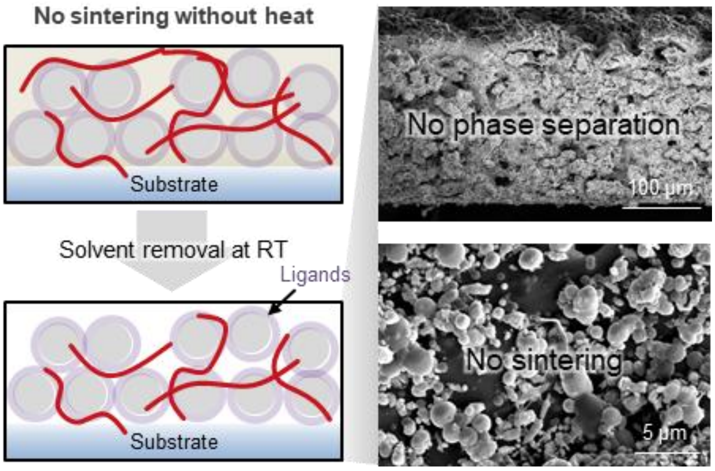

Supplementary Fig. 11| Schematic illustration and cross-sectional SEM images without ligand desorption (3 biological replicates).

When LB-LMPs/polymer films are manufactured at ambient temperature without heat, ligand detachment does not occur. In films where ligand separation does not occur, LMPs sintering and phase separation is inhibited.

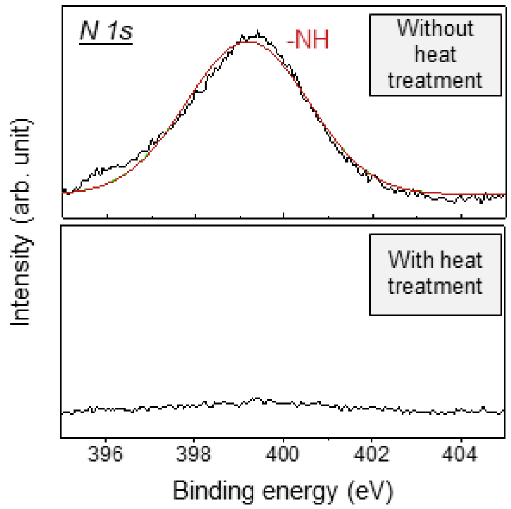

Supplementary Fig. 12| XPS of N 1*s* of LB-LMPs/PS film with/without heat treatment

To investigate ligand detachment, we conducted XPS analysis on LB-LMPs/PS films using polystyrene (PS), a carbon-hydrogen polymer. XPS measurements were performed on the bottom layer of two film types: those prepared by heat-induced solvent evaporation and those by solvent separation at room temperature. In the N 1s spectrum, a peak at 399.5 eV appeared only in the latter, indicating incomplete desorption of amine-based ligands without heat treatment, leaving residual ligands on the LMP surface.

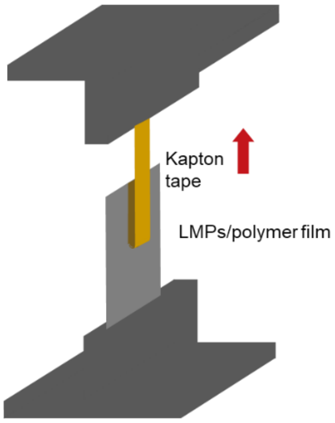

Supplementary Fig. 13| Schematic illustration of peel strength test.

Kapton tape was affixed onto the surface of the LM/TPU film, and the force required for its removal was quantified by peeling it off at a 180°. Due to the high surface tension of the LM, its adhesion to tape is poor, resulting in a weakened peeling force exerted on the film due to LM leakage on the surface.

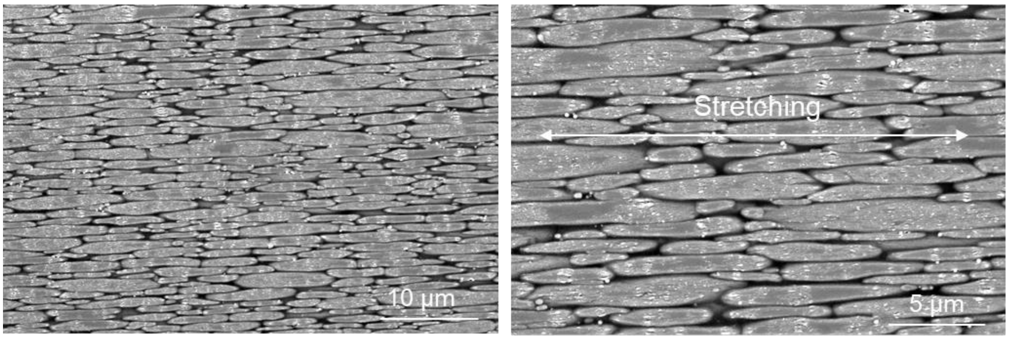

Supplementary Fig. 14| SEM images on the bottom surface of the stretched LB-LMPs/polymer films (3 biological replicates).

As LMPs elongate during stretching, their length increases; however, due to the simultaneous increase in contact area between the particles, there is only minimal change observed in resistance[7].

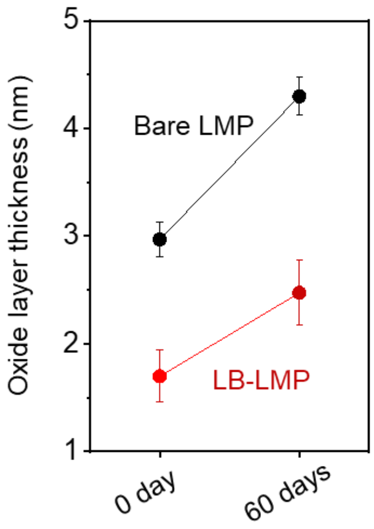

Supplementary Fig. 15| Oxide layer thickness of LB-LMPs and bare LMPs after 0 and 60 days (n=12, 4 biological replicates). Values are presented as means ± SD.

The thickness of the oxide layer on the surface of the LMPs immersed in NMP was measured after 0 and 60 days. Error bars indicate standard deviation.

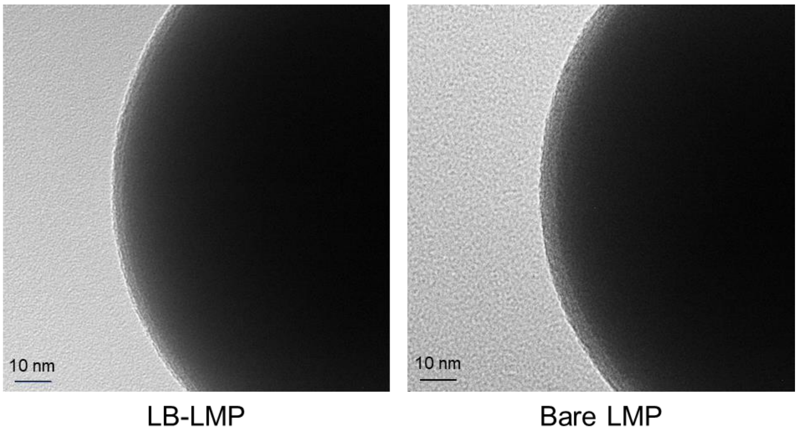

Supplementary Fig. 16| TEM images of LB-LMP and bare LMP after 60 days (4 biological replicates).

The morphologies of the oxide layer on the surface of LMP immersed in NMP was measured after 0 and 60 days.

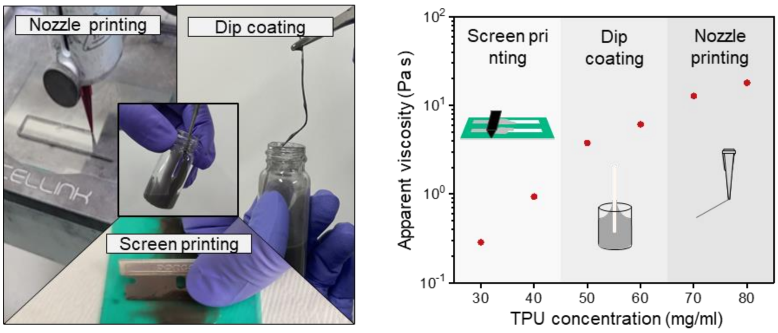

Supplementary Fig. 17| Design of LB-LMPs/polymer ink for various solution processes.

The utilization of LB-LMPs/polymer ink extends to multiple fabrication techniques such as screen printing, dip coating, and nozzle printing. This adaptability is achieved through viscosity modulation, accomplished by altering the polymer concentration within the ink formulation.

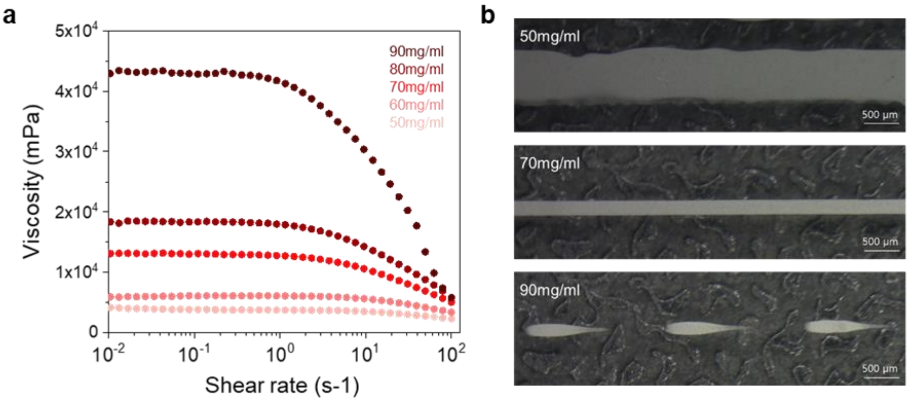

Supplementary Fig. 18| Rheological behavior and printing performance of LB-LMPs/TPU Inks. a, Shear-rate-dependent viscosity of LB-LMPs/TPU inks. **b,** Printed circuit lines using nozzle-based printing at varying ink concentrations.

LB-LMPs/TPU inks exhibited shear-thinning behavior across TPU/NMP concentrations ranging from 50 mg/mL to 90 mg/mL, confirming their suitability for nozzle-based printing.

Nozzle-based printing was conducted at a driving pressure of 80–120 kPa and a printing speed of 1600 mm/min using a 27G stainless steel jetting tip. The ink with a concentration of 50 mg/mL spread to approximately three times the nozzle diameter, whereas the 90 mg/mL ink led to discontinuous printing. In contrast, the 70 mg/mL ink enabled stable printing, achieving a circuit resolution of 200 μm.

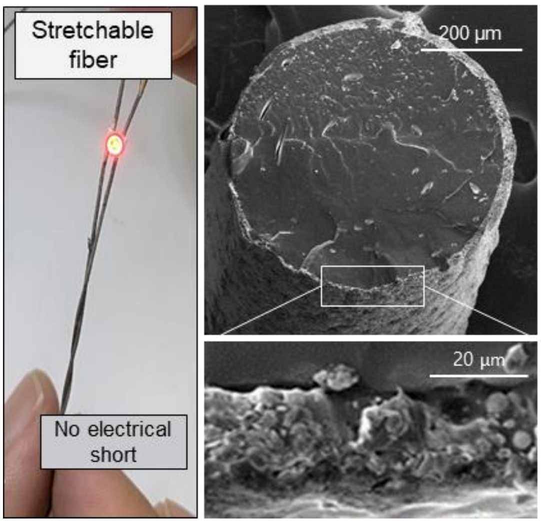

Supplementary Fig. 19| Conductive fiber coated with LB-LMPs/polymer ink (3 biological replicates).

The production process involved the dip coating of SEBS fiber with LB-LMPs/polymer ink to create conductive fibers. This procedure resulted in the sintering of LMPs on the fiber's surface, while polymer packaging occurred externally. Consequently, a conductive pathway was established while concurrently mitigating the risk of electrical short-circuiting.

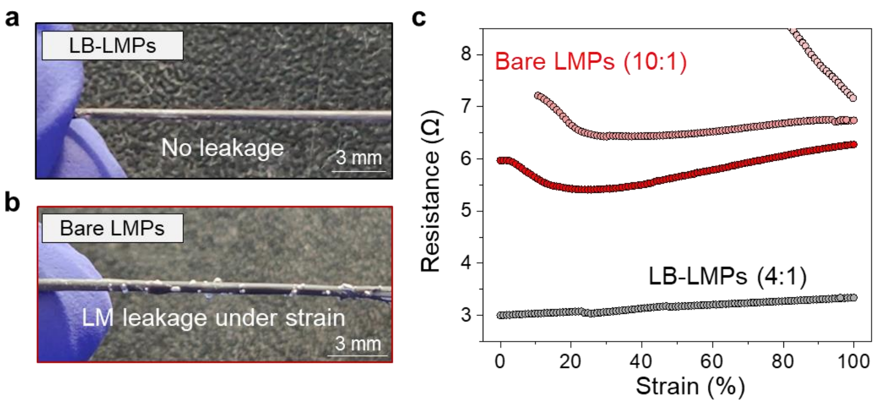

Supplementary Fig. 20| LB-LMPs-coated stretchable conductive fiber. a, Photograph of LB-LMPs-coated fiber under stretching. **b**, Photograph of bare LMP-coated fiber under stretching. **c,** Resistance of LB-LMPs-coated fiber and bare LMPs-coated fiber under strain.

LB-LMPs are easily coated on fiber with high viscosity ink. Resistance of LB-LMPs fiber show the stable under strain without any leakage of LM. On the other hand, bare LMPs-coated fiber shows the initial decrease of resistance with the leakage.

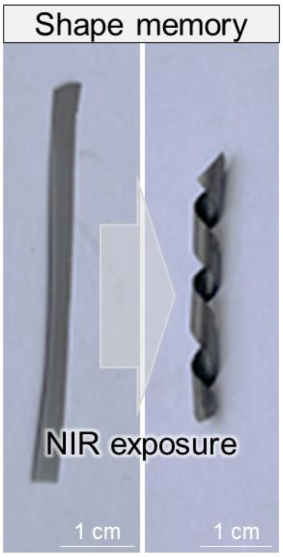

Supplementary Fig. 21| LB-LMPs with shape memory polymer (SMP).

The LB-LMPs/SMP film demonstrated the retention of its shape memory characteristic pertaining to coiling following near-infrared (NIR) irradiation.

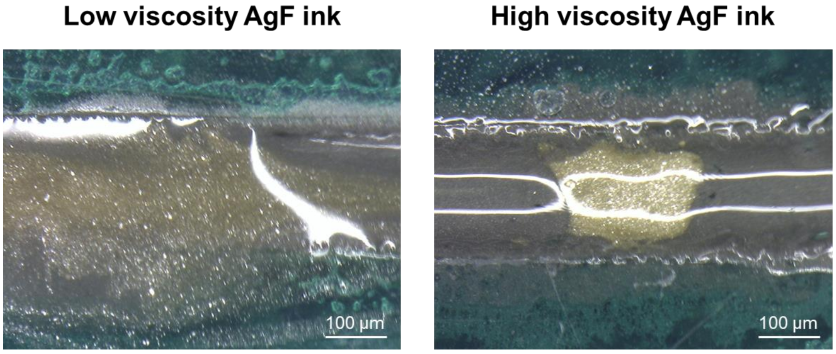

Supplementary Fig. 22| Optical images of printed results on screen-printed LB-LMP/Polymer ink layers at different AgF ink viscosities.

Low-viscosity AgF ink spreads uncontrollably, mixing with the base layer, while high-viscosity AgF ink maintains precise deposition.

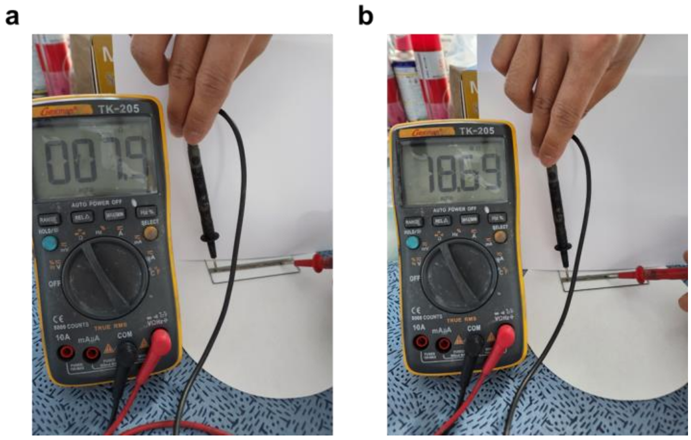

Supplementary Fig. 23| AgF-based selective electrical connection. a, Resistance between AgF connection (7.9 Ohm). **b,** Resistance between AgF and a packaged layer (18.69 Ohm).

The AgF enables electrical connectivity through the top insulated film. Unlike LM, the solid AgF provides both electrical conductivity and mechanical stability.

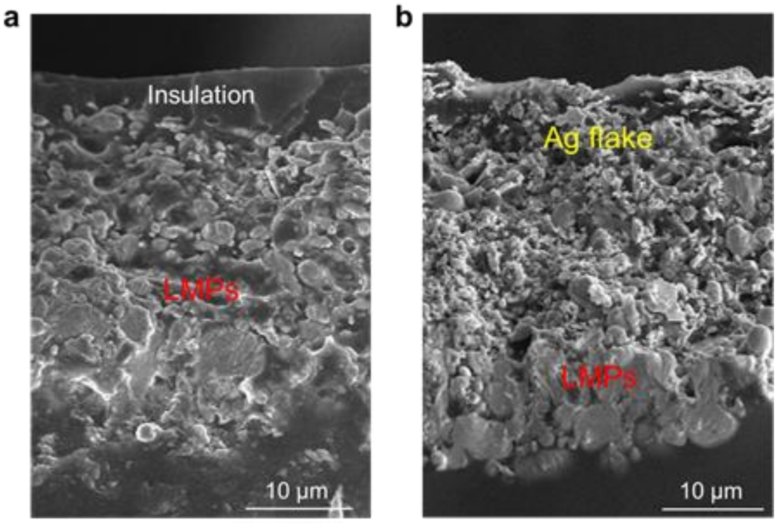

Supplementary Fig. 24| Stable AgF electrodes for robust integration. a, Photogarph of AgF electrodes**. b,** Photograph of LED integrated on AgF electrodes (3 biological replicates).

Solid AgF electrodes, unlike fluidic LM, enable the robust integration of conventional electronic components without any leakage.

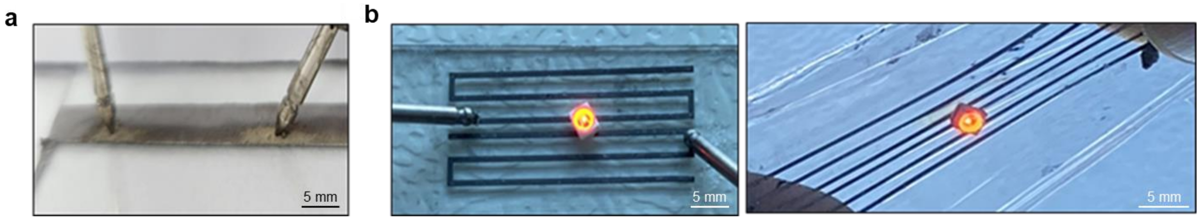

Supplementary Fig. 25| Stable AgF electrodes for robust integration. a, Photogarph of AgF electrodes**. b,** Photograph of LED integrated on AgF electrodes.

Solid AgF electrodes, unlike fluidic LM, enable the robust integration of conventional electronic components without any leakage.

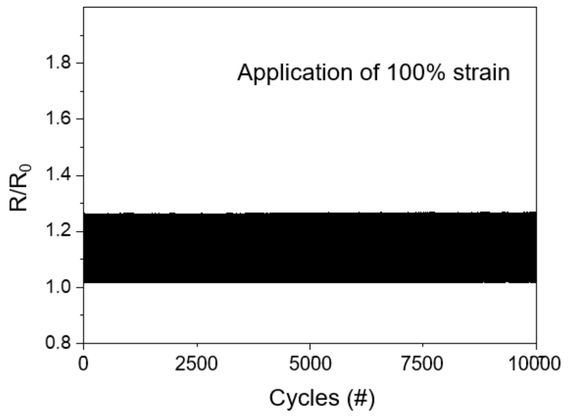

Supplementary Fig. 26| Relative resistance of LB-LMPs under repeated stretching cycles.

A LM-LMP/Polymer conductor retain its initial resistance even after 10,000 cycles at 100% strain.

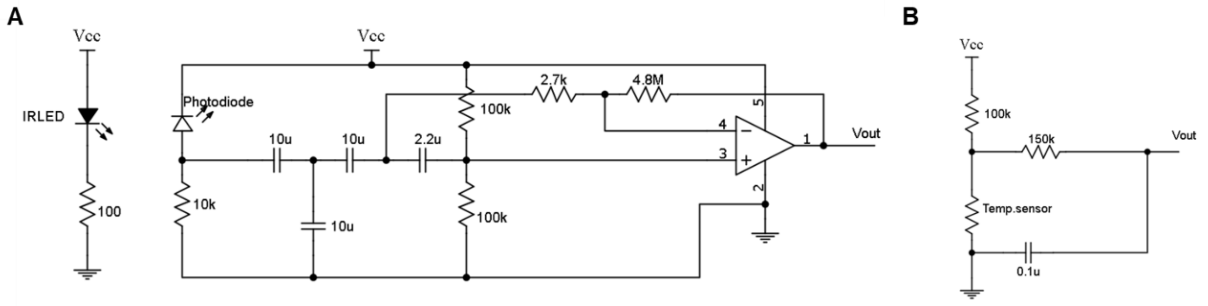

Supplementary Fig. 27| Circuit diagram of the PPG and temperature sensor circuit board. (A) The PPG sensor circuit. **(B)** The temperature sensor circuit.

The healthcare monitoring system consists of two circuits: one for the PPG sensor and another for the temperature sensor. In the PPG sensor circuit, infrared light (940 nm, Kingbright) emitted from an infrared source is absorbed by a photodiode (940 nm, Everlight). A capacitor coupled with the photodiode filters out the DC component, and the capacitor network functions as a low-pass filter with a 20 Hz cutoff frequency to remove high-frequency noise. The resulting AC PPG signal is then amplified by a factor of 1700 using an inverting amplifier (R5, R6, op-amp). Two large resistors (R3, R4, 100 kΩ) set the DC level to half of VCC to prevent output signal swing limitations.

For the temperature sensor circuit, an NTC thermistor is employed as the temperature-sensitive element. The thermistor's resistance decreases as temperature increases. The voltage across the thermistor is determined by a voltage divider formed by the thermistor and a 100 kΩ resistor. A filter is included to reduce noise in the output signal, ensuring cleaner and more accurate temperature measurements.

Both the processed PPG and temperature signals are recorded using an Arduino Uno R3, which reads the output signals through its analog input pins.

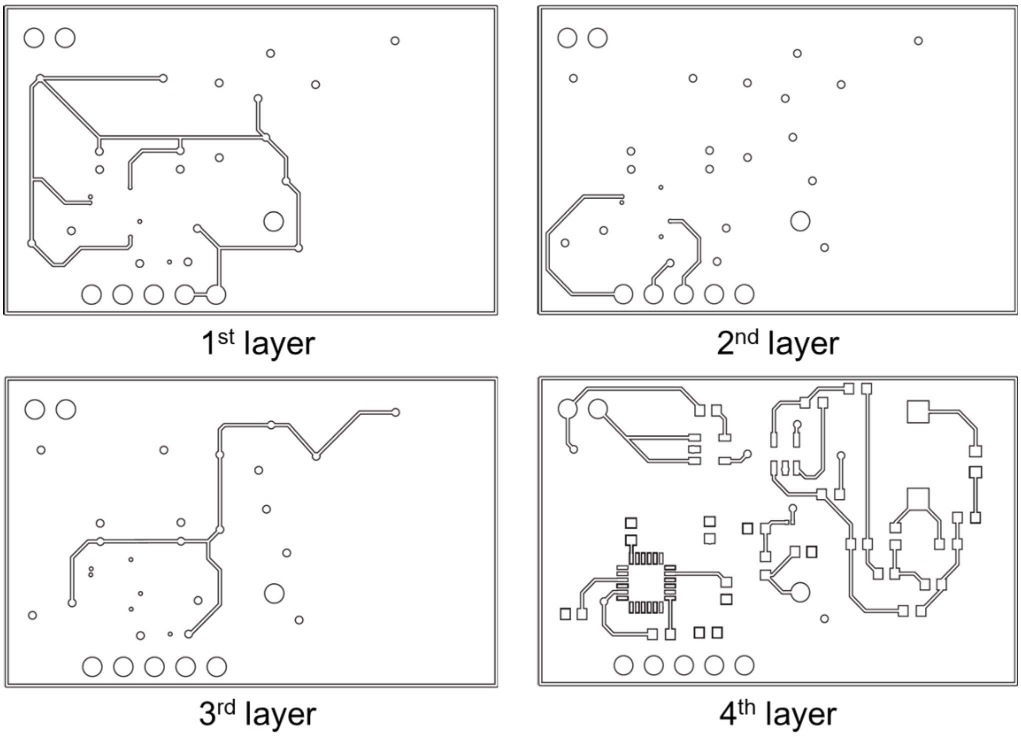

Supplementary Fig. 28| Schematic Diagram of a 4-Layer Integrated S-PCB.

LB-LMPs/polymer-based circuits were sequentially laminated and integrated onto VHB tape. Electrical conductivity in the VIA of the circuit was selectively achieved using AgF.

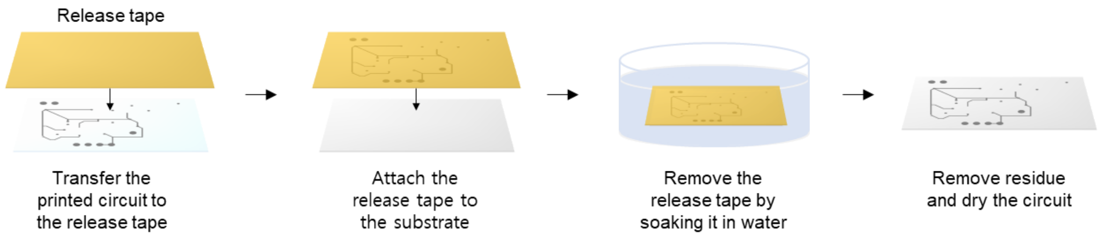

Supplementary Fig. 29| Transfer Process for S-PCB Fabrication.

Printed circuits were immersed in water, allowing for easy removal using release tape. Each printed circuit layer was then sequentially transferred onto the target substrate, enabling the assembly of multi-layer S-PCBs.

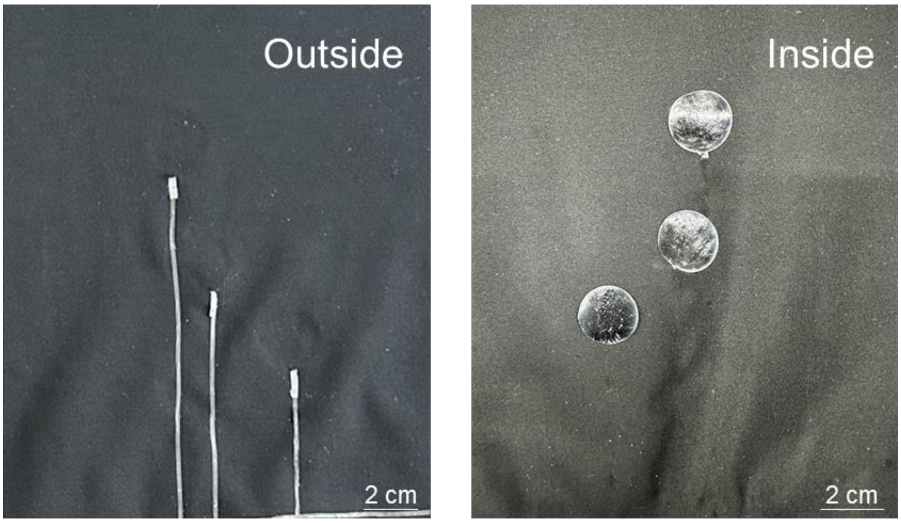

Supplementary Fig. 30| Integration of PEDOT:PSS/LB-LMPs electrodes (PLEs) and interconnector in the electronic suit.

The LB-LMPs/TPU threads designated for the interconnector and the PLEs utilized for ECG and EMG sensing were affixed to distinct sides.

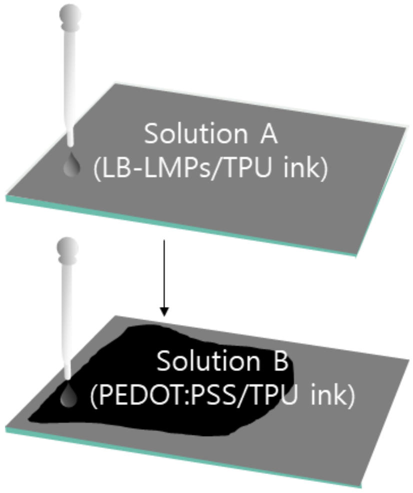

Supplementary Fig. 31| Schematic illustration of electrical connection on top layer.

For electrical connection on top layer, PEDOT:PSS/TPU ink was dropped onto the LB-LMPs/TPU ink prior to full evaporation of ink.

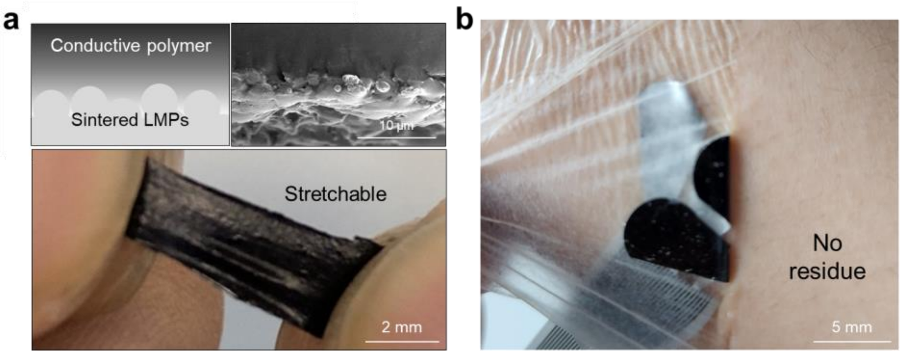

Supplementary Fig. 32| Bilayer coating for a PLE. a, Photograph of the PLE with cross-sectional SEM image. **b**, Photograph of the PLE on skin. There are no leakage or residue during usage.

Vertical phase separation between the conductive polymer (PEDOT:PSS) and LB-LMPs is achieved through the examination of SEM images.

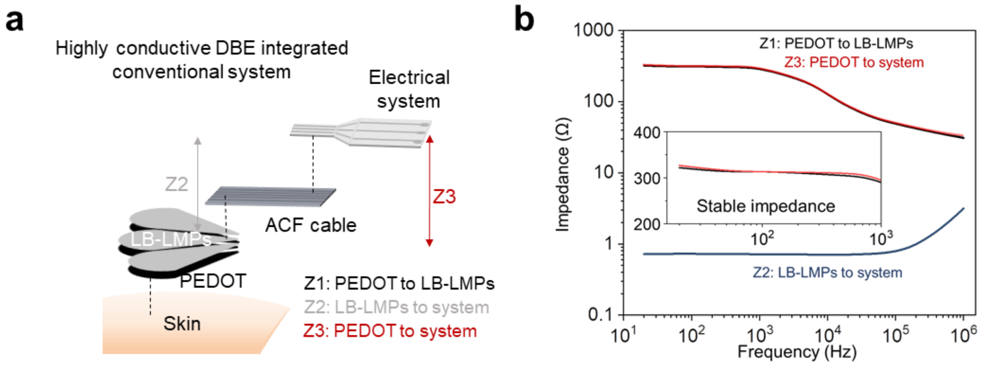

Supplementary Fig. 33| Electrical property of a PLE. a, Schematic illustration of the PLE integrated conventional system. **b,** Impedance between each component of the PLE.

PLE shows high electrical conductivity within the interconnect area of LMPs. Only the junction between LMPs and PEDOT:PSS demonstrates non-metallic conductivity.

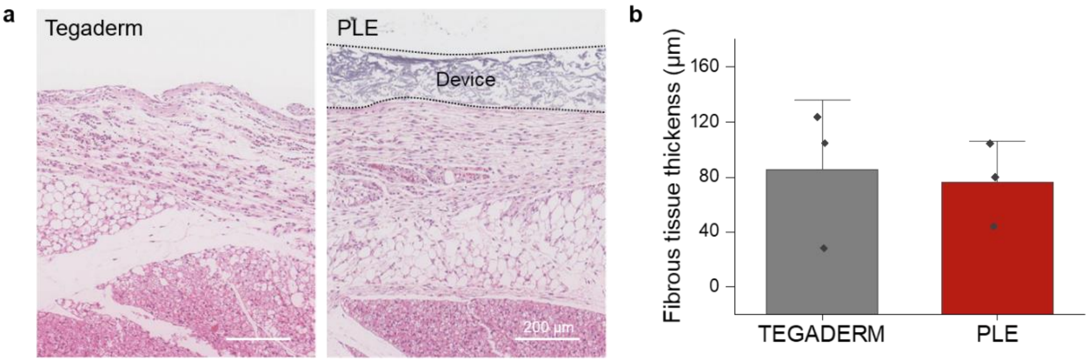

Supplementary Fig. 34| Long-term in vivo biocompatibility of PLE. a, Histological evaluation of subcutaneous implantation of PLE and Tegaderm. **b,** Quantification of fibrotic response of PLE and Tegaderm (n=3 biologically independent samples). Values are presented as means ± SD.

The in vivo biocompatibility of PLEs was evaluated through subcutaneous implantation in mice, followed by H&E staining at 2 weeks post-implantation. The fibrotic tissue thickness around PLEs (75.92 ± 30.36 μm) showed no significant difference compared to the biocompatible control (Tegaderm, 85.42 ± 50.31 μm), indicating minimal inflammatory response and supporting their suitability for long-term implantation.

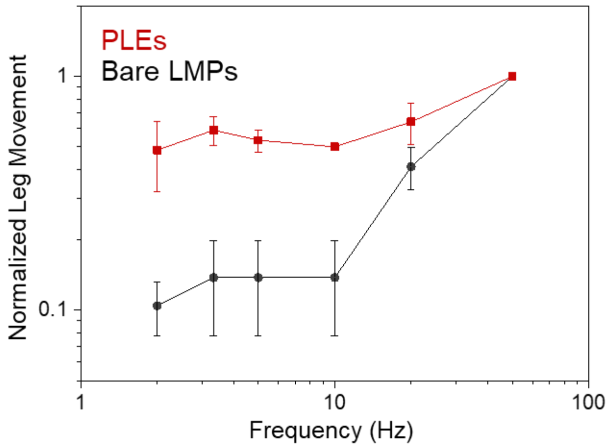

Supplementary Fig. 35| Stimulated leg movement versus stimulation frequency of PLEs versus bare LMPs-based electrodes (n=4 biologically independent samples). Values are presented as means ± SD.

During the experimental procedure, wherein an 80 mV stimulation was administered, the locomotor response of the mice was assessed as a function of varying stimulation frequencies.

REFERENCES

1 Goff, A. *et al.* An exploration into two-dimensional metal oxides, and other 2D materials, synthesised via liquid metal printing and transfer techniques. *Dalton Transactions* **50**, 7513-7526 (2021).

2 Castilla-Amoros, L., Chien, T.-C. C., Pankhurst, J. R. & Buonsanti, R. Modulating the reactivity of liquid Ga nanoparticle inks by modifying their surface chemistry. *Journal of the American Chemical Society* **144**, 1993-2001 (2022).

3 Ermoline, A. & Dreizin, E. L. Equations for the Cabrera–Mott kinetics of oxidation for spherical nanoparticles. *Chemical Physics Letters* **505**, 47-50 (2011).

4 Zhdanov, V. P. & Kasemo, B. Cabrera–Mott kinetics of oxidation of nm-sized metal particles. *Chemical Physics Letters* **452**, 285-288 (2008).

5 Pearson, R. G. Hard and soft acids and bases. *Journal of the American Chemical society* **85**, 3533-3539 (1963).

6 Pearson, R. G. Acids and Bases: Hard acids prefer to associate with hard bases, and soft acids prefer to associate with soft bases. *Science* **151**, 172-177 (1966).

7 Veerapandian, S. *et al.* Hydrogen-doped viscoplastic liquid metal microparticles for stretchable printed metal lines. *Nature materials* **20**, 533-540 (2021).
