# Supplementary material for: Self-packaged stretchable printed circuits with ligand-bound liquid metal particles in elastomer
Source: Nat Commun. 2025 May 28;16:4944. doi: 10.1038/s41467-025-60118-4 (PMC12119845; doi:10.1038/s41467-025-60118-4)
Supplement: Supplementary file 3 — Transparent Peer Review file [file 41467_2025_60118_MOESM3_ESM.pdf]

nature portfolio

Peer Review File

Self-packaged stretchable printed circuits with ligand-bound liquid metal particles in elastomer

Corresponding Author: Professor Seongjun Park

This file contains all reviewer reports in order by version, followed by all author rebuttals in order by version.

Version 0:

Reviewer comments:

Reviewer #1

(Remarks to the Author)
This study presents a distinctive approach to stretchable electronics by introducing ligand-bound liquid metal particles (LB-LMPs) for the fabrication of self-packaged, multi-layer printed circuit boards. By employing in situ sintering and vertical phase separation, the authors address key challenges in traditional stretchable electronics fabrication, such as complex packaging processes and liquid metal leakage. This method provides a solution that demonstrates high conductivity, mechanical stability, and potential scalability for applications in bioelectronics and smart apparel.
The in situ sintering mechanism, enabled by the suppression of oxide layer formation through amine-functionalized ligands, represents a notable advancement in liquid metal-based technologies. Additionally, the vertical phase separation observed in the LB-LMP/polymer composites enhances electrical insulation and eliminates the need for additional packaging processes. The study further highlights the versatility of this approach by successfully integrating LB-LMPs into various polymers, which suggests potential applicability to areas such as smart textiles and implantable bio-interfaces.
The experimental results are supported by comprehensive characterization techniques, including FTIR, XPS, and SEM analyses, lending confidence to the findings. Overall, this manuscript offers a meaningful contribution to the field of stretchable electronics. Based on the above evaluation, this reviewer recommends publication in Nature Communications after revisions to improve clarity and strengthen certain aspects of the study.
Below are specific suggestions for improvement:
1. Organic acids containing carboxyl groups are often used as antioxidants. Could the authors provide a scientific explanation for why these acids do not appear to reduce the oxide layer thickness in liquid metal particles?
2. In experiments where ligands were added to cyclohexane, was there any observable variation in the dispersion of LMPs?
3. The manuscript would benefit from additional SEM data to confirm whether LMP sintering occurred in the experiments involving ligands added to cyclohexane.
4. Including data that demonstrate vertical phase separation in polymer matrices other than polyurethane would enhance the generalizability of the proposed method.
5. Please provide more detailed information on the fabrication process for the PEDOT:PSS/LB-LMPs electrode (PLE).

Reviewer #2

(Remarks to the Author)
This article presents a new approach for fabricating stretchable printed circuit boards (S-PCBs) using ligand-assisted in situ sintered liquid metal particles (LB-LMPs). This method enables one-step printing with self-packaging, eliminating the need for additional activation and packaging processes. It demonstrates high electrical and mechanical stability, vertical electrical connectivity, and adaptability to various polymers, showcasing applications in multi-layer circuits, healthcare monitoring devices, and smart textiles. Recommended for publication after the author carefully considers the following questions.
1.The authors claim that the proposed in situ sintering and self-packaging method can be applied to the large-scale printing and fabrication of S-PCBs. However, the description of the printing process in the article is limited. First, complex circuits require high-resolution printing to prevent short circuits. What is the highest achievable resolution of this method? Second, in high-precision printing, by what method is AgF printed onto the LMP surface? How is the precision of printing points ensured, especially in large-scale circuit printing?
2.The authors have not analyzed the relationship between the self-packaging speed and the printing speed. There is a concern that during large-area high-precision printing, the previously printed fine conductive lines may already be fully

packaged before the subsequent lines are printed, making it impossible to create vertical conductive pathways.
3.Although the authors mention nozzle printing in the article, the demonstrations primarily use methods such as screen printing thin films or coating adhesive tapes followed by laser cutting. This approach seems inconsistent with the "printing" theme and merely produces stretchable conductors. In addition, how to ensure that the shape of complex circuits remains unchanged during the transfer process? Especially in the production of high-precision circuits.
4.The nozzle printing mentioned in the article requires optimization of the relationship between ink viscosity and driving pressure. Please provide specific details regarding this optimization.
5.Figures 4c, 4e, and 4h lack scale bars.

Reviewer #3

(Remarks to the Author)
This manuscript introduces ligand-bound liquid metal particles (LB-LMPs) to inhibit oxide growth. The in situ sintering of LB-LMPs within a polymer matrix promotes vertical phase separation, resulting in the automatic formation of a packaging layer. This straightforward process enables large-scale production of stretchable circuits. The work is interesting and offers new insights to the field. However, the mechanism of LB-LMPs prompts phase separation is unclear, and supporting evidence is insufficient. I recommend major revisions before the manuscript can be considered for publication. Detailed comments are provided below:
1. Mechanism and Supporting Evidence: The authors claim that “The utilization of ligand-bound LMPs prompts phase separation within the polymer matrix” (Line 78), but the mechanism is unclear, and the evidence provided is insufficient. And the statement "evaporation-induced desorption of ligands from LMPs is crucial to facilitate interparticle sintering among LMPs" (Line 154) is quite confusing.
(a) Figure 11 shows that ligands remain on the surface after solvent evaporation. This is perplexing, and the authors should provide evidence to support the presence of ligands after evaporation.
(b) How does the absence of thermal treatment preclude ligand detachment? (Line 153)
(c)The manuscript should explain why the ligands would hinder LMPs sintering. (Line 153)
The authors should provide a clear summary of the final mechanism that drives the phase separation of LB-LMPs. Currently, the discussion only describes the phenomena without offering a comprehensive mechanistic explanation.
2. Characterization of Packaging Effectiveness: There is a lack of characterization to support the uniformity and effectiveness of the LB-LMPs/polymer surface encapsulation. The authors should consider performing electrical conductivity tests (e.g., AFM-CAFM) to further validate this. Additionally, concerns arise regarding the encapsulation effectiveness on both sides of the LB-LMPs/polymer. The authors should provide further characterization, such as SEM images or AFM-CAFM, to address this.
3. In Vivo Biocompatibility: As noted in comment 2, there are concerns regarding the long-term biocompatibility of LB-LMPs/polymer. The manuscript should give more evidence related to chronic in vivo compatibility.
4. Interconnection with AgF: The manuscript states that interconnection between LB-LMPs and AgF is achieved after printing AgF on the LB-LMPs/polymer (Lines 189-192). However, given the presence of an insulating polymer layer, it is unclear how the AgF layer facilitates interconnection with the LB-LMPs. Furthermore, in Figure 4b SEM images, the insulating layer is not visible. The authors should provide a detailed explanation of how the AgF printing process removes the insulating polymer layer to enable interconnection with LB-LMPs.
5. Terminology Issues: The term “interface electrodes” (Line 188) and “bio-interfaced electrodes” (Line 249) is not commonly used in the literature. Please consider using more precise and professional terminology.

Reviewer #4

(Remarks to the Author)
The manuscript introduces a novel approach to fabricating stretchable printed circuit boards (S-PCBs) using ligand-bound liquid metal particles (LB-LMPs). The key innovation lies in the in situ sintering and self-packaging of LB-LMPs, which eliminates the need for complex multi-step fabrication processes. The study demonstrates applications of this method in creating multi-layer circuits, bioelectronics, and smart textiles. The authors validate the technique with extensive characterization and present its scalability and versatility across multiple polymer systems. I am positively impressed by the detailed and extensive characterization made by authors, with extensive use of techniques such as FTIR, SEM, and XPS which strengthens the credibility of the findings by providing detailed insight into the mechanism of the composite formation, phase separation and sintering. The developed technology addresses critical challenges in leakage prevention and simplifies multi-layer circuit fabrication, which are crucial for real-world implementation.

On the other hand, there are some shortcomings with the article. I have some concerns regarding some of the conclusions and as well I have major concerns regarding the overall reproducibility. The manuscript lacks sufficient detail for replication. For example, the specifics of the ligand-binding process and parameters for polymer-to-LMPs volume ratios are not comprehensively outlined. Providing clear, step-by-step protocols would enhance reproducibility.
Here is a list of my concerns.

1. There is not enough information on how LB-LMPs without polymer differ from those with polymer, in terms of self-sintering and conductivity.
2. There is not enough information on various types of polymer embedded LB-LMPs. Authors just say various polymers can be used, but
A. They do not specify the exact formulation of each, and step by step preparation.
B. They do not refer precisely which one is used in following characterizations.

C. In Methods, authors mention “in accordance with the desired volume ratio between the polymer and LM”, but what are the values?
D. “Additionally, Hydrophilic PU (D4), PCL, PVA, PBAT, and SMP were dissolved in NMP”. What is formulation and step by step preparation for each?
3. Authors employ Cabrera-Mott theory to explain reduction of oxide layer thickness. But why this theory is valid for Amines but not carboxyl groups? Also the from SI Figure 7, it is difficult to conclude that the thickness is lower for amine group. Moreover, what would happen if we change the NMP for instance to ethanol?
4. Authors claim particle sintering is observed on figure 2f and 2g. However, I am not able to see such sintering. What authors call sintering? IN LMNPs, sintering haens when several particles join each other, eliminating the borders. Do authors have a different definition?
5. Also in figure s10, I don’t see any sintering. Also why the sizes of particles are different? Why LMP with no NMP has smaller size? Were they sonicated same time?
6. Figure s13 is a very nice figure, but should be replicared for LB-LMB
7. The step-by-step and in detail protocol for measurement of conductivity on LB-LMP should be explained. What substrate is used? How the deposition is made, and how the conductivity is measured. For instance a measurement over glass or silicon wafer with no peeling can be interesting.
8. “In contrast, bare LMPs with thick oxide layers exhibit only a marginal descent due to their high density” I didn’t understand.
9. Authors mentioned “Moreover, fabricating the LB-LMPs/polymer film at ambient temperature without thermal treatment precludes ligand detachment, thereby impeding sintering50-52 between LMPs and inhibiting phase separation (Supplementary Fig. 11). This suggests that evaporation-induced desorption of ligands from LMPs is crucial to facilitate interparticle sintering among LMPs.”. However, I didn’t find detailed information about such thermal treatment. Do author refer to protocol of preparation of particles? Is the solution heated? To what temperature. When the polymer is added? Once more these details are necessary to make the work reproducible.
10. Authors mentioned “for high electrical conductivity, achieving 1 Ω in the interconnect without requiring activation, even at small polymer-to-LMPs volume ratios ”. Why do you mean by even at small polymer-to-LMP. Did you mean even at Larger Polymer to-LMPs? Naturally by adding polymer, sintering becomes more difficult. So this sentence doesn’t make much sense. Also what are these ratios? Again details are missing.
11. Authors mention: “Furthermore, by tuning the viscosity of ink through varying polymer contents, various solution process like printing or dip coating become available (Supplementary Fig. 16).” Once more details of each formulation is missing. If authors claim screen printing, dip coating, etc., for EACH method, they need to write down how the ink is prepared step by step, with details of formulation and preparation.
12. I didn’t find detailed information on preparation of the Ag flake ink for VIAs.
13. Some materials do nto have reference. For instance “silicone adhesive film” , “release tape” “Ag flakes”
14. Protocol and details of fim removal through release film is not described
15. Authors mention “To obtain the bottom surface images of the LMPs/polymer films, the films underwent immersion in water for 10 minutes, and were then peeled off without tension”. Peeling without tension might be difficult to achieve. Why not despoiting over glass and obtaining optical image?
16. Actual conductivity in S/M should be reported by measuring sample conductivity and thickness of the printed lines.

Bottom Line:
The manuscript presents an innovative and impactful study with high potential applications in stretchable electronics and bioelectronics. t as well presents excellent characterization in some parts of the work. However, the lack of detailed reproducibility protocols, and some ambiguities in data interpretation need to be addressed to ensure the robustness of the claims.

Version 1:

Reviewer comments:

Reviewer #1

(Remarks to the Author)
I have thoroughly reviewed the authors' responses to both my comments and those provided by other referees. The authors have adequately addressed the majority of the concerns, and I believe that the manuscript is now suitable for publication in Nature Communications.

Reviewer #2

(Remarks to the Author)
The authors have substantially addressed my concerns. I suggest the publication of the revised manuscript.

Reviewer #3

(Remarks to the Author)
The authors have carefully addressed all questions proposed by reviewers, and I believe it is acceptable for publication

now.

Reviewer #4

(Remarks to the Author)
The comments are appropriately addressed

Open Access This Peer Review File is licensed under a Creative Commons Attribution 4.0 International License, which permits use, sharing, adaptation, distribution and reproduction in any medium or format, as long as you give appropriate credit to the original author(s) and the source, provide a link to the Creative Commons license, and indicate if changes were made.
In cases where reviewers are anonymous, credit should be given to 'Anonymous Referee' and the source.
The images or other third party material in this Peer Review File are included in the article's Creative Commons license, unless indicated otherwise in a credit line to the material. If material is not included in the article's Creative Commons license and your intended use is not permitted by statutory regulation or exceeds the permitted use, you will need to obtain permission directly from the copyright holder.
To view a copy of this license, visit https://creativecommons.org/licenses/by/4.0/

Response to Reviewers' Comments

Title: Self-packaged stretchable printed circuits with ligand-bound liquid metal particles in elastomer

Authors: Hyeonyeob Seo, Gun-Hee Lee, Jiwoo Park, Dong-Young Kim, Yeonzu Son, Semin Kim, Kum Seok Nam, Congqi Yang, Joonhee Won, Jae-Young Bae, Hyunjun Kim, Seung-Kyun Kang, Steve Park, Jiheong Kang, and Seongjun Park

Research Article No.: NCOMMS-24-73429-T

We thank the Reviewers for the careful consideration of our manuscript and the suggested demonstration, clarifications, and analyses that further strengthen our work. Following is a summary of our revision made according to the Reviewers' comments.

The Reviewer's comments are in **bold** and revised texts are highlighted.

Reviewer #1

This study presents a distinctive approach to stretchable electronics by introducing ligand-bound liquid metal particles (LB-LMPs) for the fabrication of self-packaged, multi-layer printed circuit boards. By employing in situ sintering and vertical phase separation, the authors address key challenges in traditional stretchable electronics fabrication, such as complex packaging processes and liquid metal leakage. This method provides a solution that demonstrates high conductivity, mechanical stability, and potential scalability for applications in bioelectronics and smart apparel.

The in situ sintering mechanism, enabled by the suppression of oxide layer formation through amine-functionalized ligands, represents a notable advancement in liquid metal-based technologies. Additionally, the vertical phase separation observed in the LB-LMP/polymer composites enhances electrical insulation and eliminates the need for additional packaging processes. The study further highlights the versatility of this approach by successfully integrating LB-LMPs into various polymers, which suggests potential applicability to areas such as smart textiles and implantable bio-interfaces.

The experimental results are supported by comprehensive characterization techniques, including FTIR, XPS, and SEM analyses, lending confidence to the findings. Overall, this manuscript offers a meaningful contribution to the field of stretchable electronics. Based on the above evaluation, this reviewer recommends publication in Nature Communications after revisions to improve clarity and strengthen certain aspects of the study.

Answer. We appreciate the Reviewer for the helpful comment regarding our work. We have revised our work according to the Reviewer's comments. Point-by-point responses are attached below:

1. Organic acids containing carboxyl groups are often used as antioxidants. Could the authors provide a scientific explanation for why these acids do not appear to reduce the oxide layer thickness in liquid metal particles?

Answer. We appreciate the reviewer's question regarding why organic acids containing carboxyl groups do not reduce the thickness of the oxide layer on liquid metal particles (LMPs).

As the reviewer mentioned, organic acids containing carboxyl groups can bind to the surface of metal particles and suppress oxidation reactions, as explained by the Cabrera-Mott theory. However, the behavior of these ligands on the surface of LMPs is more complex and can be understood through the Hard and Soft Acids and Bases (HSAB) theory.

According to HSAB theory, carboxyl groups, being harder bases, have a higher affinity for the hard acid Ga^{3+} ions compared to softer bases like amine groups[1,2]. When carboxyl groups interact with the Ga^{3+} ions on the LMP surface, they can enhance the outward diffusion of Ga^{3+} ions. This outward diffusion promotes further oxide layer formation[3]. In contrast, the softer base, the amine group, is less likely to facilitate the outward diffusion of Ga^{3+} ions, thereby suppressing further oxide layer formation. This distinction highlights the role of chemical interactions at the molecular level in determining the behavior of oxide layer formation on LMP surfaces.

To provide additional information, we have revised the Supplementary Information section as detailed below:

Revised Supplementary Information

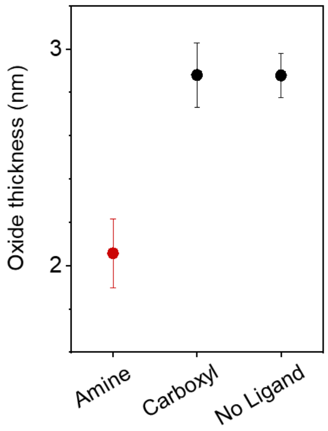

Supplementary Fig. 7| Oxide layer thickness of LMPs depending on the functional group of the ligand.

Each ligand bound LMP as synthesized by sonication in cyclohexane in the presence of the ligand. The ligand featuring an amine group reduce the oxide layer thickness of LMPs.

According to HSAB theory, carboxyl groups, being harder bases, have a higher affinity for the hard acid Ga^{3+} ions compared to softer bases like amine groups[5,6]. When carboxyl groups attach to the Ga^{3+} ions on the LMP surface, they can enhance the outward diffusion of Ga^{3+} ions. This outward diffusion promotes further oxide layer formation[2]. In contrast, the softer base, the amine group, is less likely to facilitate the outward diffusion of Ga^{3+} ions, thereby preventing further oxide layer formation. This distinction highlights the role of chemical interactions at the molecular level in determining the behavior of oxide layer formation on LMP surfaces.

References

1 Pearson, Ralph G. Hard and soft acids and bases. *Journal of the American Chemical society*, **85(22)**, 3533-3539 (1963).

2 Pearson, Ralph G. Acids and Bases: Hard acids prefer to associate with hard bases, and soft acids prefer to associate with soft bases. *Science*, **151(3707)**, 172-177 (1966).

3 Castilla-Amoros, Laia, et al. Modulating the reactivity of liquid Ga nanoparticle inks by modifying their surface chemistry. *Journal of the American Chemical Society*, **144.4**, 1993-2001 (2022).

2. In experiments where ligands were added to cyclohexane, was there any observable variation in the dispersion of LMPs?

Answer. We appreciate the reviewer's insightful comment regarding the impact of ligands on the dispersion of liquid metal particles (LMPs) in cyclohexane. The ligands used in our experiments are chemical compounds with a single functional group (e.g., carboxyl or amine). The functional group anchors to the surface of the LMP, while the opposite end of the compound features a hydrophobic region that interfaces with the nonpolar cyclohexane solvent. This characteristic of the ligands plays a critical role in stabilizing the dispersion of LMPs in cyclohexane. Specifically, the functional group ensures strong attachment to the LMPs, while the hydrophobic segment ensures compatibility with the surrounding solvent, suppressing aggregation.

As illustrated in the accompanying figure, solutions with added ligands such as butylamine or butyric acid demonstrated significantly improved dispersion compared to those without ligands.

To provide additional information, we have revised the Supplementary Information section as detailed below:

Revised Supplementary Information

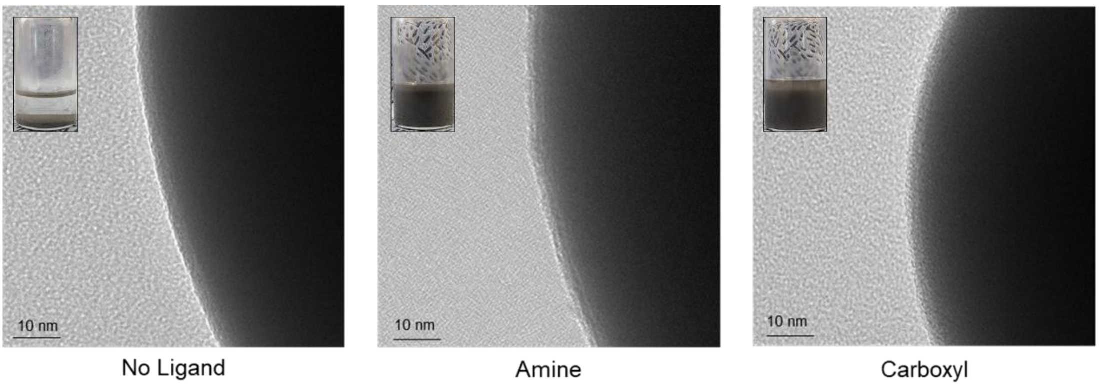

Supplementary Fig. 8| TEM images of LMP surface depending to the functional groups of the ligand.

TEM images capturing the surface morphology of LMP subsequent to ultrasonication in cyclohexane, while incorporating ligands, were obtained. Additionally, the solution containing the ligand exhibited significantly enhanced dispersion compared to the solution without the ligand.

3. The manuscript would benefit from additional SEM data to confirm whether LMP sintering occurred in the experiments involving ligands added to cyclohexane.

Answer. We sincerely appreciate the reviewer's suggestion to include additional SEM data to confirm whether LMP sintering occurred in the experiments involving ligand addition to cyclohexane. In our experiments, ligands were added to cyclohexane during LMP synthesis, and TEM analysis (Supplementary Figures 7 and 8) showed a decrease in the oxide layer thickness specifically in the case of the amine-functionalized ligand. However, SEM analysis confirmed that no sintering of LMPs occurred under these conditions.

These findings suggest that LMP sintering is influenced not only by the oxide layer thickness but also by the solvent evaporation process. Effective LMP sintering requires the desorption of ligands from the particle surface. Cyclohexane, due to its low surface tension[1,2], can not sufficiently facilitate ligand desorption, thereby hindering the sintering process.

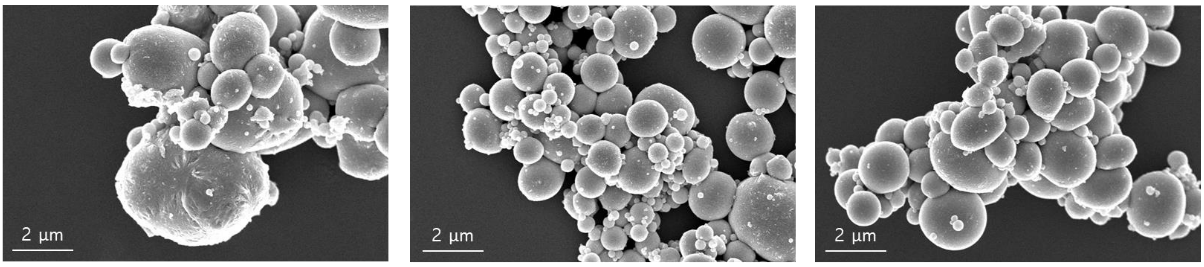

Cyclohexane   Cyclohexane + Amine   Cyclohexane + Carboxyl

References

1 Villares, A., et al. Study of the surface tensions of cyclohexane or methylcyclohexane with some cyclic ethers. Journal of solution chemistry, 34, 185-198 (2005).

2 Occhiuzzi, Jessica, et al. "The quest for green solvents for the sustainable production of nanosheets of two-dimensional (2D) materials, a key issue in the roadmap for the ecology transition in the flatland." Molecules 28(3), 1484 (2023).

4. Including data that demonstrate vertical phase separation in polymer matrices other than polyurethane would enhance the generalizability of the proposed method.

Answer. We appreciate the reviewer's suggestion to provide additional data demonstrating vertical phase separation in polymer matrices other than polyurethane, as this would strengthen the generalizability of our proposed method. To address this, we performed SEM analysis on LB-LMPs/PCL films fabricated using polycaprolactone (PCL). For comparison, we also analyzed bare LMP/PCL films prepared by mixing bare LMP with tip sonication in an ice bath, as described in the main text.

The results confirmed that LB-LMPs/PCL films exhibited vertical phase separation, consistent with the behavior observed in TPU-based films. Specifically, in LB-LMPs/PCL films, the LMPs were encapsulated with polymer at the top surface and segregated toward the bottom surface, indicating distinct vertical phase separation. In contrast, the bare LMPs/PCL films showed LMPs distributed on the top surface, and vertical phase separation was not observed.

These findings demonstrate that the vertical phase separation behavior of LB-LMPs is not limited to TPU but extends to other polymer matrices soluble in NMP, such as PCL, highlighting the broader applicability of our method.

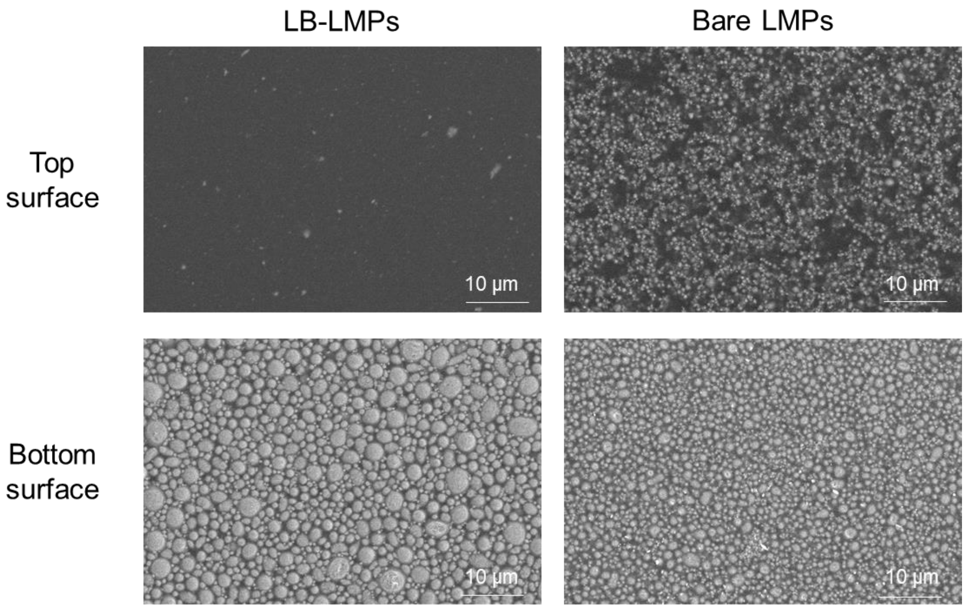

Supplementary Fig. 10| SEM images on the top and bottom surface of the LB-LMPs/polymer and bare LMPs/polymer films.

5. Please provide more detailed information on the fabrication process for the PEDOT:PSS/LB-LMPs electrode (PLE).

Answer. We appreciate the reviewer's request for a more detailed description of the method used to fabricate the PEDOT:PSS/LB-LMPs electrode (PLE). The fabrication process begins with pouring the LB-LMPs/TPU ink onto a glass plate. Then, the PEDOT:PSS/TPU ink is carefully printed on top of the first layer. The use of NMP as a common solvent for both inks ensures high wettability, while incorporating TPU as a common polymer promotes effective adhesion between the PEDOT:PSS and LB-LMPs layers following solvent evaporation. This process results in the vertical alignment of the LMPs and PEDOT:PSS, establishing electrical connectivity throughout the structure and forming the PEDOT:PSS/LB-LMPs electrode (PLE). The bilayer structure of the PLE is characterized by SEM, as shown in Supplementary Fig. 32, while its electrical connectivity is confirmed through impedance measurements presented in Supplementary Fig. 33.

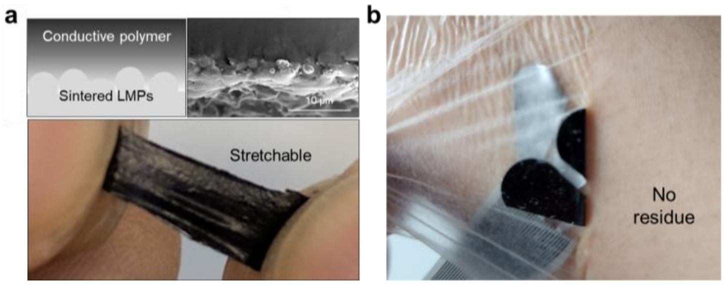

Supplementary Fig. 32| Bilayer coating for a PLE. a, Photograph of the PLE with cross-sectional SEM image. **b**, Photograph of the PLE on skin. There are no leakage or residue during usage.

To provide accurate information, we have revised the Supplementary Information and Method section as detailed below:

Revised Supplementary Information

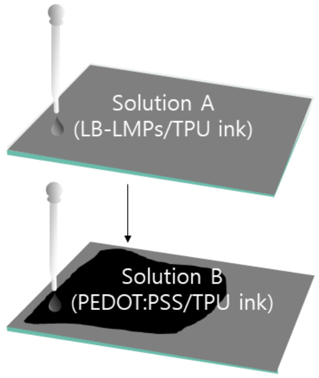

Supplementary Fig. 31| Schematic illustration of electrical connection on top layer. For electrical connection on top layer, PEDOT:PSS/TPU ink was dropped onto the LB-LMPs/TPU ink prior to full evaporation of ink.

Revised Main text

Fabrication of PEDOT:PSS/LB-LMPs electrode (PLE). The fabrication process begins by depositing the LB-LMPs/TPU ink onto the substrate on 60°C hot plate. Subsequently, the PEDOT:PSS/TPU ink is precisely printed on top of the initial layer before the complete evaporation of NMP. The ink is then dried at 60°C and laser-cut into the desired design.

Reviewer #2

This article presents a new approach for fabricating stretchable printed circuit boards (S-PCBs) using ligand-assisted in situ sintered liquid metal particles (LB-LMPs). This method enables one-step printing with self-packaging, eliminating the need for additional activation and packaging processes. It demonstrates high electrical and mechanical stability, vertical electrical connectivity, and adaptability to various polymers, showcasing applications in multi-layer circuits, healthcare monitoring devices, and smart textiles. Recommended for publication after the author carefully considers the following questions.

Answer. We appreciate the Reviewer's insightful feedback on our work. We have addressed the comments thoroughly and incorporated the necessary revisions. Detailed point-by-point responses are provided below:

1.The authors claim that the proposed in situ sintering and self-packaging method can be applied to the large-scale printing and fabrication of S-PCBs. However, the description of the printing process in the article is limited. First, complex circuits require high-resolution printing to prevent short circuits.
What is the highest achievable resolution of this method?
Second, in high-precision printing, by what method is AgF printed onto the LMP surface? How is the precision of printing points ensured, especially in large-scale circuit printing?

Answer. We appreciate the reviewer's questions about the resolution and AgF printing for electrical circuit.

What is the highest achievable resolution of this method? LB-LMP/polymer inks are well-suited for large-area and customizable processes such as nozzle printing and screen printing, making them highly versatile for various applications. At present, our printing process achieves a reliable circuit resolution of approximately 200 μm. However, higher-resolution circuits may require advanced printing techniques, which we plan to explore in future work. We believe that further research into optimizing ink viscosity and refining printing parameters could enable resolutions below 200 μm, opening new possibilities for more precise circuit fabrication.

Second, in high-precision printing, by what method is AgF printed onto the LMP surface? How is the precision of printing points ensured, especially in large-scale circuit printing?

We achieved VIAs by printing AgF/polymer ink onto the LB-LMP/polymer layer before completing the encapsulation process. The success of this method depends on precise control of the viscosity of each ink. The viscosity of the ink was controlled by adjusting the polymer-to-solvent ratio. The optical images provided illustrate the outcomes of printing AgF inks with

varying viscosities onto screen-printed LB-LMP/polymer layers. The images clearly show that low-viscosity AgF/polymer ink tends to spread excessively, hindering precise and well-defined printing.

These results highlight the critical role of optimizing AgF ink viscosity for precise and effective printing. High-viscosity AgF/polymer ink was selectively applied to areas designated for VIAs and conductive pads. Regions without AgF/polymer ink remained insulated on the surface due to the polymer encapsulation achieved through self-packaging, while regions with AgF/polymer ink established effective connections with the LMPs via diffusion.

To improve clarity and provide additional context, we have updated the Supplementary Information section as detailed below:

Revised Supplementary Information

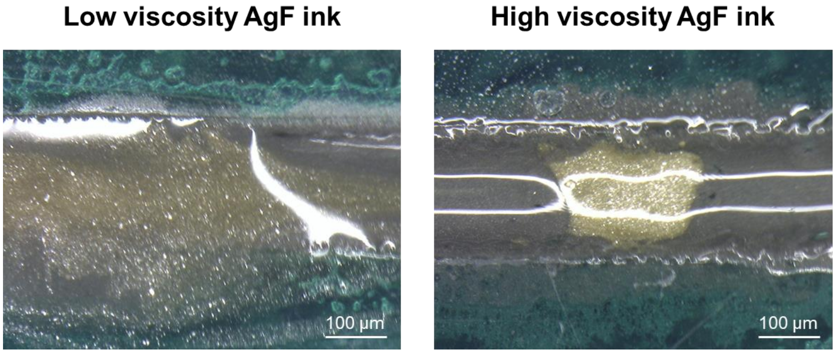

Supplementary Fig. 22| Optical images of printed results on screen-printed LB-LMP/Polymer ink layers at different AgF ink viscosities.
Low-viscosity AgF ink spreads uncontrollably, mixing with the base layer, while high-viscosity AgF ink maintains precise deposition.

Revised Supplementary Information

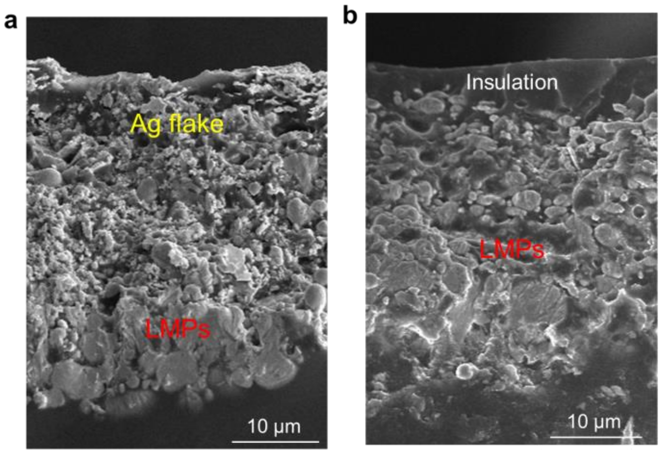

Supplementary Fig. 24| Cross-sectional SEM images of LB-LMPs/polymer conductors (a) with and (b) without AgF printing.

In regions where AgF was printed, vertical connections between AgF and LMPs were established, while in regions without AgF, LMPs were effectively insulated by the polymer layer.

2.The authors have not analyzed the relationship between the self-packaging speed and the printing speed. There is a concern that during large-area high-precision printing, the previously printed fine conductive lines may already be fully packaged before the subsequent lines are printed, making it impossible to create vertical conductive pathways.

Answer. We appreciate the Reviewer's comment regarding the self-packaging and the formation of vertical conductive pathway.

In this article, we focus on the ligand-LMP interaction to enable both self-sintering and self-packaging. To achieve this, we use NMP (N-methyl-2-pyrrolidone) as the solvent, which facilitates ligand decomposition during the ultrasonication process. Since NMP is a high-boiling-point solvent, solvent evaporation at 60°C on a hotplate is required to complete the packaging process. As the packaging step occurs after the completion of all printing steps, the printing speed and packing speed are independent processes and do not directly influence one another.

We acknowledge the need for a more detailed explanation of the process by which the vertical conductive path is formed. To create vertical conductive pathways, we utilized a drop of silver flake-polymer (AgF) ink. These vertical conductive paths were formed by applying AgF ink on top of the LB-LMPs/TPU ink, as shown in Fig. 4a and 4b. As described in the Methods section, after printing LB-LMPs/TPU ink onto a glass substrate, AgF ink was applied to define electrically connected areas, such as VIAs or conductive pads.

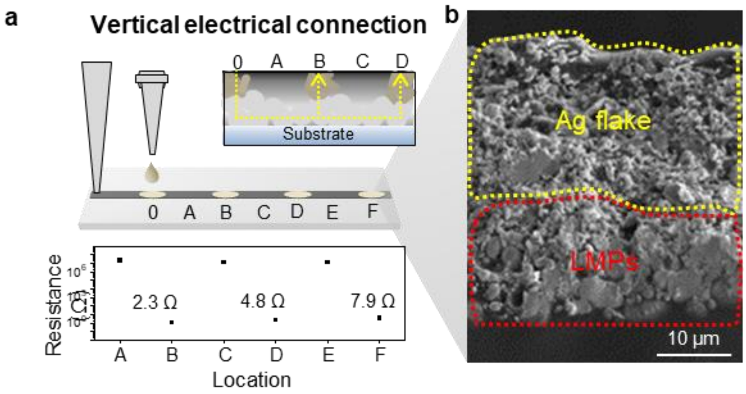

Fig. 4 LB-LMPs-based multi-layered stretchable circuit.
a, Schematic illustration and resistance of the selectively electrically connected LB-LMPs-based conductor with silver flakes. **b**, SEM image of selective electrical connections after printing silver flake composites.

3.Although the authors mention nozzle printing in the article, the demonstrations primarily use methods such as screen printing thin films or coating adhesive tapes followed by laser cutting. This approach seems inconsistent with the "printing" theme and merely produces stretchable conductors. In addition, how to ensure that the shape of complex circuits remains unchanged during the transfer process? Especially in the production of high-precision circuits.

Answer. We appreciate the reviewer's comment on the circuit fabrication and transfer process methods.

Our approach may appear to focus solely on the fabrication of stretchable conductors, but we believe that the key innovation of this study lies in eliminating additional activation and packaging steps. We wanted to emphasize large-area processing and customized manufacturing, which became possible due to this innovation, and to support this, we introduced various printing techniques, including screen printing and laser cutting. Please consider nozzle printing as one of the potential methods for this purpose as well.

To avoid confusion, let us summarize the core idea: conventional circuits utilizing Liquid Metal Particles (LMPs) require additional activation and packaging processes to prevent leakage. However, in this study, we streamlined the fabrication process by employing in situ sintering of LMPs and self-packaging using polymers, enabling the production of complex circuits beyond simple stretchable conductors. In nozzle printing, a resolution of 200 µm is currently achieved, but further advancements to surpass this threshold remain a key area for future research. Our inks possess the shear-thinning properties necessary for nozzle printing, and with precise viscosity control, achieving higher resolutions is a viable goal for future studies.

The method employing these LB-LMPs facilitates vertical phase separation, achieving conductivity even at low LMP volume ratios. A low LMP volume ratio corresponds to a relatively high polymer content, which enhances mechanical stability, a critical factor for the transfer process. Our self-packaged LB-LMP/polymer films demonstrate exceptional mechanical stability, effectively preventing mechanical damage and liquid metal leakage during transfer.

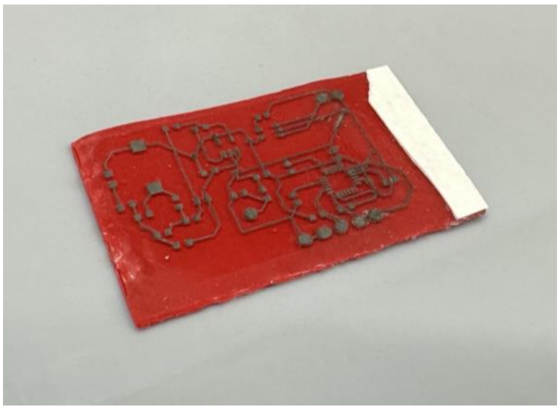

Fig| S-PCB after Transfer Process.

To provide a clearer explanation of our approach, we have revised the Supplementary Information section of the main text as outlined below:

Revised Supplementary Information

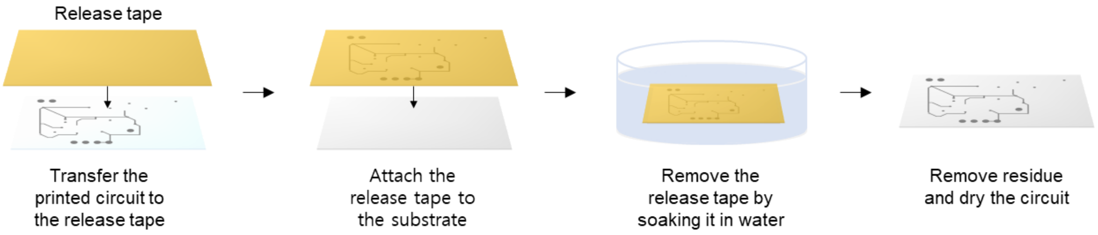

Supplementary Fig. 29| Transfer Process for S-PCB Fabrication.

Printed circuits were immersed in water, allowing for easy removal using release tape. Each printed circuit layer was then sequentially transferred onto the target substrate, enabling the assembly of multi-layer S-PCBs.

4.The nozzle printing mentioned in the article requires optimization of the relationship between ink viscosity and driving pressure. Please provide specific details regarding this optimization.

Answer. We appreciate the reviewer's question regarding the optimization of the relationship between ink viscosity and driving pressure for nozzle printing.

The shear-thinning behavior of ink is a crucial factor in nozzle printing. To assess its suitability for nozzle-based printing, we analyzed its shear-thinning properties. Rheological analysis confirmed that the prepared LB-LMPs/TPU ink exhibited shear-thinning behavior across various concentrations (TPU in NMP, 50 mg/mL to 90 mg/mL, with an LMP volume fraction of 25%). These properties validate the ink's compatibility with nozzle-based printing.

As the reviewer mentioned, achieving optimal printing quality also depends on the driving pressure and printing speed. We performed nozzle printing experiments using a driving pressure of 80–120 kPa and a printing speed of 1600 mm/min. The viscosity of LB-LMPs/TPU ink was controlled by varying the TPU concentration while maintaining a constant LMP volume fraction (25%). The experiments were performed using 27G stainless steel dispensing tips. The results showed that ink with a concentration of 50 mg/ml spread to approximately three times the nozzle diameter, while ink with a concentration of 90 mg/ml resulted in discontinuous printing. The ink with a concentration of 70 mg/ml achieved successful printing, producing circuits with a resolution of 200 μm.

To further demonstrate its printing capability, we have included relevant data in the Supplementary Information.

Revised Supplementary Information

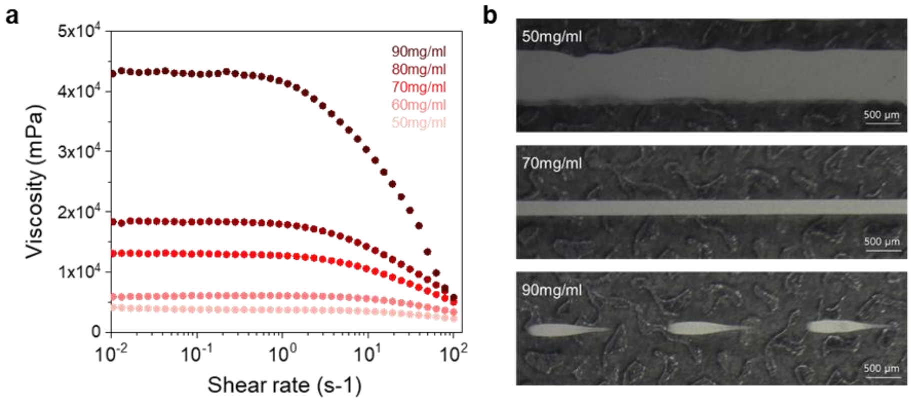

Supplementary Fig. 18| Rheological behavior and printing performance of LB-LMPs/TPU Inks. a, Shear-rate-dependent viscosity of LB-LMPs/TPU inks. **b,** Printed circuit lines using nozzle-based printing at varying ink concentrations.

LB-LMPs/TPU inks exhibited shear-thinning behavior across TPU/NMP concentrations ranging from 50 mg/mL to 90 mg/mL, confirming their suitability for nozzle-based printing.

Nozzle-based printing was conducted at a driving pressure of 80–120 kPa and a printing speed of 1600 mm/min using a 27G stainless steel jetting tip. The ink with a concentration of 50 mg/mL spread to approximately three times the nozzle diameter, whereas the 90 mg/mL ink led to discontinuous printing. In contrast, the 70 mg/mL ink enabled stable printing, achieving a circuit resolution of 200 μm.

5.Figures 4c, 4e, and 4h lack scale bars.

Answer. We appreciate your valuable feedback regarding the lack of scale bars in Figures 4c, 4e, and 4h. Scale bars have now been added to these figures to enhance clarity and ensure the accuracy of the information presented. The updated figures are included in the revised main text as shown below:

Revised Figure

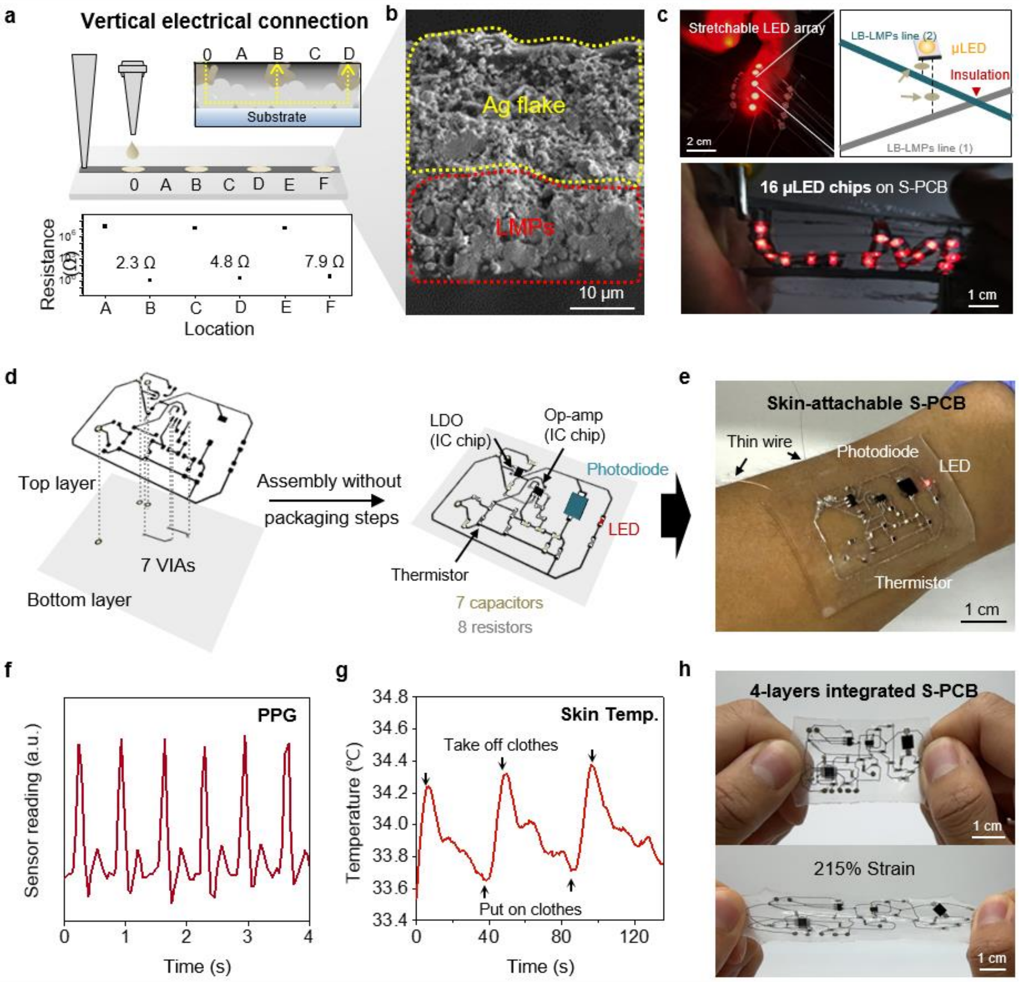

Fig. 4 LB-LMPs-based multi-layered stretchable circuit.
a, Schematic illustration and resistance of the selectively electrically connected LB-LMPs-based conductor with silver flakes. **b**, SEM image of selective electrical connections after printing silver flake composites. **c**, Optical images and schematic illustration of the LED array under strain. **d**, Schematic illustration of the S-PCB with conductive traces, VIAs, and electronic components using LB-LMPs for PPG and temperature sensing. **e**, Optical image of the PPG sensor attached to human skin. **f**, The normalized reading of the PPG sensor on the wrist. **g**, Measured skin temperature change on the wrist. **h**, Optical image of a stand-alone 4-layer integrated S-PCB (top) and the S-PCB under 215% strain (bottom).

Reviewer #3

This manuscript introduces ligand-bound liquid metal particles (LB-LMPs) to inhibit oxide growth. The in situ sintering of LB-LMPs within a polymer matrix promotes vertical phase separation, resulting in the automatic formation of a packaging layer. This straightforward process enables large-scale production of stretchable circuits. The work is interesting and offers new insights to the field. However, the mechanism of LB-LMPs prompts phase separation is unclear, and supporting evidence is insufficient. I recommend major revisions before the manuscript can be considered for publication. Detailed comments are provided below:

Answer. We thank the Reviewer for the valuable suggestions and constructive feedback. In response, we have revised our manuscript accordingly, and detailed point-by-point responses are included below:

1. Mechanism and Supporting Evidence: The authors claim that “The utilization of ligand-bound LMPs prompts phase separation within the polymer matrix” (Line 78), but the mechanism is unclear, and the evidence provided is insufficient. And the statement "evaporation-induced desorption of ligands from LMPs is crucial to facilitate interparticle sintering among LMPs" (Line 154) is quite confusing.

(a) Figure 11 shows that ligands remain on the surface after solvent evaporation. This is perplexing, and the authors should provide evidence to support the presence of ligands after evaporation.

(b) How does the absence of thermal treatment preclude ligand detachment? (Line 153)

(c)The manuscript should explain why the ligands would hinder LMPs sintering. (Line 153)

The authors should provide a clear summary of the final mechanism that drives the phase separation of LB-LMPs. Currently, the discussion only describes the phenomena without offering a comprehensive mechanistic explanation.

Answer. We appreciate the Reviewer’s careful review regarding the mechanism and supporting evidence for phase separation and ligand behavior in LB-LMPs. Below, we provide a clarified and comprehensive explanation addressing each point raised.

We propose that ligand desorption is driven by solvent evaporation with heat, as evidenced by comparative experiments conducted with and without heat treatment. In the absence of heat treatment, ligand desorption is not induced, and consequently, sintering between LMPs does not occur. This was confirmed by experiments showing that LB-LMP films prepared without heat treatment failed to exhibit both sintering and vertical phase separation (Supplementary Fig. 11).

(a) Evidence of ligands remaining after solvent evaporation (Figure 11):

Supplementary Fig. 11 demonstrates that sintering and vertical phase separation do not occur in the LB-LMPs/polymer film when the solvent is separated at room temperature. This absence of activity can be attributed to the fact that ligands are not desorbed during the evaporation process. To investigate this phenomenon in greater detail, we conducted XPS analysis on LB-LMPs/PS films using polystyrene (PS), a polymer composed solely of carbon and hydrogen. XPS measurements were performed on the bottom layer of two types of films: LB-LMPs/PS films prepared by heat treatment to evaporate the solvent and LB-LMPs/PS films prepared by solvent separation at room temperature. In the N 1s spectrum, a peak at 399.5 eV was observed in the LB-LMPs/PS films prepared by solvent separation at room temperature, which was absent in the films prepared by solvent evaporation through heat treatment. This observation indicates that amine-based ligand desorption remains incomplete in the absence of solvent evaporation with heat treatment, leaving residual ligands on the LMP surface. These results emphasize that the dynamics of solvent evaporation significantly influence ligand behavior, directly affecting in situ sintering and the initiation of vertical phase separation.

(b) Ligand separation without heat treatment (line 153):

In the absence of heat treatment, solvent evaporation is absent, and the heat and capillary forces required to induce ligand desorption are insufficient. For the LB-LMPs/polymer films fabricated without heat treatment, it was confirmed that the ligands remained on the surface of the LMPs. Without ligand desorption, the ligands remain on the LMP surface, acting as a barrier that physically blocks the diffusion pathways of the LMP surface atoms. This interference prevents interactions between adjacent LMPs, thereby inhibiting sintering and the formation of conductive pathways.

(c) Why ligands hinder LMP sintering (line 153):

When ligands remain on the surface, they physically isolate the LMPs, preventing direct contact between the metal particle surfaces. This isolation blocks the diffusion and interaction of surrounding metal particles, which are essential for the sintering process. The absence of ligand desorption disturbs with the sintering of LMPs and the formation of conductive paths, while also interfering with vertical phase separation.

Final mechanism driving phase separation in LB-LMPs:

As ligands desorb due to capillary force during solvent evaporation, the dispersion of LMPs in the polymer solution decreases, initiating vertical phase separation. The LB-LMPs, characterized by a thin oxide layer, undergo in-situ sintering upon ligand desorption, forming conductive pathways and exhibiting initial conductivity. This sintering accelerates vertical phase separation, allowing LMPs to migrate to the bottom layer while the polymer self-packages into the top layer. This self-packaging creates a stable structure where the conductive bottom layer is electrically isolated by the polymer encapsulation in the top layer.

We hope this expanded explanation provides clarity and addresses the reviewer's concerns. Thank you for highlighting the need for a more detailed discussion.

To provide additional information, we have revised the Main text and Supplementary Information section as detailed below:

Revised Main text

Moreover, fabricating the LB-LMPs/polymer film at ambient temperature without thermal treatment precludes ligand detachment, thereby impeding sintering[50-52] between LMPs and inhibiting phase separation (**Supplementary Fig. 11**). XPS analysis of LB-LMP/polymer films without heat treatment reveals a peak at 399.5 eV in the N 1s spectrum, indicating incomplete ligand desorption (**Supplementary Fig. 12**). This suggests that evaporation-induced desorption of ligands from LMPs is crucial to facilitate interparticle sintering among LMPs and initiate vertical phase separation.

Revised Supplementary Information

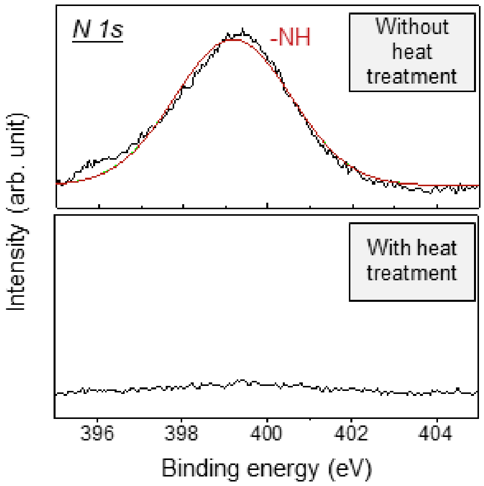

Supplementary Fig. 12| XPS of N 1s of LB-LMPs/PS film with/without heat treatment

To investigate ligand detachment, we conducted XPS analysis on LB-LMPs/PS films using polystyrene (PS), a carbon-hydrogen polymer. XPS measurements were performed on the bottom layer of two film types: those prepared by heat-induced solvent evaporation and those by solvent separation at room temperature. In the N 1s spectrum, a peak at 399.5 eV appeared only in the latter, indicating incomplete desorption of amine-based ligands without heat treatment, leaving residual ligands on the LMP surface.

2. Characterization of Packaging Effectiveness: There is a lack of characterization to support the uniformity and effectiveness of the LB-LMPs/polymer surface encapsulation. The authors should consider performing electrical conductivity tests (e.g., AFM-CAFM) to further validate this. Additionally, concerns arise regarding the encapsulation effectiveness on both sides of the LB-LMPs/polymer. The authors should provide further characterization, such as SEM images or AFM-CAFM, to address this.

Answer. We appreciate the reviewer's comments. The LB-LMPs/polymer films undergo vertical phase separation, with the top surface encapsulated by the polymer and the bottom surface forming a conductive pathway through LMP sintering.

To verify the vertical phase separation, SEM analysis was performed on both the top and bottom surfaces of the LB-LMPs/polymer films. SEM images of the top surface clearly show the encapsulation of LMPs within the polymer matrix, while the bottom surface images reveal a high concentration of LMPs forming a continuous conductive pathway.

To further assess the uniformity of surface encapsulation at the macroscale, peeling tests were conducted on the LB-LMPs/polymer surface (Fig. 3e). The results showed no evidence of LM leakage, providing strong evidence for the effectiveness of the encapsulation. Additionally, conductivity measurements of the LB-LMPs/polymer surface confirmed the absence of conductivity on the encapsulated top surface across different regions, further validating the successful packaging of the LMPs within the polymer layer (Fig. 4a and Supplementary Fig. 23). These findings collectively demonstrate the reliability and uniformity of the encapsulation process.

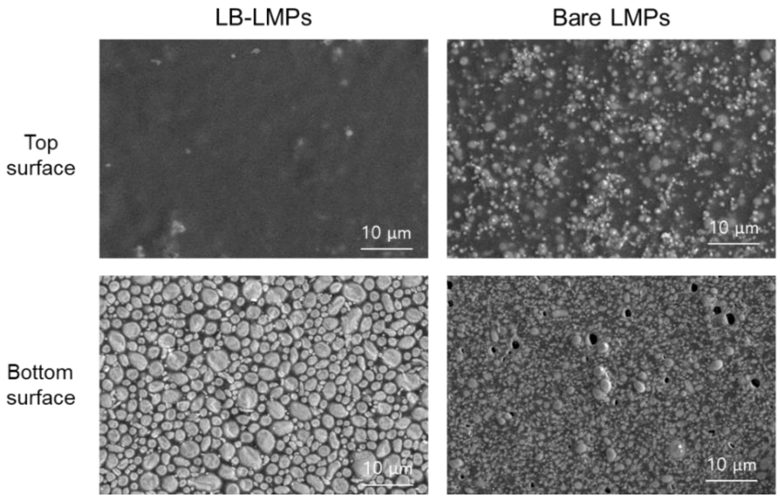

Supplementary Fig. 10| SEM images on the top and bottom surface of the LB-LMPs/polymer and bare LMPs/polymer films.

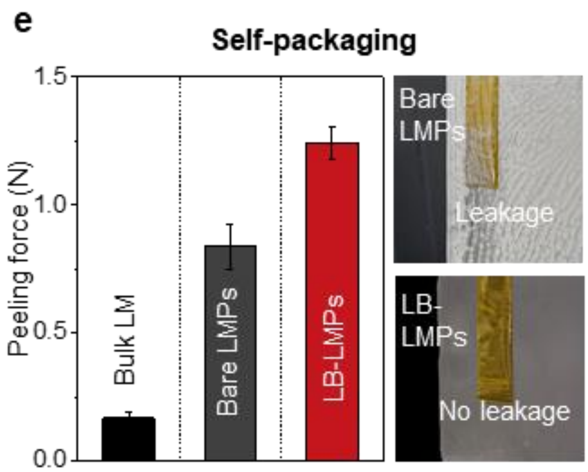

Fig. 3 Mechanical and electrical properties of LB-LMPs-based conductors.
e, Peeling force of bulk LM, bare LMPs/polymer and LB-LMPs/polymer films. There is no leakage of the LB-LMPs/polymer film after peel off with tape.

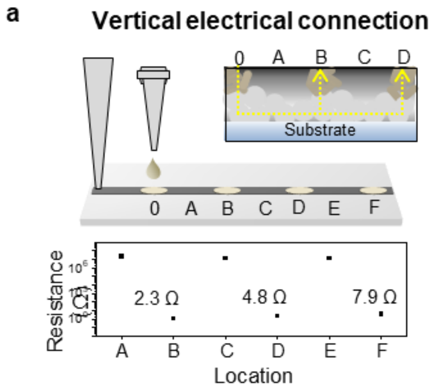

Fig. 4 LB-LMPs-based multi-layered stretchable circuit.
a, Schematic illustration and resistance of the selectively electrically connected LB-LMPs-based conductor with silver flakes.

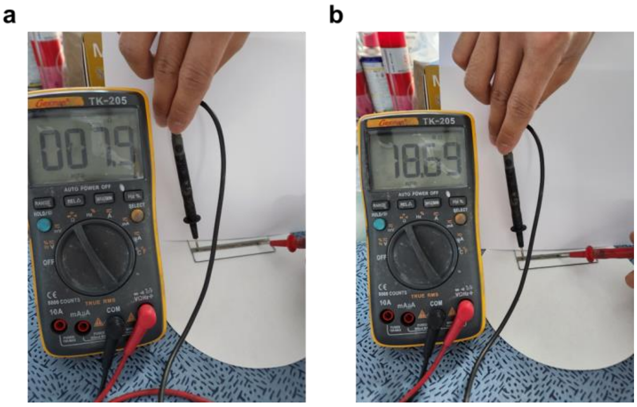

Supplementary Fig. 23| AgF-based selective electrical connection. a, Resistance between AgF connection (7.9 Ohm)**. b,** Resistance between AgF and a packaged layer (18.69 MOhm).

3. In Vivo Biocompatibility: As noted in comment 2, there are concerns regarding the long-term biocompatibility of LB-LMPs/polymer. The manuscript should give more evidence related to chronic in vivo compatibility.

Answer. We appreciate the reviewers' concerns regarding the long-term biocompatibility of LB-LMP/polymer films and acknowledge the importance of providing additional evidence to support their potential use in implantable electronic devices.

To assess the chronic in vivo compatibility of our material, we conducted subcutaneous implantation studies using PEDOT:PSS/LB-LMP electrodes (PLEs) in mice. Tissue responses were evaluated through hematoxylin and eosin (H&E) staining at 2 weeks post-implantation in a subcutaneous pocket on the dorsal side of the animals. The inflammatory response was quantified by measuring the thickness of fibrotic tissue surrounding the implants. As a control, we used a Tegaderm film, a commonly employed biocompatible material. The fibrotic capsule thickness measured around the Tegaderm film was 85.42 ± 41.08 µm, whereas the PLE group exhibited a fibrotic tissue thickness of 75.92 ± 24.79 µm, with no statistically significant difference between the two groups.

These findings indicate that PLEs induce a minimal inflammatory response comparable to that of an established biocompatible material, suggesting their suitability for long-term implantation. Furthermore, no signs of significant tissue damage or adverse immune reactions were observed, reinforcing the potential of LB-LMP/polymer films for chronic bioelectronic applications.

To provide additional information, we have revised the Main test, Supplementary Information and Method section as detailed below:

Revised Main Text

Moreover, LMPs-based implantable devices have recently gained attention for their low modulus and high electrical conductivity. However, the risk of leakage significantly hinders their broader application, necessitating extra packaging or treatments. Our PLE, leveraging PEDOT:PSS and LB-LMPs, overcomes this issue by providing highly stable and biocompatible interfaces. In addition, compared to the bare LMPs-based electrode, the PLE interface improves cell viability due to the sealing of LMPs surfaces with a biocompatible PEDOT:PSS[58-60] (**Fig. 5d** and **5e**). In vivo biocompatibility was evaluated through dorsal subcutaneous implantation in mice for 2 weeks, revealing a minimal inflammatory response comparable to a biocompatible control, supporting their potential for long-term implantation (**Supplementary Fig. 34**).

Revised Supplementary Information

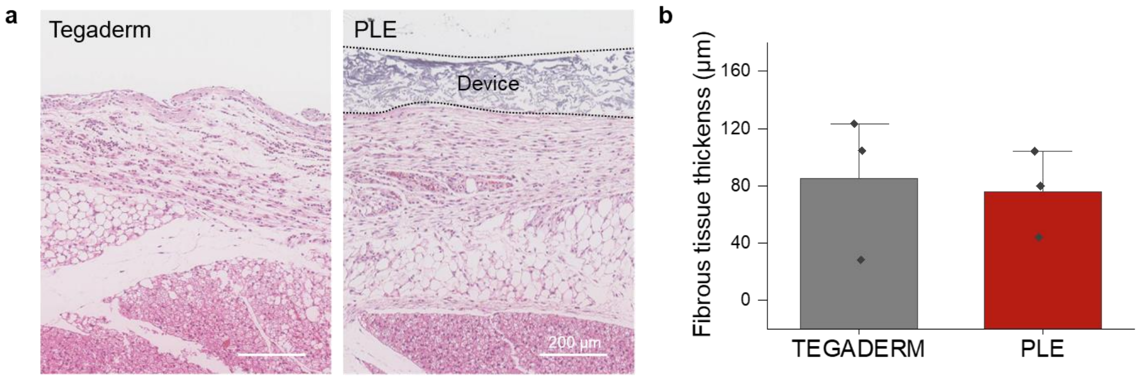

Supplementary Fig. 34| Long-term in vivo biocompatibility of PLE. a, Histological evaluation of subcutaneous implantation of PLE and Tegaderm. **b,** Quantification of fibrotic response of PLE and Tegaderm.

The in vivo biocompatibility of PLEs was evaluated through subcutaneous implantation in mice, followed by H&E staining at 2 weeks post-implantation. The fibrotic tissue thickness around PLEs (75.92 ± 24.79 µm) showed no significant difference compared to the biocompatible control (Tegaderm, 85.42 ± 41.08 µm), indicating minimal inflammatory response and supporting their suitability for long-term implantation.

Revised Main text

In vivo biocompatibility of PLE. Surgical procedures. All animal surgeries were reviewed and approved by the Institutional Animal Care and Use Committee at Seoul National University Hospital. 8-weeks old male B6 mice were used for all in vivo studies. PLE films were cut into 5 mm X 5 mm and attached to Tegaderm for easy handling. Tegaderm was cut into 5 mm X 5mm for implantation as control group. The films were prepared using sterile techniques and further sterilized by immersing 100% Ethanol for 1 h and further sterilized under UV light for 30 min. The films were implanted in the dorsal subcutaneous pocket of the animal. Mice were anesthetized using isoflurane during implantation surgery.

Back hair was removed, and skin was sterilized with betadine. A 1-cm Incision was made carefully with surgical scissors. The films were inserted into the subcutaneous pocket, and the skin incisions were closed with sutures (5-0 Black silk, Ethicon). After 2 weeks of implantation, the animals were euthanized by CO2 inhalation and the skin under the film was dissected for histological analysis. The tissues were then fixed in 10% formalin for 24h before paraffin-embedding. The paraffin-embedded fixed samples were sliced and prepared into slides. The slides were deparaffinized and underwent hematoxylin and eosin staining for histological analysis.

4. Interconnection with AgF: The manuscript states that interconnection between LB-LMPs and AgF is achieved after printing AgF on the LB-LMPs/polymer (Lines 189-192). However, given the presence of an insulating polymer layer, it is unclear how the AgF layer facilitates interconnection with the LB-LMPs. Furthermore, in Figure 4b SEM images, the insulating layer is not visible. The authors should provide a detailed explanation of how the AgF printing process removes the insulating polymer layer to enable interconnection with LB-LMPs.

Answer. We appreciate the reviewer's questions regarding the interconnection between LB-LMPs and AgF, as well as the role of the insulating polymer layer. As noted, once the solvent has fully evaporated and the LB-LMP/polymer layer is completely encapsulated by the polymer, direct interconnection with AgF becomes impossible. To overcome this, we achieved interconnection by printing AgF/polymer ink onto the LB-LMP/polymer layer before the encapsulation process was complete. Since both inks use the same solvent (NMP) and polymer (TPU), they exhibit excellent wettability and adhesion, enabling the AgF/polymer ink to diffuse and establish effective connections with the LB-LMPs.

To successfully implement this method, precise viscosity control of the inks is crucial. The AgF/polymer ink applied later should have relatively high viscosity to prevent it from mixing excessively into the LB-LMPs/polymer ink. The optical images provided illustrate the outcomes of printing AgF/polymer inks with varying viscosities onto screen-printed LB-LMPs/polymer inks. These results underscore the importance of using AgF/polymer inks with optimal viscosity for precise and effective printing.

High-viscosity AgF/polymer ink was applied selectively to areas designated for VIAs and conductive pads. Areas without AgF/polymer ink remained insulated due to the polymer encapsulation from self-packaging, while areas with AgF/polymer ink formed effective connections with the LMPs through diffusion. This behavior was verified through SEM analysis. The SEM results indicate that the LB-LMPs/polymer layer, with a thickness of approximately 40 µm, has an insulating polymer layer of less than 10 µm on the top. The AgF/polymer ink was observed to penetrate up to about 25 µm, effectively connecting to the LMPs and ensuring electrical interconnection. To provide additional information, we have revised the Supplementary Information section as detailed below:

Revised Supplementary Information

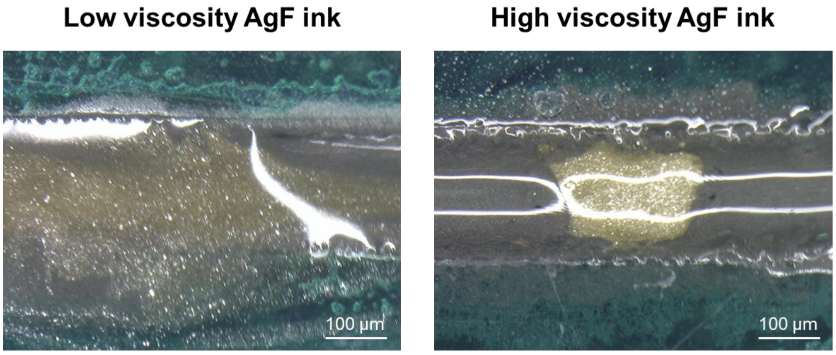

Supplementary Fig. 22| Optical images of printed results on screen-printed LB-LMP/Polymer ink layers at different AgF ink viscosities.

Low-viscosity AgF ink spreads uncontrollably, mixing with the base layer, while high-viscosity AgF ink maintains precise deposition.

Revised Supplementary Information

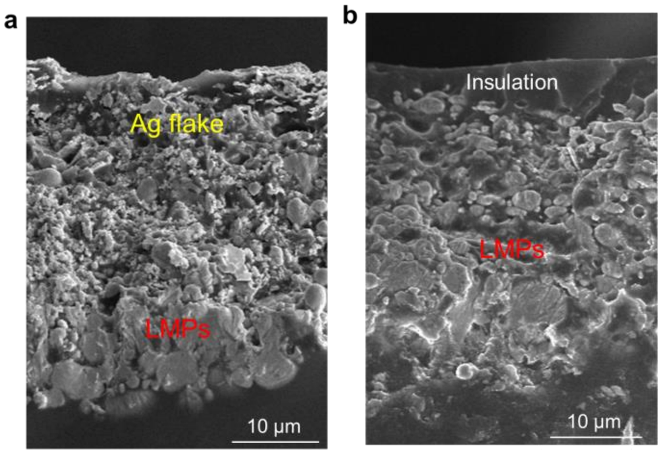

Supplementary Fig. 24| Cross-sectional SEM images of LB-LMPs/polymer conductors (a) with and (b) without AgF printing.

In regions where AgF was printed, vertical connections between AgF and LMPs were established, while in regions without AgF, LMPs were effectively insulated by the polymer layer.

5. Terminology Issues: The term "interface electrodes" (Line 188) and "bio-interfaced electrodes" (Line 249) is not commonly used in the literature. Please consider using more precise and professional terminology.

Answer. We appreciate the reviewer's valuable feedback regarding the terminology. To address this, we have revised the main text to incorporate more precise and widely accepted terms. Specifically, the term "interface electrodes" and "bio-interfaced electrodes" have been replaced with "bioelectronic interfaces" to ensure alignment with standard terminology in the literature. These changes have been reflected in the main text as detailed below:

Revised Main Text

In the pursuit of completing electrical systems, mechanically robust electrodes are essential for seamless integration with electronic components or bioelectronic interfaces[53-55]. However, physically removing the packaged polymer layer inevitably leads to the leakage of liquid metal.

…

The combination of LB-LMPs with various conductive materials through this straightforward process shows versatility and reliable performance as bioelectronic interfaces, highlighting the potential for effective liquid metal use in diverse bio-applications.

Reviewer #4

The manuscript introduces a novel approach to fabricating stretchable printed circuit boards (S-PCBs) using ligand-bound liquid metal particles (LB-LMPs). The key innovation lies in the in situ sintering and self-packaging of LB-LMPs, which eliminates the need for complex multi-step fabrication processes. The study demonstrates applications of this method in creating multi-layer circuits, bioelectronics, and smart textiles. The authors validate the technique with extensive characterization and present its scalability and versatility across multiple polymer systems. I am positively impressed by the detailed and extensive characterization made by authors, with extensive use of techniques such as FTIR, SEM, and XPS which strengthens the credibility of the findings by providing detailed insight into the mechanism of the composite formation, phase separation and sintering. The developed technology addresses critical challenges in leakage prevention and simplifies multi-layer circuit fabrication, which are crucial for real-world implementation.

On the other hand, there are some shortcomings with the article. I have some concerns regarding some of the conclusions and as well I have major concerns regarding the overall reproducibility. The manuscript lacks sufficient detail for replication. For example, the specifics of the ligand-binding process and parameters for polymer-to-LMPs volume ratios are not comprehensively outlined. Providing clear, step-by-step protocols would enhance reproducibility.

Here is a list of my concerns.

Answer. We are grateful to the Reviewer for the thoughtful comments on our work. We have made the requested revisions and provide detailed responses to each point below:

1.There is not enough information on how LB-LMPs without polymer differ from those with polymer, in terms of self-sintering and conductivity.

Answer. We appreciate the reviewer's question regarding the differences between LB-LMPs without polymer and LB-LMPs with polymer in terms of self-sintering and conductivity.

SEM imaging, as shown in Fig. 2f, confirms that localized in-situ sintering can occur in LB-LMPs without polymer. However, in the absence of a polymer matrix, the lack of structural support limits the ability of particles to form consistent interconnections. This hinders the establishment of long-range conductivity. Although localized sintering of LB-LMPs is observed at the microscale, macroscale conductivity remains difficult to achieve without the polymer matrix. In LB-LMPs with polymer, the polymer matrix aids in maintaining particle dispersion and alignment during the sintering process (Fig. 3a). This additional structural support enhances the connectivity of sintered particles, enabling the formation of conductive pathways.

In comparison, bare LMPs exhibit no signs of sintering under the same conditions, regardless of the presence or absence of the polymer. SEM images reveal that bare LMPs retain their perfectly spherical shapes, which is attributed to the presence of a thick oxide layer that prevents interparticle contact and sintering. This highlights the critical role played by the ligand in facilitating sintering, as it reduces the oxide layer and promotes particle-to-particle connections.

These findings underscore the role of the ligand in facilitating effective sintering and the polymer matrix in supporting the establishment of long-range conductivity in LB-LMP systems.

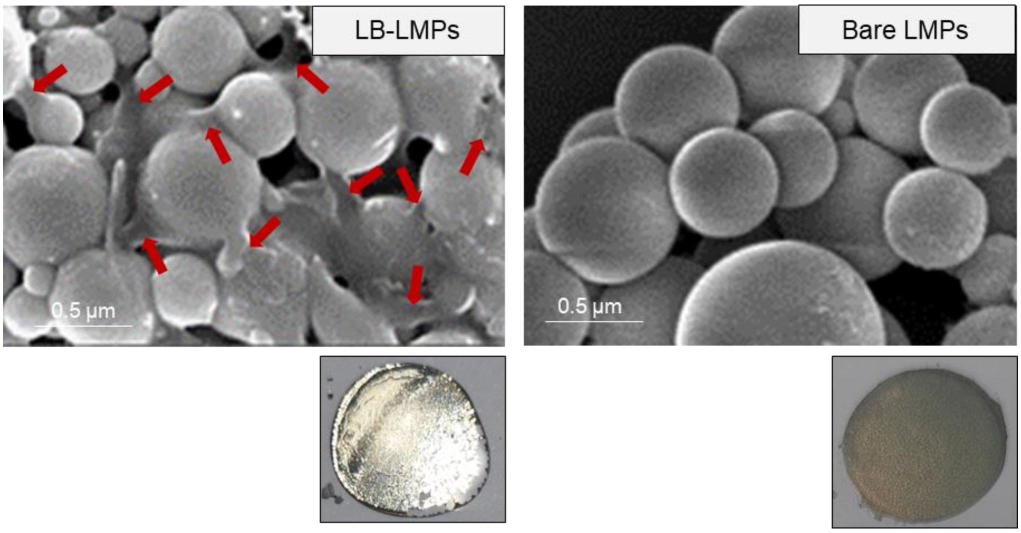

Fig. 2 Mechanism of in situ sintering and self-packaging of LB-LMPs.
f, SEM images of the top surfaces of bare LMPs and LB-LMPs.

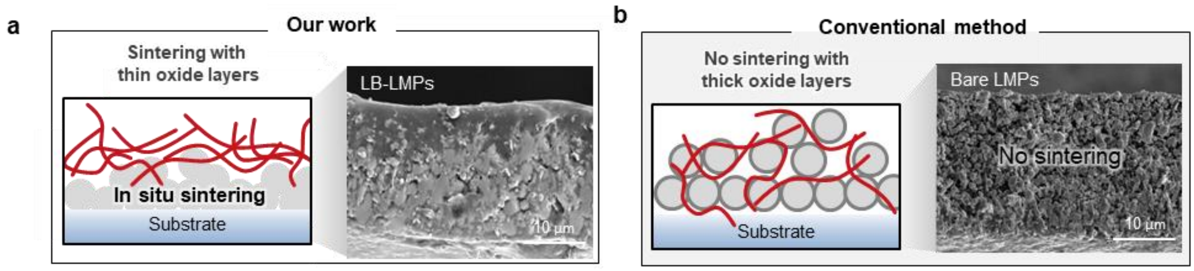

Fig. 3 Mechanical and electrical properties of LB-LMPs-based conductors.
a, Schematic illustration and cross-sectional SEM image of the LB-LMPs/polymer film and **b**, the bare LMPs/polymer film.

2.There is not enough information on various types of polymer embedded LB-LMPs. Authors just say various polymers can be used, but

A.They do not specify the exact formulation of each, and step by step preparation.

B.They do not refer precisely which one is used in following characterizations.

C.In Methods, authors mention "in accordance with the desired volume ratio between the polymer and LM", but what are the values?

D."Additionally, Hydrophilic PU (D4), PCL, PVA, PBAT, and SMP were dissolved in NMP". What is formulation and step by step preparation for each?

Answer. We appreciate the opportunity to clarify the polymer embedded LB-LMPs preparation and characterization process. Below, we provide detailed information to address each point you raised.

In our experiments highlighting the universal applicability of the mechanism, we utilized hydrophilic PU (D4), PCL, PVA, PBAT, and SMP, all of which are known as effective binders for LMP. All these polymers were dissolved in NMP at a uniform concentration of 0.1 g/mL and subsequently mixed with LB-LMP solutions, maintaining a consistent LMP volume fraction of 25%.As a representative example, the preparation process of the LB-LMPs/D4 film is as follows:

1. Dissolve 0.6 g of D4 in 6 ml of NMP to prepare the D4 solution.
2. Add approximately 1.04 g of LM (corresponding to a 25% LMPs volume fraction) to 3 ml of NMP and perform tip sonication to prepare the LB-LMPs solution.
3. Mix the two solutions thoroughly using a Thinky mixer.
4. Evaporate the solvent on a 60°C hotplate to form the film.

To compare resistance based on LMPs volume fractions, films were prepared with volume fractions of 25%, 50%, and 75%, as shown in Fig. 3d. This systematic preparation allows us to evaluate the impact of varying volume fractions on electrical properties.

The subsequent electrical characterizations were conducted using TPU polymer, with data obtained at a polymer-to-LMPs volume fraction of 25%. In the Methods section, when the polymer used was TPU, the term "LMPs/polymer film" was updated to "LMPs/TPU film" for clarity. To provide a clearer explanation of our approach, we have revised the Methods section of the main text as detailed below:

Revised Main Text

Preparation of LB-LMPs/polymer ink. The LB-LMPs solution was incorporated into a

polymer solution (1.2g of TPU in 18mL of NMP), undergoing mixing for a duration of 5 minutes utilizing a Thinky mixer (AR-100). The volume of the polymer solution was adjusted to achieve LMPs volume fraction $(V_{LMPs}/(V_{LMPs} + V_{polymer}))$ of 25%, 50%, and 75%, enabling a comparison of resistance based on the volume fractions. Additionally, Hydrophilic PU (D4), PCL, PVA, PBAT, and SMP were dissolved in NMP at the same concentration of 0.1 g/ml at identical concentrations and subsequently combined with the LB-LMPs solution at a consistent LMPs volume fraction of 25%.

3.Authors employ Cabrera-Mott theory to explain reduction of oxide layer thickness. But why this theory is valid for Amines but not carboxyl groups? Also the from SI Figure 7, it is difficult to conclude that the thickness is lower for amine group. Moreover, what would happen if we change the NMP for instance to ethanol?

Answer. We appreciate the reviewer's insightful question regarding why organic acids containing carboxyl groups do not reduce the thickness of the oxide layer on liquid metal particles (LMPs). As the reviewer mentioned, carboxyl groups can inhibit oxidation reactions through chelation of metal ions, as explained by the Cabrera-Mott theory. However, the behavior of these acids on the surface of LMPs is more complex and can be understood through the Hard and Soft Acids and Bases (HSAB) theory. According to HSAB theory, carboxyl groups, being harder bases, have a higher affinity for the hard acid Ga^{3+} ions compared to softer bases like amine groups[1,2]. When carboxyl groups attach to the Ga^{3+} ions on the LMP surface, they can enhance the outward diffusion of Ga^{3+} ions. This outward diffusion promotes further oxide layer formation[3]. In contrast, the softer base, the amine group, is less likely to facilitate the outward diffusion of Ga^{3+} ions, thereby preventing further oxide layer formation. This distinction highlights the role of chemical interactions at the molecular level in determining the behavior of oxide layer formation on LMP surfaces.

Supplementary Fig. 7 presents data on the oxide layer thickness of LMPs synthesized by sonicating LMs with various ligands in cyclohexane. Measurements were conducted under three conditions: without a ligand, with carboxyl-functionalized ligands, and with amine-functionalized ligands. The results indicate that amine-functionalized ligands reduce the oxide thickness of LMPs by 28.5% compared to the condition without ligands. Additionally, as shown in the XPS analysis in Supplementary Fig. 9, the Ga^0 peak is significantly more prominent in LMPs treated with amine-functionalized ligands compared to those treated with carboxyl-functionalized ligands. This observation highlights the critical role of amine groups in mitigating oxide growth on the LMP surface.

The hydroxyl group of ethanol is considered less effective as a ligand due to its weaker electron pair donating ability compared to amine groups. XPS analysis of LMPs prepared via tip sonication in ethanol revealed a dominant Ga^{3+} peak over the Ga^0 peak, indicating that ethanol, when used as a ligand, does not effectively inhibit oxide layer formation on LMPs.

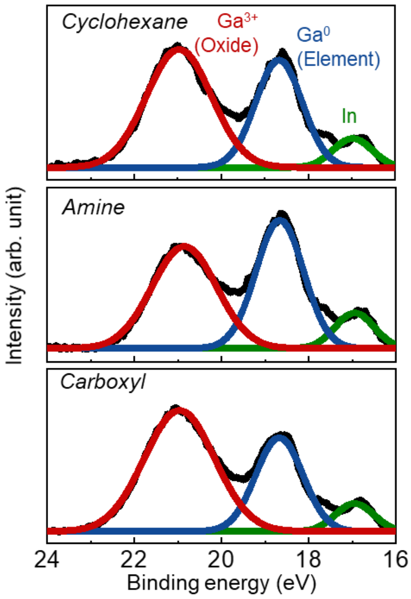

Supplementary Fig. 9| XPS of Ga 3d of LMPs depending to the functional groups of the ligand.

The red curve (~21 eV) corresponds the Ga^{3+} peak, indicative of the presence of gallium oxide, while the blue curve (18-19 eV) corresponds to the Ga^{0} peak.

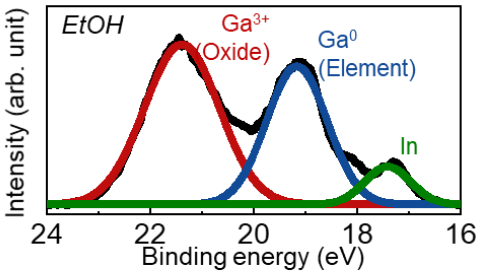

Fig| XPS of Ga 3d of LMPs treated with tip sonication in ethanol.

References

1 Pearson, Ralph G. Hard and soft acids and bases. *Journal of the American Chemical society*, **85(22)**, 3533-3539 (1963).

2 Pearson, Ralph G. Acids and Bases: Hard acids prefer to associate with hard bases, and soft acids prefer to associate with soft bases. *Science*, **151(3707)**, 172-177 (1966).

3 Castilla-Amoros, Laia, et al. Modulating the reactivity of liquid Ga nanoparticle inks by modifying their surface chemistry. *Journal of the American Chemical Society*, **144.4**, 1993-2001 (2022).

To provide additional information, we have revised the Supplementary Information section as detailed below:

Revised Supplementary Information

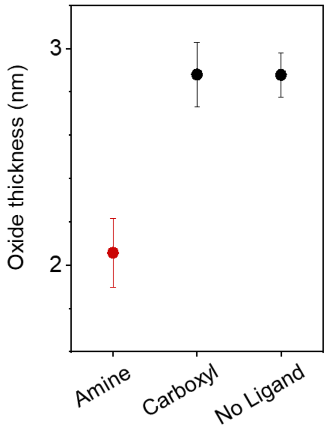

Supplementary Fig. 7| Oxide layer thickness of LMPs depending on the functional group of the ligand.

Each ligand bound LMP as synthesized by sonication in cyclohexane in the presence of the ligand. The ligand featuring an amine group reduce the oxide layer thickness of LMPs.

According to HSAB theory, carboxyl groups, being harder bases, have a higher affinity for the hard acid Ga^{3+} ions compared to softer bases like amine groups[5,6]. When carboxyl groups attach to the Ga^{3+} ions on the LMP surface, they can enhance the outward diffusion of Ga^{3+} ions. This outward diffusion promotes further oxide layer formation[2]. In contrast, the softer base, the amine group, is less likely to facilitate the outward diffusion of Ga^{3+} ions, thereby preventing further oxide layer formation. This distinction highlights the role of chemical interactions at the molecular level in determining the behavior of oxide layer formation on LMP surfaces.

4.Authors claim particle sintering is observed on figure 2f and 2g. However, I am not able to see such sintering. What authors call sintering? IN LMNPs, sintering haens when several particles join each other, eliminating the borders. Do authors have a different definition?

Answer. We appreciate the reviewer's question regarding particle sintering in Figures 2f and 2g and the need to clarify the meaning of "sintering" in this context.

We apologize the lack of a clear definition of sintering in the manuscript. In this study, sintering refers to the formation of conductive pathways between LMPs, where particle-to-particle connections are established, resulting in electrical conductivity. Figure 2g presents an optical image illustrating the macroscopic aggregation of liquid metal particles, while Figure 2f provides an SEM image emphasizing the sintering process. As shown in the SEM image in Figure 2f, LB-LMPs partially sinter as the solvent evaporates, forming connections with surrounding particles. In contrast, bare LMPs, constrained by their thick oxide layer, retain their spherical structure and do not establish such connections.

Furthermore, the initial conductivity observed in the bottom layer of the LB-LMPs/polymer film supports that sintering occurs, enabling the formation of conductive pathways. This phenomenon is further evidenced by the cross-sectional SEM image of the LB-LMPs/polymer film in Figure 3a, which highlights the interconnected network of sintered particles. Conversely, the bottom layer of the bare LMPs/polymer film has no initial conductivity, corroborating the absence of sintering.

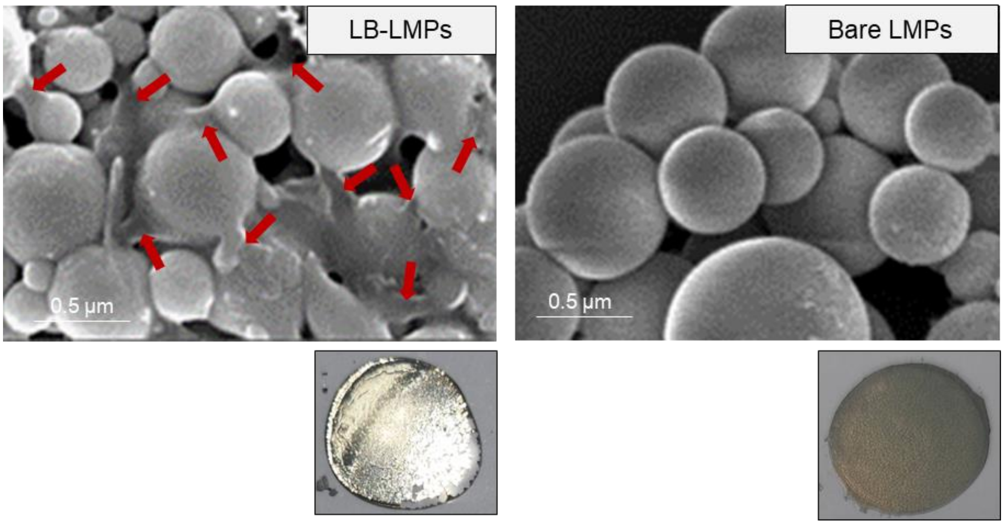

Fig. 2 Mechanism of in situ sintering and self-packaging of LB-LMPs.
f, SEM images of the top surfaces of bare LMPs and LB-LMPs.

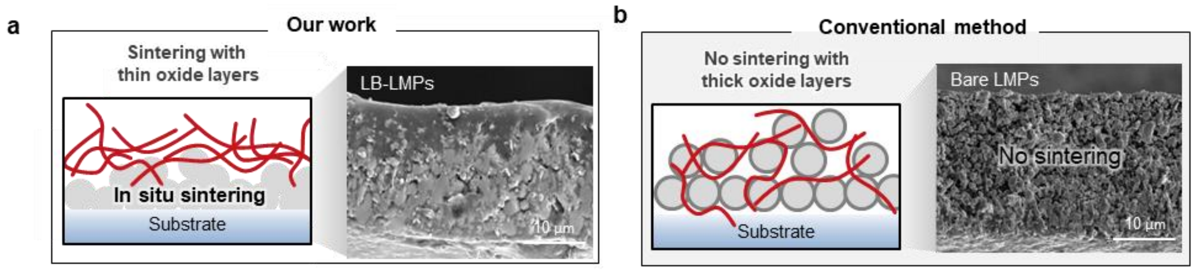

Fig. 3 Mechanical and electrical properties of LB-LMPs-based conductors.
a, Schematic illustration and cross-sectional SEM image of the LB-LMPs/polymer film and **b**, the bare LMPs/polymer film.

5.Also in figure s10, I don't see any sintering. Also why the sizes of particles are different? Why LMP with no NMP has smaller size? Were they sonicated same time?

Answer. We appreciate the reviewer's questions regarding the observed particle size differences and sonication conditions in Figure S10.

Bare LMPs and LB-LMPs were prepared using different tip sonication methods tailored to their respective properties. Bare LMPs were tip sonicated in NMP while immersed in an ice bath to minimize temperature rise, whereas LB-LMPs were tip sonicated without an ice bath to facilitate ligand formation from NMP.

To further investigate the reviewer's concerns about particle size, we analyzed the sizes of LB-LMPs and bare LMPs using SEM imaging under their respective sonication conditions. The SEM results revealed that the particle sizes of LB-LMPs ranged from approximately 0.1 to 2.5 µm, whereas bare LMPs exhibited particle sizes of approximately 0.1 to 1.5 µm. This represents a size difference of up to 2 times between the two systems.

The larger particle size of LB-LMPs can be attributed to the reduction in cavitation energy at elevated temperatures, which influences the fragmentation behavior during sonication. As the temperature increases, the vapor pressure of the liquid rises, leading to vapor-filled bubble formation during tip sonication. These vapor-filled bubbles collapse less violently due to the reduced pressure differential between the bubble interior and the surrounding liquid[1,2]. Although more bubbles may form, the energy released during each cavitation is lower, resulting in diminished overall cavitation intensity. Consequently, when tip sonication is conducted without an ice bath, the LMP particles tend to exhibit relatively larger sizes due to reduced fragmentation efficiency.

The sintering of the LB-LMP/TPU film is demonstrated through the cross-sectional SEM image (Fig. 3a). In Supplementary Fig. 10, although the backside of the LB-LMPs/polymer film appears to be separated by the polymer matrix, the LMPs are vertically interconnected, as evidenced in Fig. 3a, leading to high electrical conductivity.

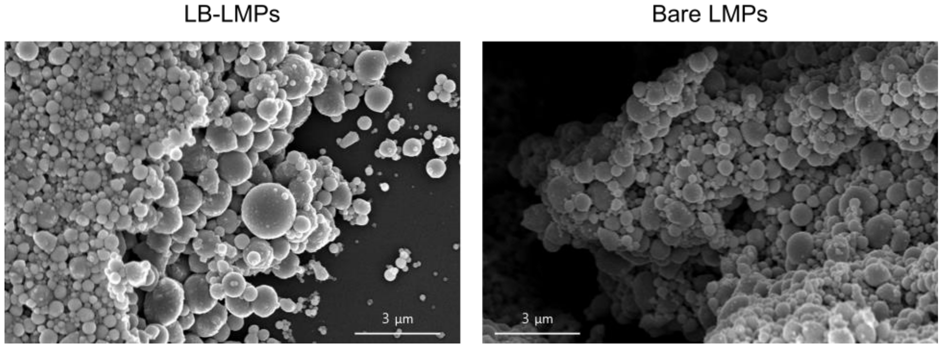

Fig| SEM images of LB-LMPs and bare LMPs

References

1 Ruiz, Esneyder, et al. Study of sonication parameters on PLA nanoparticles preparation by simple emulsion-evaporation solvent technique. *European Polymer Journal*, **173**, 111307 (2022).

2 Siddiqui, Akhtar, et al. Modeling the effect of sonication parameters on size and dispersion temperature of solid lipid nanoparticles (SLNs) by response surface methodology (RSM). *Pharmaceutical development and technology*, **19.3,** 342-346 (2014).

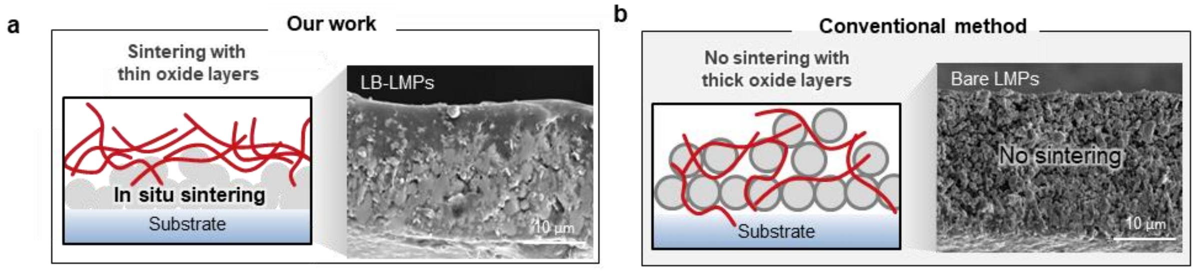

Fig. 3 Mechanical and electrical properties of LB-LMPs-based conductors.
a, Schematic illustration and cross-sectional SEM image of the LB-LMPs/polymer film and **b**, the bare LMPs/polymer film.

6.Figure s13 is a very nice figure, but should be replicared for LB-LMB

Answer. We appreciate your comment regarding Supplementary Fig. 14. We would like to clarify that the SEM images presented in Supplementary Fig. 14 were indeed obtained using films made from LB-LMPs. We recognize that the lack of clear mention in the manuscript may have caused confusion. To address this, we have revised the figure legend and the corresponding text to clearly specify that the data pertains to LB-LMP films.

Revised Supplementary Information

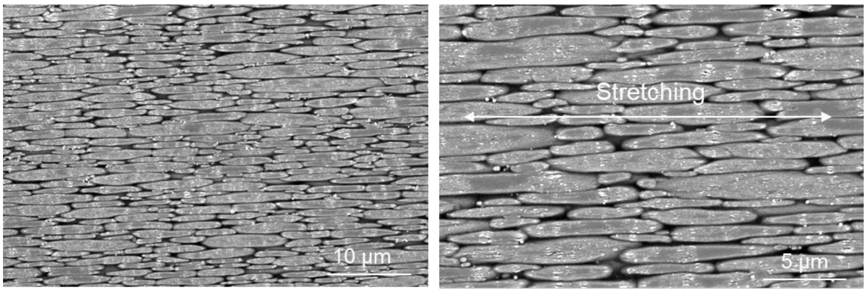

Supplementary Fig. 14| SEM images on the bottom surface of the stretched LB-LMPs/polymer films.

As LMPs elongate during stretching, their length increases; however, due to the simultaneous increase in contact area between the particles, there is only minimal change observed in resistance[5].

7.The step-by-step and in detail protocol for measurement of conductivity on LB-LMP should be explained. What substrate is used? How the deposition is made, and how the conductivity is measured. For instance a measurement over glass or silicon wafer with no peeling can be interesting.

Answer. We appreciate the Reviewer's comment regarding the measurement of conductivity.

To precisely measure the conductivity of our stretchable conductor, we focused on the conductive layer. Previously, the backside of the polymer was measured by detaching it through immersion in water, allowing the backside to be released without any mechanical stimuli. Conductivity measurements were performed using a four-point probe setup (Keithley 2400).

Since our film is covered with TPU, measuring conductivity directly on the silicon wafer is limited. Instead, we measured the conductivity of patterned lines with vertically connected AgF without peeling and included this data in the Supplementary Information.

To ensure a more precise explanation of our approach, we have revised the Methods section of the main text as outlined below:

Revised Main Text

Electrical characterization. The electrical properties of the conductor were measured using an LCR meter (4284 A, HP) and a source meter unit (2450, Keithley). To measure the resistance of the backside of the LMPs/polymer film, the film was detached through water immersion, allowing for separation without applying mechanical force. To monitor resistance variation under strain, a special setup including a force gauge (with a maximum force of 50 N, Mark-10), a stand with a motor (Mark-10), and a customized manual strain machine were utilized.

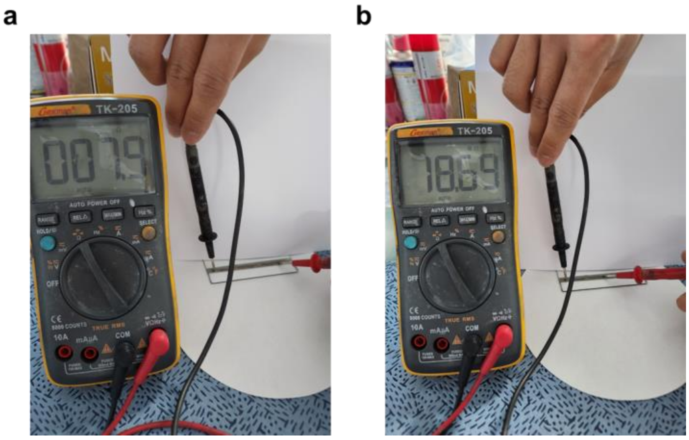

Supplementary Fig. 23| AgF-based selective electrical connection. a, Resistance between AgF connection (7.9 Ohm). **b,** Resistance between AgF and a packaged layer (18.69 Ohm).

8.“In contrast, bare LMPs with thick oxide layers exhibit only a marginal descent due to their high density” I didn’t understand.

Answer. We appreciate the reviewer’s thoughtful comment. Thank you for the opportunity to clarify this point.

We recognize that this sentence may have caused confusion and have revised the manuscript to provide greater clarity. Our intention was to highlight the difference between bare LMPs and LB-LMPs in terms of phase separation. Due to the relatively high intrinsic density of LMPs compared to the polymer, a small amount of bare LMP may precipitate during solvent evaporation. However, achieving complete phase separation requires particle sintering. In the case of bare LMPs, the thick oxide layer inhibits particle-to-particle sintering, limiting precipitation and preventing effective separation. In contrast, LB-LMPs, functionalized with ligands that reduce the oxide layer thickness, exhibit efficient particle-to-particle sintering. This sintering allows the LB-LMPs to fully sink to the bottom during the evaporation process, resulting in distinct phase separation and the formation of an integrated layer that is entirely separate from the polymer matrix.

To avoid potential misinterpretation, we have removed the original sentence and revised the discussion to better reflect these distinctions. Thank you again for pointing this out and allowing us to improve the manuscript.

Revised Main Text

This vertical phase separation effectively prevents the leakage of liquid metal while maintaining initial conductivity, eliminating the need for an additional activation and packaging processes. In contrast, bare LMPs with thick oxide layers failure to sinter or achieve complete phase separation in the polymer matrix (**Fig. 3b, Supplementary Fig. 10**).

9.Authors mentioned "Moreover, fabricating the LB-LMPs/polymer film at ambient temperature without thermal treatment precludes ligand detachment, thereby impeding sintering50-52 between LMPs and inhibiting phase separation (Supplementary Fig. 11). This suggests that evaporation-induced desorption of ligands from LMPs is crucial to facilitate interparticle sintering among LMPs.". However, I didn't find detailed information about such thermal treatment. Do author refer to protocol of preparation of particles? Is the solution heated? To what temperature. When the polymer is added? Once more these details are necessary to make the work reproducible.

Answer. Thank you for your question and for highlighting the need for more detailed information regarding the thermal treatment process in the fabrication of LB-LMPs/polymer films.

The LB-LMPs/polymer films were fabricated by evaporating the solvent NMP from the LB-LMPs/polymer ink at a controlled temperature of 60°C. The polymer was included in the ink from the beginning of the process (Supplementary Fig. 2). During the solvent evaporation at 60°C, evaporation-induced desorption of ligands from the LMPs occurs, which facilitates particle-to-particle sintering among the LMPs. This sintering drives the vertical phase separation, where the LMP phase sinks downwards while the polymer phase separates upwards.

We hope this clarification addresses your concerns and provides the necessary details for reproducibility. To ensure a more precise explanation of our approach, we have revised the Methods section of the main text as outlined below:

Revised Main Text

The volume of the polymer solution was adjusted to achieve LMPs volume fraction $(V_{LMPs}/(V_{LMPs} + V_{polymer}))$ of 25%, 50%, and 75%, enabling a comparison of resistance based on the volume fractions. Additionally, Hydrophilic PU (D4), PCL, PVA, PBAT, and SMP were dissolved in NMP at the same concentration of 0.1 g/ml at identical concentrations and subsequently combined with the LB-LMPs solution at a consistent LMPs volume fraction of 25%. LB-LMPs/polymer films were prepared by evaporating the solvent from the LB-LMPs/polymer ink using a hotplate set at 60°C.

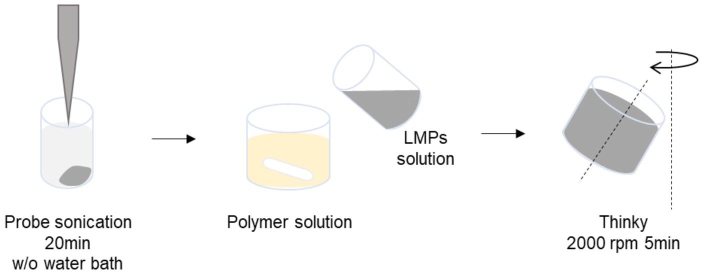

Supplementary Fig. 2| Fabrication of LB-LMPs/polymer ink.

Bulk LM was immersed in NMP, subjected to ultrasonication, and subsequently mixed through rotational agitation with the polymer solution.

10.Authors mentioned "for high electrical conductivity, achieving 1 Ω in the interconnect without requiring activation, even at small polymer-to-LMPs volume ratios ". Why do you mean by even at small polymer-to-LMP. Did you mean even at Larger Polymer to-LMPs? Naturally by adding polymer, sintering becomes more difficult. So this sentence doesn't make much sense. Also what are these ratios? Again details are missing.

Answer. We appreciate the reviewer's question and the opportunity to clarify this point. We apologize for the confusion. The term "polymer-to-LMPs volume ratios" was indeed misleading, and it has been replaced with "LMPs volume fraction" for accuracy. The LMPs volume fraction is calculated using the following formula:

$$LMPs\ \ volyme\ \ fraction(\%) = (V_{LMPs}/V_{LMPs} + V_{polymer})\ \ \text{x}\ \ 100$$

We agree that adding polymer makes sintering more challenging due to the increased insulation, which is why achieving high electrical conductivity even at lower LMPs volume fractions is significant.

To provide a clearer explanation of our approach, we have revised the Figure 3d and Methods section of the main text as detailed below:

Revised Main Text and Figure

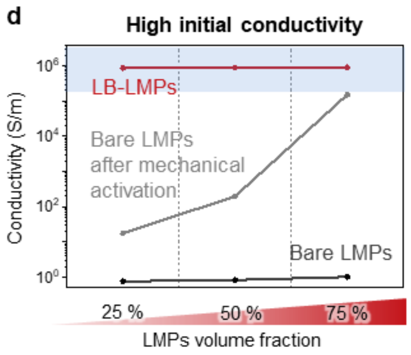

Fig. 3 Mechanical and electrical properties of LB-LMPs-based conductors.
d, Conductivity of bare LMPs and LB-LMPs-based conductors according to polymer contents

This suggests that evaporation-induced desorption of ligands from LMPs is crucial to facilitate interparticle sintering among LMPs. Notably, sintering of particles allows for high electrical conductivity, achieving 1 Ω in the interconnect without requiring activation, even at small LMPs volume ratios (Fig. 3d).

…

Preparation of LB-LMPs/polymer ink. The LB-LMPs solution was incorporated into a polymer solution (1.2g of TPU in 18mL of NMP), undergoing mixing for a duration of 5 minutes utilizing a Thinky mixer (AR-100). The volume of the polymer solution was adjusted to achieve LMPs volume fraction $(V_{LMPs}/(V_{LMPs} + V_{polymer}))$ of 25%, 50%,

and 75%, enabling a comparison of resistance based on the volume fractions. Additionally, Hydrophilic PU (D4), PCL, PVA, PBAT, and SMP were dissolved in NMP at the same concentration of 0.1 g/ml at identical concentrations and subsequently combined with the LB-LMPs solution at a consistent LMPs volume fraction of 25%.

11.Authors mention: “Furthermore, by tuning the viscosity of ink through varying polymer contents, various solution process like printing or dip coating become available (Supplementary Fig. 16).” Once more details of each formulation is missing. If authors claim screen printing, dip coating, etc., for EACH method, they need to write down how the ink is prepared step by step, with details of formulation and preparation.

Answer. Thank you for your question regarding the details of the ink formulations for different fabrication techniques. We appreciate the opportunity to provide a more thorough explanation.

All inks were prepared with a fixed LB-LMPs volume fraction of 25%. As shown in Supplementary Fig. 17, the viscosity of the LB-LMPs/polymer ink was adjusted by varying the polymer concentration to enable its use across multiple fabrication methods.

1. For screen printing, the ink was prepared by dissolving 0.6 g of TPU in 12 ml of NMP to create the polymer solution.
2. For dip coating, the polymer solution was adjusted to 0.6 g of TPU dissolved in 9 ml of NMP.
3. For nozzle printing, the polymer solution was prepared by dissolving 0.6 g of TPU in 6 ml of NMP.

In all cases, the LB-LMPs solution was prepared separately by performing tip sonication on 1.2 g of LM in 3 ml of NMP. The polymer solution and the LB-LMPs solution were then combined and mixed thoroughly using a Thinky mixer to obtain the final ink formulation for each fabrication method. These specific formulations ensure that the ink viscosity is optimized for each respective technique. To provide a more detailed and accurate explanation of our approach, we have revised the Methods section of the main text as described below:

Revised Main text

We conducted screen printing, nozzle printing, and dip coating with LB-LMPs/polymer ink by adjusting the viscosity of the ink. The viscosity of the ink was adjusted by varying the polymer concentration. For screen printing, the ink was formulated by dissolving 0.6 g of TPU in 12 ml of NMP to create the polymer solution. For dip coating, the polymer solution consisted of 0.6 g of TPU dissolved in 9 ml of NMP. For nozzle printing, the polymer solution was prepared by dissolving 0.6 g of TPU in 6 ml of NMP. In all cases, the LB-LMPs solution was prepared separately by performing tip sonication on 1.2 g of LM in 3 ml of NMP, followed by thorough mixing with the polymer solution using a Thinky mixer.

12.I didn’t find detailed information on preparation of the Ag flake ink for VIAs.

Answer. We appreciate the reviewer’s question regarding the preparation of the Ag flake ink for VIAs and thank you for pointing out the missing details in the manuscript. The AgF ink was prepared by adding 2 g of AgF to a TPU solution composed of 0.8 g of TPU dissolved in 12 ml of NMP. The mixture was then thoroughly homogenized using a vortex mixer to ensure uniform dispersion of the Ag flakes in the solution. To offer a clearer explanation of our approach, we have updated the Methods section of the main text as outlined below:

Revised Main text

To establish vertical electrical connections with the top polymer layer, AgF or PEDOT:PSS ink was deposited onto the printed LB-LMPs/polymer ink. The AgF ink was prepared by adding 2 g of AgF to a TPU solution composed of 0.8 g of TPU dissolved in 12 ml of NMP. The PEDOT:PSS ink was prepared by dissolving 0.3 g of TPU and 0.15 g of PEDOT:PSS in 15 ml of NMP.

13.Some materials do nto have reference. For instance “silicone adhesive film” , “release tape” “Ag flakes”

Answer. We appreciate the reviewer’s observation regarding missing references for certain materials. The silicone adhesive film used in our experiments is VHB tape (3M), the release tape is Water-Soluble Wave Solder Tape 5414 sourced from 3M, and the Ag flakes (0.5~2 μm) were sourced from Daejoo Electronics Materials Co. These details are included in the revised manuscript to ensure proper attribution and clarity for readers.

Revised Main text

LM alloy (eutectic gallium indium, EGaIn) was purchased from Changsha Ruichi Nonferrous Metals, and Ag flakes (0.5~2 μm) were obtained from Daejoo Electronics Materials Co.

…

For printing the substrate, PDMS (Sylgard 184, Dow Corning), VHB tape (3 M), Kapton tape (3 M) and Tegaderm (3 M) were used. Water-Soluble Wave Solder Tape 5414 (3M) was used as release tape.

14.Protocol and details of film removal through release film is not described

Answer. We appreciate the reviewer's observation regarding the lack of details on the film removal process using the release film. To clarify, the protocol for transferring high-precision circuits involves the following steps:

The screen-printed circuit is first soaked in water for 30 minutes. This step allows water to penetrate between the substrate and the film, reducing adhesive strength and making the film easier to remove. Next, a release tape is carefully attached to the film, and the film is detached from the glass substrate without applying peeling force. The release tape, now holding the film, is then adhered to the VHB tape. Finally, the release process facilitates adhesion to the VHB tape while ensuring the film remains intact and undistorted.

The method employing LB-LMPs facilitates vertical phase separation, achieving conductivity even at low LMP volume ratios. A low LMP volume ratio corresponds to a relatively high polymer content, which enhances mechanical stability, a critical factor for the transfer process. Our self-packaged LB-LMP/polymer films demonstrate exceptional mechanical stability, effectively preventing mechanical damage and liquid metal leakage during transfer.

To provide a clearer explanation of our approach, we have revised the Supplementary Information section of the main text as outlined below:

Revised Supplementary Information

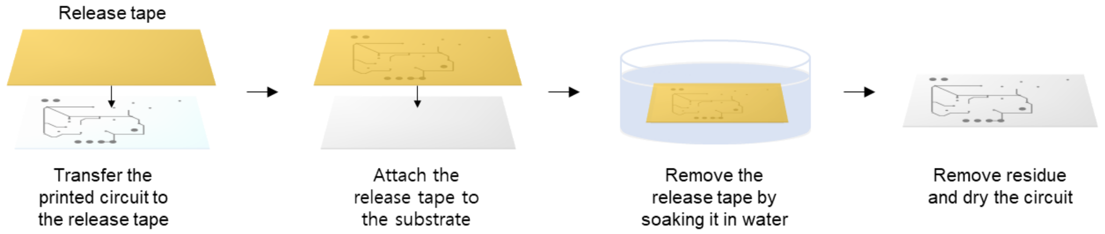

Supplementary Fig. 29| Transfer Process for S-PCB Fabrication.

Printed circuits were immersed in water, allowing for easy removal using release tape. Each printed circuit layer was then sequentially transferred onto the target substrate, enabling the assembly of multi-layer S-PCBs.

15.Authors mention “To obtain the bottom surface images of the LMPs/polymer films, the films underwent immersion in water for 10 minutes, and were then peeled off without tension”. Peeling without tension might be difficult to achieve. Why not despoiting over glass and obtaining optical image?

Answer. We appreciate the reviewer's insightful comments on the imaging method for the bottom surface of the LB-LMP/polymer film and have adopted the approach suggested.

To verify this, LB-LMP/polymer ink was poured onto a glass plate, and the solvent was evaporated to create an LB-LMP/polymer film. The bottom surface of the film was then optically imaged without detaching it from the glass plate. This approach ensured stability and preserved the structure of the film during imaging. The optical images confirmed that the bottom layer of the film displayed a dense distribution of LMPs, while the top layer was effectively packaged with polymer.

.

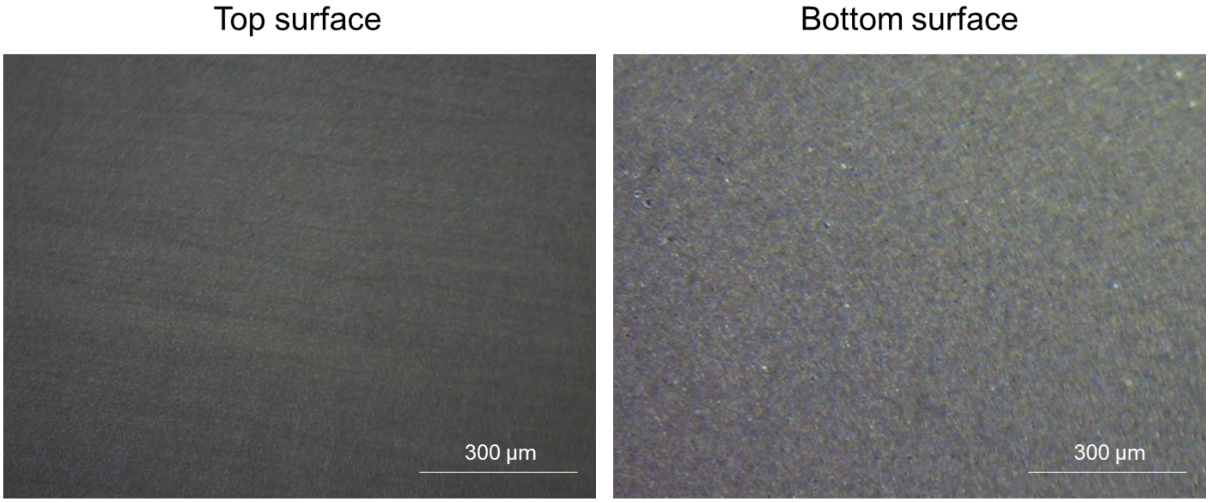

Fig| Optical images of top surface and bottom surface of LB-LMPs/TPU film

16.Actual conductivity in S/M should be reported by measuring sample conductivity and thickness of the printed lines.

Answer. We appreciate the Reviewer's comment regarding conductivity.

As suggested, we have updated our data to use the S/m unit for consistency and clarity. To ensure accurate conductivity measurements, we determine the thickness of the conductive layer by analyzing SEM images and estimating its dimensions. Our sample exhibits a conductivity of approximately 9×10^5 S/m, which is comparable to the values reported for previous LMP-based conductors.

Revised Figure

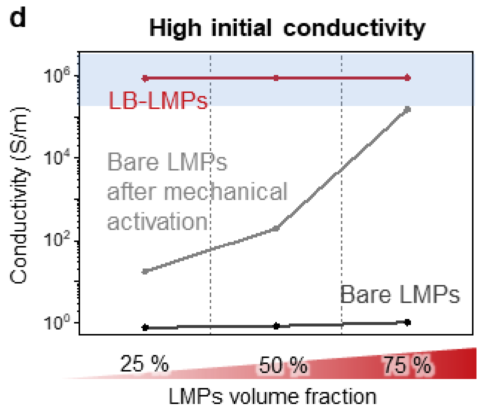

Fig. 3 Mechanical and electrical properties of LB-LMPs-based conductors.
d, Conductivity of bare LMPs and LB-LMPs-based conductors according to polymer contents
